# MICFOA: A Novel Improved Catch Fish Optimization Algorithm with Multi-Strategy for Solving Global Problems

**DOI:** 10.3390/biomimetics9090509

**Published:** 2024-08-23

**Authors:** Zhihao Fu, Zhichun Li, Yongkang Li, Haoyu Chen

**Affiliations:** 1 School of Electronic Information Engineering, Hankou University, Wuhan 430212, China; fuzhihao@hkxy.edu.cn (Z.F.); 2062040051@hkxy.edu.cn (Y.L.); 2 Department of Health Technology and Informatics, The Hong Kong Polytechnic University, Hong Kong 999077, China; zhichun20.li@connect.polyu.hk; 3 College of Petroleum Engineering, Xi’an Shiyou University, Xi’an 710065, China

**Keywords:** catch fish optimization algorithm, Lévy flight, weight-balanced selection mechanism, global optimization, CEC 2018 test suite, CEC 2022 test suite

## Abstract

Catch fish optimization algorithm (CFOA) is a newly proposed meta-heuristic algorithm based on human behaviors. CFOA shows better performance on multiple test functions and clustering problems. However, CFOA shows poor performance in some cases, and there is still room for improvement in convergence accuracy, getting rid of local traps, and so on. To further enhance the performance of CFOA, a multi-strategy improved catch fish optimization algorithm (MICFOA) is proposed in this paper. In the exploration phase, we propose a Lévy-based differential independent search strategy to enhance the global search capability of the algorithm while minimizing the impact on the convergence speed. Secondly, in the exploitation phase, a weight-balanced selection mechanism is used to maintain population diversity, enhance the algorithm’s ability to get rid of local optima during the search process, and effectively boost the convergence accuracy. Furthermore, the structure of CFOA is also modified in this paper. A fishermen position replacement strategy is added at the end of the algorithm as a way to strengthen the robustness of the algorithm. To evaluate the performance of MICFOA, a comprehensive comparison with nine other metaheuristic algorithms is performed on the 10/30/50/100 dimensions of the CEC 2017 test functions and the 10/20 dimensions of the CEC2022 test functions. Statistical experiments show that MICFOA has more significant dominance in numerical optimization problems, and its overall performance outperforms the CFOA, PEOA, TLBO, COA, ARO, EDO, YDSE, and other state-of-the-art algorithms such as LSHADE, JADE, IDE-EDA, and APSM-jSO.

## 1. Introduction

There are many optimization problems in the real world that need to be solved using artificial intelligence techniques [1]. Optimization methods are a branch of AI. The metaheuristic algorithm is an efficient stochastic optimization method [2]. Compared with the limitations of traditional mathematical methods in solving optimization problems with high dimensionality, multiple constraints, no gradient information, and the existence of a large number of locally optimal solutions [3], metaheuristic algorithms, with their simplicity, flexibility, and no gradient information required, have been widely noticed and applied in different fields [4,5,6,7]. Metaheuristic algorithms generate multiple initial solutions randomly in the problem space and iterate over the initial solutions by using a specific update formula to provide a more satisfactory solution within an acceptable time cost. For metaheuristic algorithms, their performance depends on how to balance local and global search. Therefore, metaheuristic algorithms usually switch from global exploration to local exploitation as a way to find the global optimal solution. In other words, metaheuristic algorithms reduce the dependence on the location of the historical optimal solution in the exploration phase and expand the search scope as much as possible. In the exploitation phase, they increase the dependence on the historical optimal solution location and dig as deep as possible into a certain problem area.

Since their emergence, a variety of metaheuristic algorithms derived from different inspirations have been proposed, which have been inspired by different animal behaviors, plant growth mechanisms, human social behaviors, and a wide range of physicochemical phenomena. Based on the source of inspiration, metaheuristic algorithms can be broadly categorized into four groups [8]: evolution-based algorithms, physics-based algorithms, swarm-based algorithms, and human-based algorithms.

The history of metaheuristic algorithms is rooted in evolution-based algorithms. The Genetic Algorithm (GA) and Differential Evolution (DE) are the best-known evolution-based algorithms. GA is an algorithm proposed by John Henry Holland through modeling the natural selection mechanism of Darwin’s evolutionary theory and the genetics mechanism of the biological evolutionary process [9]. Different from GA, DE pushes the population towards a global optimal solution by using mutation, crossover, and selection operators in each generation [10]. As an efficient metaheuristic algorithm with a simple structure, DE has been continuously improved by a broad range of researchers. JADE, as an adaptive DE algorithm with external archiving, is an important variant in the history of DE development [11]. LSHADE is a successful DE variant that scored well at the IEEE Conference on Evolutionary Computation in 2014 [12]. IDE-EDA is an excellent DE variant with a hybrid distribution estimation algorithm [13]. APSM-jSO [14] is a novel high-performance variant of jSO, and jSO [15] is an excellent algorithm proposed based on LSHADE. Except for GA and DE, evolution-based algorithms also include Evolutionary Strategies (ES) [16], Evolutionary Programming (EP) [17], and Genetic Programming (GP) [18].

Physics-based algorithms are inspired by various physical or chemical phenomena in nature. For example, Simulated Annealing (SA) is inspired by the annealing process of solid materials [19]. The RIME achieves the optimization process by mimicking the crossover behavior between ice [20]. The Equilibrium Optimizer (EO) [21] is based on the principle that particles update their positions according to the candidate solutions in the created equilibrium pool within a controlled volume-mass equilibrium model. Other physics-based algorithms include the Gravitational Search Algorithm (GSA) [22], Lightning Search Algorithm (LSA) [23], Energy Valley Optimizer (EVO) [24], and Kepler Optimization Algorithm (KOA) [25].

Swarm-based algorithms are the most numerous category of metaheuristic algorithms. These algorithms are inspired by the foraging behavior, survival behavior, cooperative behavior, reproductive behavior, and migratory behavior of different kinds of animals. The classical swarm-based algorithms include Particle Swarm Optimization (PSO) [26], Ant Colony Optimization (ACO) [27], Artificial Bee Colony (ABC) [28], Cuckoo Search (CS) [29], and Firefly Algorithm (FA) [19]. In recent years, several novel swarm-based algorithms have been proposed, including the Whale Optimization Algorithm (WOA) [30], Dwarf Mongoose Optimization (DMO) [31], Butterfly Optimization Algorithm (BOA) [32], Golden Jackal Optimization (GJO) [33], Tuna Swarm Optimization (TSO) [34], Remora Optimization Algorithm (ROA) [35], and Fennec Fox Algorithm (FFA) [36].

The human-based algorithm is a novel category of metaheuristic algorithms. One of the better-known algorithms in this category is Teaching Learning Based Optimization (TLBO) [37], which is inspired by the teaching-learning behavior between teachers and students. Additionally, this category of algorithms also includes the Brain Storm Optimization Algorithm (BSOA) [38], Socio Evolution and Learning Optimization (SELO) [39], Group Teaching Optimization Algorithm (GTOA) [40], Political Optimizer (PO) [41], Most Valuable Player Algorithm (MVPA) [42], and Skill Optimization Algorithm (SOA) [43].

Many great metaheuristic algorithms have logically demonstrated that there is no metaheuristic algorithm that is best suited to solve all optimization problems, that is, there is a No Free Lunch (NFL) theory [44]. In other words, a particular metaheuristic algorithm may show very promising results on one set of problems, but the same algorithm may perform poorly on another set of problems. Obviously, NFL has made this area of research very active, so that new metaheuristics and improvements to existing methods are proposed every year.

Catch fish optimization algorithm (CFOA) is a human-based meta-heuristic algorithm proposed by Jia et al. in 2024 to mimic the catching behavior of fishermen. With the advantages of few parameters and a simple structure, CFOA has been proved to be superior to DE, GA, PSO, GWO, WOA, and other classical meta-heuristic algorithms, and has strong optimization ability in test functions and clustering problems. However, CFOA also has some problems, such as the lack of balance between exploitation and exploration behaviors and insufficient population diversity. Furthermore, since the algorithm has just been proposed, no other researcher has yet conducted an in-depth study on CFOA. Thus, we firstly propose the multi-strategy improved catch fish optimization algorithm (MICFOA) for the purpose of enhancing the performance of CFOA.

The primary contributions of this study are as follows:

- A Lévy-based differential independent search strategy was introduced in the exploration phase to enhance the global search capability of the algorithm while minimizing the impact on convergence speed;
- A weight-balanced selection mechanism was introduced in the exploitation phase to maintain population diversity, enhance the algorithm’s ability to get rid of local optima during the search process, and effectively boost convergence accuracy;
- Refining the algorithm structure and using a fishermen position replacement strategy to strengthen the robustness of the algorithm;
- The performance of MICFOA was comprehensively evaluated using two benchmark test sets: CEC2017 and CEC2022.

The rest of the paper is structured as follows: Section 2 briefly introduces the catch fish optimization algorithm. In Section 3, the improvement strategies and the proposed MICFOA are separately presented. In Section 4, we evaluate the performance of MICFOA using functions with different dimensions. Finally, Section 5 provides conclusions and an outlook for future work.

## 2. Catch Fish Optimization Algorithm

### 2.1. Inspiration for CFOA

Catch fish optimization algorithm is a metaheuristic algorithm inspired by the catching behavior of fishermen in ponds. Fishermen will rely on their own knowledge and intuition when catching fish, and they are also able to cooperate with other fishermen in catching fish with their tools. Moreover, fishermen will work together to salvage the fish after surrounding them.

### 2.2. Mathematical Model for CFOA

#### 2.2.1. Initialization Process

The populations in the CFOA initialization process are randomly generated as in many metaheuristic algorithms. Assuming that there are N fishermen in the D-dimensional search space, the population matrix of fishermen can be represented by mathematical Equation (1).

(1) $$X=\left[\begin{array}{c} X_{1}\\ X_{2}\\ \vdots \\ X_{i}\\ \vdots \\ X_{N} \end{array}\right]=\left[\begin{array}{cccccc} x_{1,1} & x_{1,2} & \cdots & x_{1,j} & \cdots & x_{1,D}\\ x_{2,1} & x_{2,2} & \cdots & x_{2,j} & \cdots & x_{2,D}\\ \vdots & \vdots & \ddots & \vdots & \ddots & \vdots \\ x_{i,1} & x_{i,2} & \cdots & x_{i,j} & \cdots & x_{i,D}\\ \vdots & \vdots & \ddots & \vdots & \ddots & \vdots \\ x_{N,1} & x_{N,2} & \cdots & x_{N,j} & \cdots & x_{N,D} \end{array}\right]$$

As previously noted, every fisher is a potential solution for the problem, and by utilizing its proposed values for the decision variables, the problem’s objective function can be assessed. Equation (2) symbolizes the calculated value of the objective function derived from the fishers.

(2) $$F\left(X\right)=\left[fit_{1},fit_{2},\ldots ,fit_{N}\right]=\left[function\left(X_{1}\right),function\left(X_{2}\right),\ldots ,function\left(X_{N}\right)\right]$$

In the equation, $fit_{i}$ and $function\left(X_{i}\right)$ represent the objective function value assessed for the *i*th fisher. Each $X_{i}$ represents a fisher position, which is initialized in the following representation:

(3) $$X_{i,j}=lb_{j}+rand\times \left(ub_{j}-lb_{j}\right),i=1,2,\ldots ,N$$

where $X_{i,j}$ is the position of the *i*th fisher in the *j*th dimension. $ub_{j}$ and $lb_{j}$ denote the upper and lower boundaries of the *j*th dimension in the problem space. Metaheuristic algorithms usually contain two phases: exploration and exploitation. In CFOA, the switch from exploration to exploitation is controlled using the parameter *S*. When *S* < 0.5, CFOA performs the exploration behavior; vice versa, CFOA performs the exploitation behavior. The calculation of the parameter *S* can be obtained from Equation (4).

(4) $$S=\frac{t}{T}$$

where *t* and *T* denote the current iteration number and the maximum iteration number.

#### 2.2.2. Independent Search and Group Capture

CFOA has two search methods in the exploration phase: independent search and group capture. The fisherman will choose one search method for catching fish in each iteration and switch between the two methods by the catch rate parameter *α*. The parameter *α* can be obtained from Equation (4).

(5) $$\alpha ={\left(1-\frac{3}{2}S\right)}^{\frac{3}{2}S}$$

When the catch rate α is higher (i.e., α>p), fishermen choose to catch fish by independent search. Fishermen will disturb the water while fishing, forcing fish to poke out of the water to breathe. The fishermen will recognize the location of the fish by observing the ripples produced by the fishes’ breathing. At the same time, the fisherman would correct his position based on the catch of others. The strategy is updated as shown below.

(6) $$X_{i,j}\left(t+1\right)=X_{i,j}\left(t\right)+\left(X_{r,j}\left(t\right)-X_{i,j}\left(t\right)\right)\times Exp+rand\times s_{j}\times R$$

(7) $$Exp=\frac{fit_{i}-fit_{r}}{fit_{\max}-fit_{\min}}$$

(8) $$R=dis\times \sqrt{Exp}\times \left(1-S\right)$$

where $X_{r,j}\left(t\right)$ denotes a random selection of fisher locations different from $X_{i,j}\left(t\right)$. *Exp* is the experience obtained by the *i*th fisherman $X_{i,j}\left(t\right)$ using $X_{r,j}\left(t\right)$ as the reference person. $fit_{i}\left(i=1,2,\ldots ,N\right)$ denotes the fitness of each fisher. $fit_{\max}$ and $fit_{\min}$ represent the maximum and minimum fitness at the current number of iterations. *dis* is the Euclidean distance between the *i*th fisherman $X_{i}\left(t\right)$ and the reference point $X_{r}\left(t\right)$. *s* is a random unit vector with dimension *D*.

When α≤p, the fisherman chooses group capture. The fisherman cooperates with other fishermen to expand their fishing capacity by using nets. Generally, three or four fishermen will cooperate in fishing. The strategy is represented as follows:

(9) $$X_{i,j}\left(t+1\right)=X_{i,j}\left(t\right)+rand\times \left(Center_{c}-X_{c,j}\left(t\right)\right)+{\left(1-2S\right)}^{2}\times r_{1}$$

(10) $$Center_{c}=mean\left(X_{c}\left(t\right)\right)$$

where $Center_{c}$ is the target point of the group c encirclement. c denotes a group of 3 or 4 individuals. $r_{1}$ is a random number ranging from −1 to 1. $mean\left(X_{c}\left(t\right)\right)$ denotes the average position of $X_{c}\left(t\right)$.

#### 2.2.3. Collective Capture

In the later stages of catching fish, all fishermen move under a unified search pattern, driving free and hidden fish to the same spot for gathering and encirclement. The distribution of fishermen is centered on the school of fish, with the degree of aggregation gradually thinning from the middle to the periphery, and the range of distribution gradually narrowing outward. The center fishermen catch the school of fish, and the peripheral fishermen catch the escaped fish. The updated equation is as follows.

(11) $$X_{i}\left(t+1\right)=X_{Gb}+normrnd\left(0,\frac{r_{2}\times \sigma \times \left|mean\left(X\right)-X_{Gb}\right|}{3}\right)$$

(12) $$\sigma =\sqrt{\left(\frac{2\times \left(1-S\right)}{{\left(1-S\right)}^{2}+1}\right)}$$

where $X_{Gb}$ is the position of the fisher with the best fitness value. GD is a Gaussian distribution function that follows a mean of 0 and a standard deviation of σ. $r_{2}$ is a random integer with values between 1 and 3. $\left|\cdot \right|$ indicates that absolute values are taken.

#### 2.2.4. Boundary Control

After a fisherman’s position is updated, it is determined whether its position is out of bounds, and the dimensions that are out of bounds are regenerated according to Equation (3).

## 3. The Newly Proposed MICFOA

As a newly proposed human-based meta-heuristic algorithm, CFOA has shown promising performance on clustering problems. However, by analyzing the searching method of CFOA, it is found that CFOA suffers from an imbalance between exploitation and exploration abilities, and the diversity of the population is weakened at the later stage. Specifically, CFOA exhibits too much randomness in the independent search phase, which is favorable for extensive search, but too blind a search is not conducive to the convergence of the algorithm. CFOA uses the position of the best fisherman as the reference point in the collective capture phase, which accelerates convergence but is easy to fall into the local optimum. Furthermore, CFOA gives no consideration to the replacement problem when the fishermen get tired; in other words, there is no strategy for solving the problem when the algorithm becomes stagnant. Therefore, to address the above problems, we adopt three strategies to improve CFOA. In this section, we provide a comprehensive description of the three strategies adopted to strengthen CFOA and present the detailed framework of the proposed MICFOA.

### 3.1. Lévy-Based Differential Independent Search Strategy

In the independent search phase, fishermen modify their fishing behavior based on their own position and that of one other random fisherman. The approach does not take into account the valid information of other better fishermen, making the CFOA highly exploratory in the early stages. However, the favorable performance of metaheuristic algorithms relies on the balance between exploitation performance and exploration performance. Therefore, too much global exploration capability at an early stage can weaken the performance of an algorithm. To solve this problem, inspired by the JADE algorithm, this paper proposes a Lévy-based differential independent search strategy (LDIS). The JADE algorithm uses the DE/current-to-pbest/1 mutation strategy for the mutation operation. The strategy does not only use the information of the best individual, but it also takes the information of other better individuals and considers the difference between these individuals as a direction to guide the evolution of the population. For the CFOA, the independent search phase is dominated by the global exploration capability with a certain exploitation capability. The LDIS will combine location information from itself, a random individual, and a randomly selected better individual to perform a location update. The specific method is shown below.

(13) $$X_{i,j}\left(t+1\right)=X_{i,j}\left(t\right)+RL\times XA+RL\times XB$$

(14) $$XA=\left(X_{r,j}\left(t\right)-X_{i,j}\left(t\right)\right)\times Exp+rand\times s\times R$$

(15) $$XB=\left(X_{pb,j}\left(t\right)-X_{i,j}\left(t\right)\right)\times Exp_{2}+rand_{2}\times s_{2}\times R_{2}$$

(16) $$N_{p}=N\times \left(p_{\max}+\left(p_{\max}-p_{\min}\right)\times S\right)$$

(17) $$Exp_{2}=\frac{fit_{i}-fit_{r2}}{fit_{\max}-fit_{\min}}$$

where $X_{pb,j}\left(t\right)$ denotes a random selection of fisher locations different from the top $N_{p}$ individuals of the current population. $p_{\max}$ and $p_{\min}$ are the greediness factors used to balance exploitation and exploration. *Exp*_2_ is the experience obtained by the *i*th fisherman $X_{i,j}\left(t\right)$ using $X_{pb,j}\left(t\right)$ as the reference person. $fit_{r2}$ denotes the fitness of a randomly selected individual. RL is a random number that follows a Lévy motion. The Lévy flight is characterized by frequent short-range searches and occasional long-range searches, so the random step size of the Lévy flight obeys the Lévy distribution, which has a stronger perturbation effect and can delay approaching a mere local optimum. The expression of the power form of the Lévy distribution is as follows.

(18) $$RL=\frac{\mu }{{\left|v\right|}^{1/\beta }}$$

(19) $$\begin{array}{c} \sigma_{\mu }={\left(\frac{\Gamma \left(1+\beta \right)\times \sin\left(\frac{\pi \times \beta }{2}\right)}{\Gamma \left(\frac{1+\beta }{2}\right)\times \beta \times 2^{\frac{\beta -1}{2}}}\right)}^{\frac{1}{\beta }} \end{array}$$

where RL is the step length of the Lévy flight, and β is a fixed constant set to 1.5. $\mu =N(0,{\sigma^{2}}_{\mu })$ and $v=N(0,{\mu_{v}}^{2})$ are both normal stochastic distributions. $\mu_{v}$ is equal to 1. Γ represents the standard gamma function.

### 3.2. Balancing Selection Mechanism

The CFOA changes its fishing position during the collective capture phase by referring to the best fisherman and the average position of all fishermen. There are two shortcomings involved in the process. One is that the position of the best fisherman determines the search direction of the remaining fishermen. When the best fisherman falls into a local optimum, it can lead to stagnation of the whole fisherman population. The second is that each fisher’s contribution to the population is different. Referring to the average position of the whole population will weaken the convergence ability of the algorithm. In order to make improvements to the deficiencies of CFOA in the exploitation phase, this paper proposes a balancing selection mechanism (BSM). While most meta-heuristic algorithms judge the goodness of an agent based on the fitness score, this paper proposes to evaluate the quality of an agent based on both fitness and distance. For the selection of the optimal fishermen, we chose to use a roulette strategy to select from all fishermen. This ensures a high selection rate for the best group, providing sufficient exploitation capacity, while having the probability of selecting the rest of the group, enhancing the ability of the algorithm to jump out of the local optimum. Moreover, we use a weighted average position instead of an arithmetic average position, which better represents the contribution of each fisherman to the population. It is worth noting that all the above improvements are based on the simultaneous consideration of fitness and distance as evaluation criteria. The specifics of the BSM are denoted as follows.

(20) $$Score\left(X_{i}\right)=\omega_{1}\times norm\left(fit_{i}\right)+\omega_{2}\times norm\left(dis_{i}\right)$$

where $Score\left(X_{i}\right)$ is the score of the *i*^th^ fisherman. $norm\left(\cdot \right)$ denotes normalized computation. $dis_{i}$ is the Euclidean distance between the *i*th fisher and the best fisher. $\omega_{1}$ and $\omega_{2}$ are the weights of the two metrics, both of which are 0.5 in this paper. After getting the score of each fisherman, a fisherman $X_{BSM}\left(t\right)$ will be selected to replace the optimal fisherman according to the roulette strategy. The weighted average position can be obtained using Equation (19).

(21) $$X_{wmean}\left(t\right)=\sum_{i=1}^{N}\gamma_{i}\times X_{i}\left(t\right)$$

(22) $$\gamma_{i}=\ln\left(N+1\right)/\left(\sum_{i=1}^{N}\left(\ln\frac{\left(N+1\right)}{i}\right)\right)$$

where $X_{wmean}\left(t\right)$ is the weighted average position. The modified collective capture strategy using BSM is represented as follows.

(23) $$X_{i}\left(t+1\right)=X_{BSM}\left(t\right)+GD\left(0,\frac{r_{2}\times \sigma \times \left|X_{wmean}\left(t\right)-X_{Gb}\right|}{3}\right)$$

### 3.3. Fishermen Position Replacement Strategy

**Figure 1:**
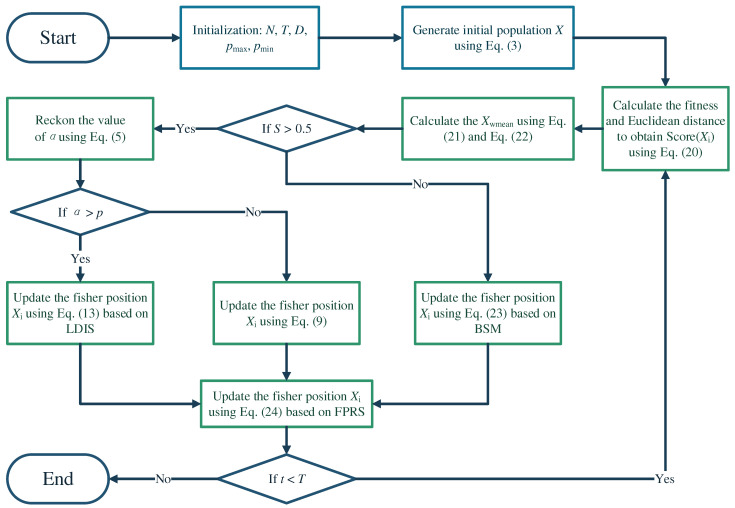
The flowchart of MICFOA.

Fishers will be less efficient when fishing due to reduced physical strength. At this point, consideration should be given to replacing fishermen with reduced physical strength by fishermen who are not engaged in the work. This situation is not considered in the basic CFOA. Therefore, we refine the structure of CFOA and propose a fishermen position replacement strategy (FPRS). The strategy cores are how to judge that the fishermen need to be replaced and the way to replace them. Metaheuristic algorithms are unfortunately often prone to local optima due to the lack of population diversity. Therefore, we judge that a fisherman needs to be replaced based on the fact that the fisherman is stuck in stagnation. In FPRS, we set a counter Ac for each fisherman. The initial value is 0. When the fisherman’s position after updating is worse than the position before updating, Ac is increased by 1; vice versa, the value of Ac remains unchanged. As the updating progresses, when Ac accumulates to a certain value, we consider that the fisherman’s physical strength has decreased seriously and needs to be replaced. The newly added fisherman can fully consider the effective information of the population, which then enhances population diversity and helps the populations jump out of stagnation. The equation of FPRS is expressed as follows.

(24) $$X_{i,j}\left(t+1\right)=\left\{\begin{array}{l} X_{i,j}\left(t\right)+{\left(1-S\right)}^{2S}\times \left(lb_{j}+rand\times {(\mathrm{ub}}_{j}-lb_{j})\right),r_{3}\leq rs\\ X_{i,j}\left(t\right)+\left(rs\times (1-r_{4})+r_{4}\right)\times \left(X_{ra,j}\left(t\right)-X_{rb,j}\left(t\right)\right),r_{3}>rs \end{array}\right.$$

where $r_{i}\in (0,1),i=3,4$ is a random number. rs is a constant value equal to 0.2. $X_{ra,j}\left(t\right)$ and $X_{rb,j}\left(t\right)$ are two randomly selected fishermen. Applying the above improvement strategies to the basic CFOA, we obtain the proposed MICFOA algorithm. The pseudo-code of MICFOA is shown in Algorithm 1, and the flowchart is shown in Figure 1.
**Algorithm 1:** MICFOA
1.Initial parameters: N, T, t, $p_{\max}$, $p_{\min}$2.Generate initial population X using Equation (3)3.While t < T4. Calculate the fitness and Euclidean distance to obtain $Score\left(X_{i}\right)$ using Equation (20)5. Calculate $X_{wmean}$ using Equations (21) and (22)6. For each fisher $X_{i}$ do7.  If S < 0.58.   Reckon the value of α using Equation (5)9.    If α>p10.     Update the fisher position $X_{i}$ using Equation (13) based on LDIS11.    Else12.     Update the fisher position $X_{i}$ using Equation (9)13.    End if14.  Else15.   Update the fisher position $X_{i}$ using Equation (23) based on BSM16.   End if17. Update the fisher position $X_{i}$ using Equation (24) based on FPRS18. End for19.End while20.Output best fisher position $X_{Gb}$

### 3.4. Computational Complexity Analysis

The population size of fishers is *N*, the search space dimension is *D*, and the maximum number of iterations is *T*. Since each fisher executes only one search strategy, the time complexity of CFOA is $O\left(T\times N\times D\right)$. In the proposed MICFOA, the LDIS and BSM strategies do not create additional loops and therefore do not increase the time complexity. As for the FPRS, we assume that *N*_f_ fishermen are replaced in each iteration, and the time complexity of the strategy is $O\left(T\times N_{f}\times D\right)$. As a result, the time complexity of MICFOA is $O\left(T\times \left(N_{f}+N\right)\times D\right)$.

## 4. Numerical Experiment and Results Analysis

In this section, the performance of the proposed MICFOA is comprehensively evaluated based on the CEC 2017 test set and the CEC 2022 test set. The detailed information on the CEC 2017 test set and the CEC 2022 test set can be found in the literature [45,46].

### 4.1. Experimental Environments and Parameter Settings

All experiments are conducted on a Windows 10 computer with an AMD R7 4800U 4.20 GHz processor and 16.0 GB of RAM. All algorithms are implemented using MATLAB 2020b software.

In the experiments, the dimensions of functions in the CEC 2017 test set are 10, 30, 50, and 100, and the dimensions of functions in the CEC 2022 test set are 10 and 20. The search ranges are both [−100, 100] D. All the test functions are solved independently by the participating algorithms 30 times. The obtained best value, mean, and standard deviation are recorded. In this work, the population *N* is set to 30, and the maximum number of iterations *T* is set to 500.

In order to analyze the results obtained by MICFOA and the comparison algorithms, two statistical tests, including the Wilcoxon rank sum test and the Friedman test, are applied to statistically analyze the superiority or inferiority of MICFOA and the competitors. The Wilcoxon rank sum test can judge how well or poorly MICFOA and the other algorithms are performing on each function. The Friedman test can provide the ranking of all the algorithms and determine whether there is any difference between MICFOA and the competing algorithms. Violin plots and convergence plots are employed to analyze the robustness and convergence of MICFOA and the competitors.

In order to fully evaluate the performance of MICFOA, comparison experiments with metaheuristic algorithms of four different classes totaling eleven were conducted.

(1) Human-based algorithms: CFOA, PEOA [47], and TLBO [37];
(2) Swarm-based algorithms: COA [8] and ARO [48];
(3) Physics-based algorithms: EDO [49] and YDSE [50];
(4) Evolution-based algorithms: LSHADE [12], JADE [11], IDE-EDA [13], and APSM-jSO [14].

These comparison algorithms contain classical algorithms as well as recently proposed new algorithms and variants of excellent evolution-based algorithms. In particular, LSHADE is the winner of the IEEE 2014 Conference on Evolutionary Computation. IDE-EDA is an algorithm with performance similar to that of the LSHADE-RSP algorithm, the winner of the IEEE 2018 Conference on Evolutionary Computation. APSM-jSO is an improved version of the runner-up of the IEEE 2018 Conference on Evolutionary Computation. The comparison with these algorithms enables a comprehensive assessment and illustration of the superiority of MICFOA. For a fair comparison, the parameters of those competitors are set to the recommended values by the authors of each paper, as shown in Table 1.

### 4.2. Strategy Effectiveness Analysis

In this subsection, the effect of the three strategies on MICFOA is examined through strategy effectiveness analysis experiments. For illustration purposes, three MICFOA-derived algorithms, namely MICFOA-1, MICFOA-2, and MICFOA-3, were designed for experimental purposes. Detailed information on MICFOA and its derived algorithms is shown in Table 2. These algorithms will be compared on the CEC2017 test set and the CEC2022 test set, and the results obtained will be analyzed using the Wilcoxon rank sum test and the Friedman test. Table 3 presents the results of the Friedman test for MICFOA and the derived algorithms at the significance level a = 0.05. The rankings are obtained by ranking all the algorithms according to their mean values in solving the test function, and the *p*-values reflect whether or not these algorithms have differences.

**Figure 2:**
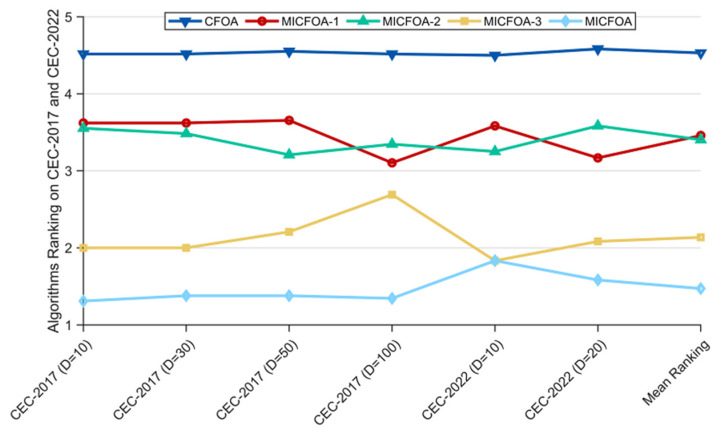
Ranking of MICFOA and three derived algorithms based on the Friedman test.

Based on the *p*-values in the last row of Table 3, we can observe that the *p*-values for all dimensions are not greater than 0.05, which indicates that there is a significant difference between the five algorithms in this subsection in terms of performance. Figure 2 exhibits the Friedman rankings of MICFOA and the derived algorithms. Specifically, all three MICFOA-derived algorithms combining a single strategy rank better than the basic CFOA, which indicates that all strategies can enhance the performance of MICFOA. MICFOA achieves the best ranking in all cases, which means that MICFOA outperforms both the three derived algorithms and the basic CFOA regarding overall performance, and it also indicates that the three improved strategies do not have a negative effect on each other. MICFOA-3 ranks first in 10D of CEC 2022 alongside MICFOA, and is only inferior to MICFOA in other scenarios, which suggests that the FPRS positively affects the performance of MICFOA. In summary, the effectiveness of the three improvement strategies on MICFOA is from most to least: FPRS > BSM > LIDS.

**Figure 3:**
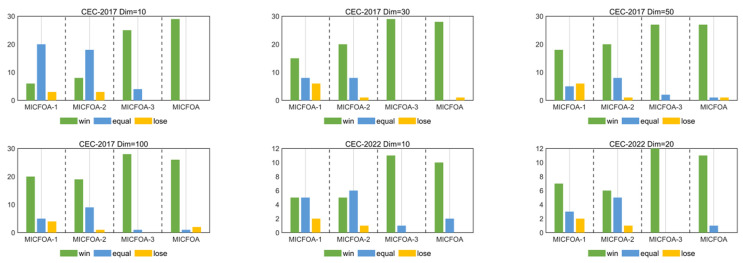
Win/equal/loss statistics of MICFOA and derived algorithms compared to CFOA.

The Wilcoxon rank sum test can provide information about the differences between MICFOA and the derived algorithms compared to the basic CFOA. Table 4 records the statistical results. In Table 4, “+” indicates the total number of functions for which MICFOA and derived algorithms outperform the basic CFOA. “=” indicates that their performances are statistically similar. “−” indicates the total number of functions for which CFOA is better. Figure 3 visualizes the statistical results of Table 4. It can be seen that the number of “+” for MICFOA and derived algorithms is more than the number of “−”, which implies that MICFOA and derived algorithms significantly outperform the basic CFOA. In a word, the Wilcoxon rank-sum test confirms again the superiority of the MICFOA algorithm proposed in this paper.

### 4.3. Comparison with Other Competitors Using the CEC 2017 Test Set

**Figure 4:**
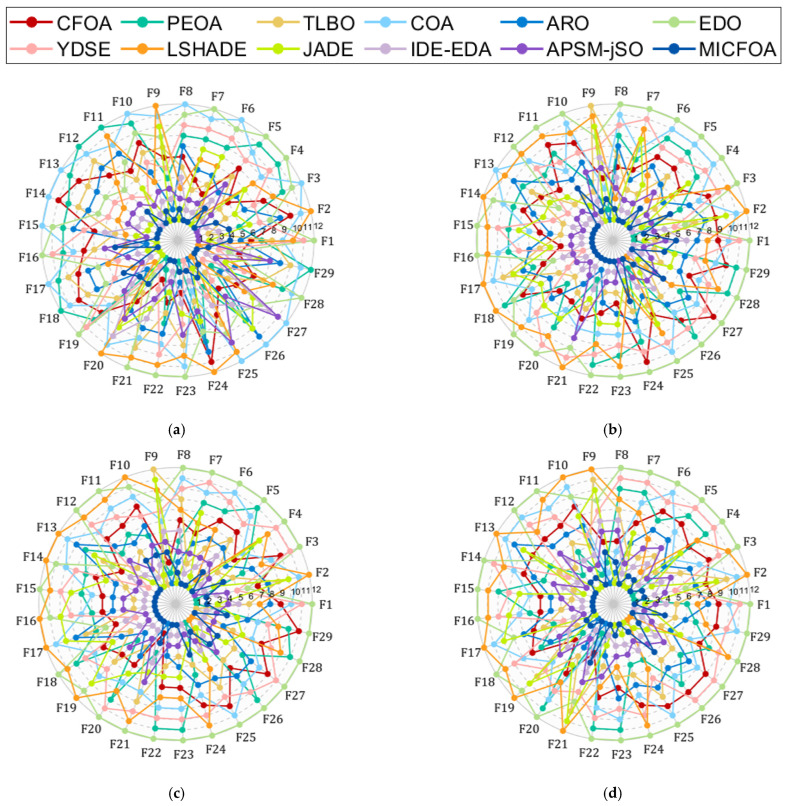
Rankings based on “Mean” of MICFOA and competitors on the CEC2017 test set. (**a**) D = 10; (**b**) D = 30; (**c**) D = 50; (**d**) D = 100.

In this section, the CEC 2017 test set was utilized to evaluate the search capability of MICFOA. The detailed results obtained by MICFOA, CFOA, PEOA, TLBO, COA, ARO, EDO, YDSE, LSHADE, JADE, IDE-EDA, and APSM-jSO in the CEC 2017 test set (D = 10, 30, 50, 100) are presented in Table A1, Table A2, Table A3 and Table A4 in Appendix A, where “ Best”, ‘Mean’ and ‘Std’ denote the optimal value, the average value, and the standard deviation of the results. Prior to statistical analysis, spider plots were chosen to visualize the mean-based rankings of MICFOA and competitors on each function, as shown in Figure 4. The area enclosed by each curve of the spider diagram represents the performance of the corresponding algorithm. We can roughly conclude from Figure 4 that MICFOA outperforms the competition in all six conditions.

#### 4.3.1. Analysis of the Wilcoxon Rank Sum Test Results

**Figure 5:**
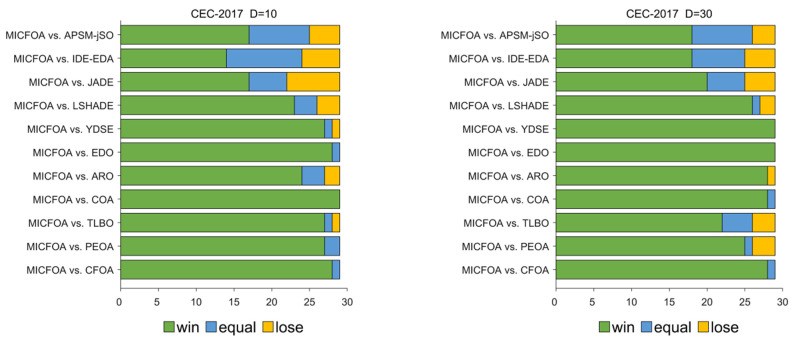

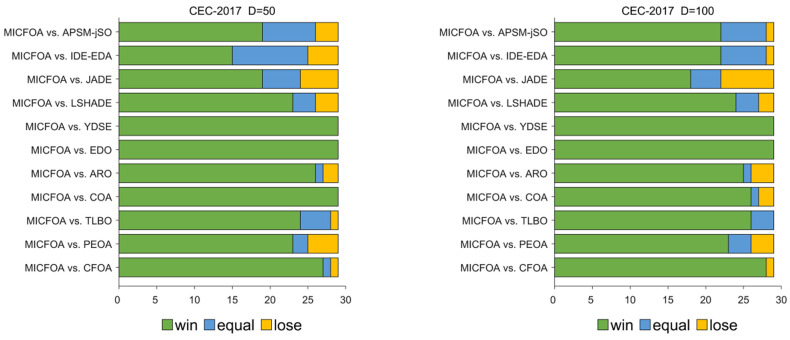
The Wilcoxon rank sum test results from the perspective of the test function dimension.

Table 5 summarizes the counts of how MICFOA is superior, similar, or inferior to competitors in the four dimensions of the CEC2017 test set, based on the results of the Wilcoxon rank-sum test. The “Total” in Table 5 shows that MICFOA is superior (inferior) to CFOA, PEOA, TLBO, COA, ARO, EDO, YDSE, LSHADE, JADE, IDE-EDA, and APSM-jSO on 111(2), 98(10), 99(5), 112(2), 103(8), 115(0), 114(1), 96(10), 74(23), 69(14), and 76(11) test functions, i.e., when comparing MICFOA to these competitors, the number of “+” totals more than “−”. This means that MICFOA outperforms the 11 comparison algorithms in overall performance. Figure 5 intuitively shows the statistical results of the Wilcoxon rank sum test to evaluate the algorithm performance from the perspective of different dimensions of functions. The details of the analysis are as follows.

For D = 10, MICFOA is superior (inferior) to CFOA, PEOA, TLBO, COA, ARO, EDO, YDSE, LSHADE, JADE, IDE-EDA, and APSM-jSO on 28(1), 27(0), 27(1), 29(0), 24(2), 28(0), 27(1), 23(3), 17(7), 15(5), and 17(4) functions. In other words, MICFOA beats the competitors on the 10D functions.

For D = 30, MICFOA is superior (inferior) to CFOA, PEOA, TLBO, COA, ARO, EDO, YDSE, LSHADE, JADE, IDE-EDA, and APSM-jSO on 28(1), 25(3), 22(3), 28(0), 28(1), 29(0), 29(0), 23(3), 19(5), 18(4), and 18(3) functions. In other words, MICFOA beats the competitors on the 30D functions.

For D = 50, MICFOA is superior (inferior to) to CFOA, PEOA, TLBO, COA, ARO, EDO, YDSE, LSHADE, JADE, IDE-EDA, and APSM-jSO on 27(1), 23(4), 24(1), 29(0), 26(2), 26(2), 27(1), 23(3), 17(7), 15(4), and 19(3) functions. In other words, MICFOA beats the competitors on the 50D functions.

For D = 100, MICFOA is superior (inferior to) to CFOA, PEOA, TLBO, COA, ARO, EDO, YDSE, LSHADE, JADE, IDE-EDA, and APSM-jSO on 28(1), 23(3), 26(0), 26(2), 25(3), 29(0), 29(0), 24(2), 18(7), 22(1), and 22(1) functions. In other words, MICFOA beats the competitors on the 100D functions.

#### 4.3.2. Analysis of the Friedman Test Results

**Figure 6:**
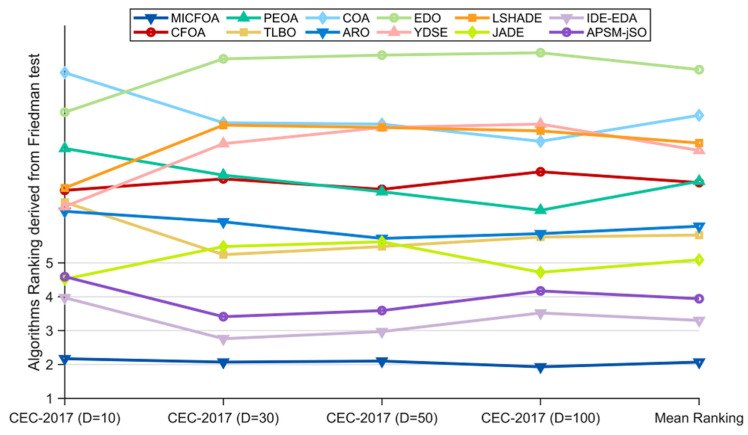
Ranking of MICFOA and competitors based on the Friedman test.

In this subsection, the overall performance of MICFOA and the competitors is analyzed, based on the results of the Friedman test. Table 6 summarizes the Friedman scores and other statistical metrics for all algorithms. In order to visualize the Friedman test results more intuitively, Figure 6 visualizes these scores. As can be seen in Table 6, the *p*-values of 10D, 30D, 50D, and 100D are all less than 0.05, which indicates that there is a significant difference between the performance of MICFOA and the competitors in all dimensions of the CEC2017 test set. The results of the Friedman test are specified below:

(1) For 10D, MICFOA ranks in first place followed by IDE-EDA, JADE, APSM-jSO, ARO, YDSE, TLBO, CFOA, LSHADE, PEOA, EDO, and COA. That is, MICFOA outperforms all competitors on 10D functions.
(2) For 30D, MICFOA ranks in first place followed by IDE-EDA, APSM-jSO, TLBO, ARO, JADE, CFOA, PEOA, YDSE, LSHADE, COA and EDO. That is, MICFOA outperforms all competitors on 30D functions.
(3) For 50D, MICFOA ranks in first place followed by IDE-EDA, APSM-jSO, TLBO, JADE, ARO, PEOA, CFOA, LSHADE/YDSE, COA and EDO. That is, MICFOA outperforms all competitors on 50D functions.
(4) For 100D, MICFOA ranks in first place followed by IDE-EDA, APSM-jSO, JADE, TLBO, ARO, PEOA, CFOA, COA, LSHADE, YDSE and EDO. That is, MICFOA outperforms all competitors on 100D functions.

Based on these specific results, MICFOA outperforms CFOA, PEOA, TLBO, COA, ARO, EDO, YDSE, LSHADE, JADE, IDE-EDA, and APSM-jSO in all dimensions of the CEC 2017 test suite. In addition, in terms of “Mean Ranking”, MICFOA achieves first place, followed by IDE-EDA, APSM-jSO, and JADE. Therefore, the Friedman test results support the conclusion that MICFOA significantly outperforms all competitors in the CEC 2017 test suite, which indicates that MICFOA notably improves the performance of CFOA.

**Figure 7:**
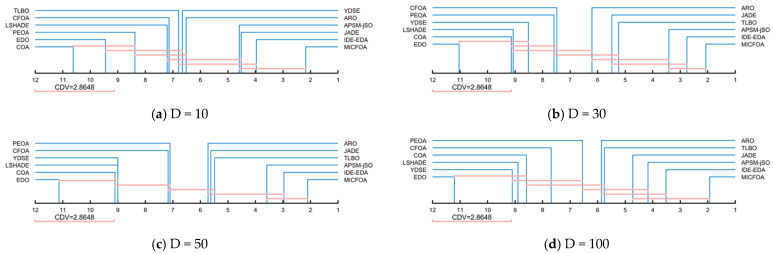
Multiple comparisons between MICFOA and competitors based on the Nemenyi test.

The Friedman test provides a score for each algorithm. On this basis, the Nemenyi test is applied to further evaluate the magnitude of the difference between MICFOA and the other algorithms. Figure 7 shows the visualization results using the Nemenyi test. There is no significant difference between the algorithms connected with blue line segments in Figure 7. According to Figure 7, there is no significant difference between MICFOA, IDE-EDA, APSM-jSO, and JADE in 10D and 100D. For 30D and 50D, there is no significant difference between MICFOA, IDE-EDA, APSM-jSO, and TLBO. For all other algorithms, MICFOA shows significant superiority.

#### 4.3.3. Analysis of Convergence and Robustness

**Figure A1:**
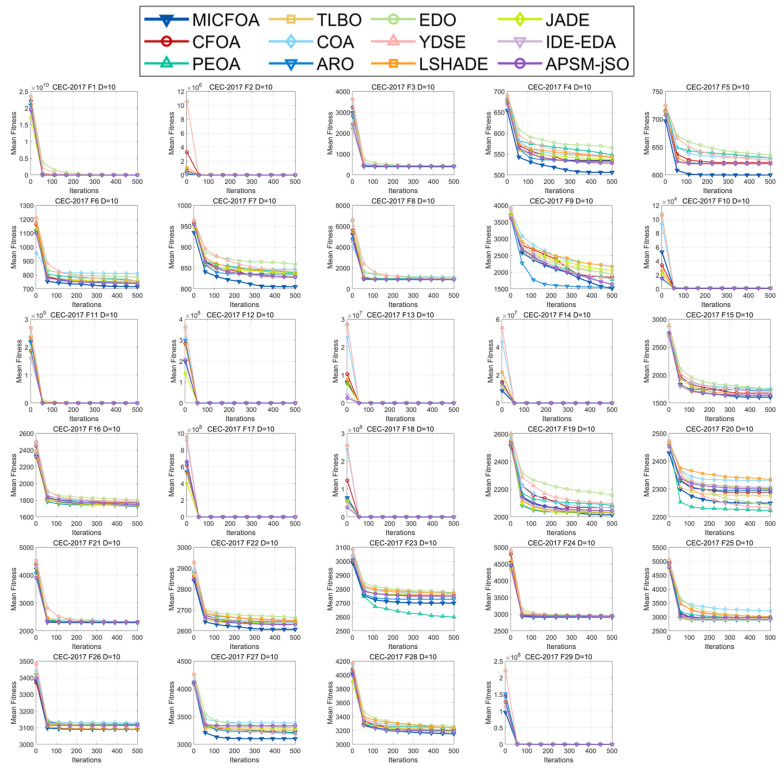
The convergence curves of MICFOA and competitors on CEC 2017 (D = 10).

**Figure A2:**
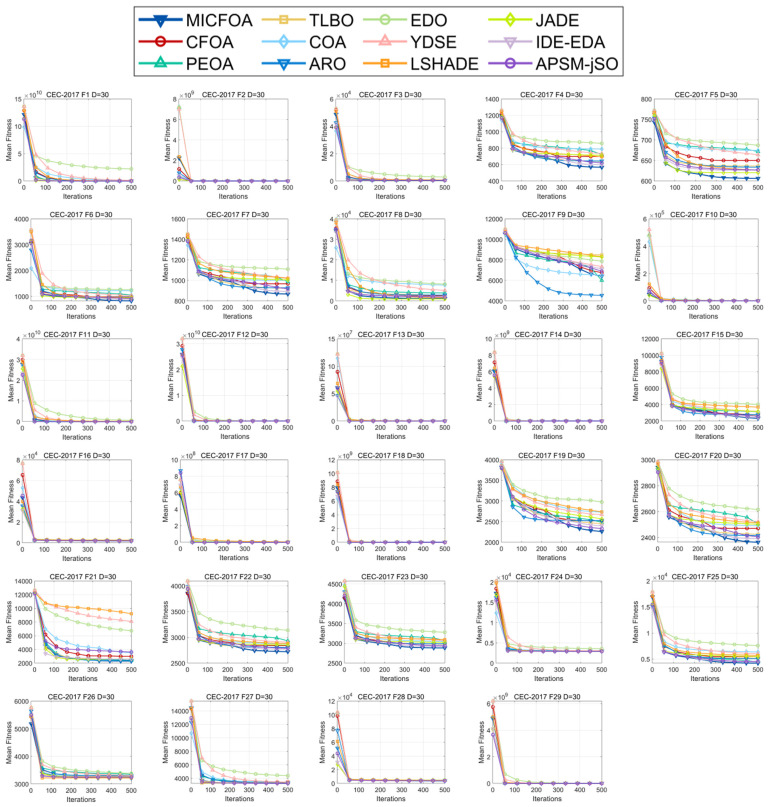
The convergence curves of MICFOA and competitors on CEC 2017 (D = 30).

**Figure A3:**
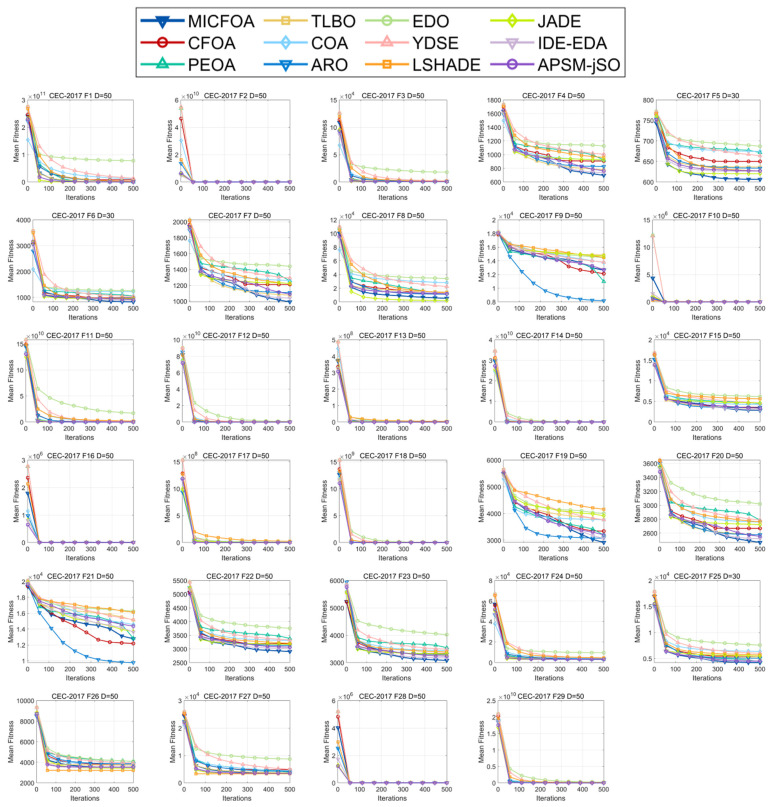
The convergence curves of MICFOA and competitors on CEC 2017 (D = 50).

**Figure A4:**
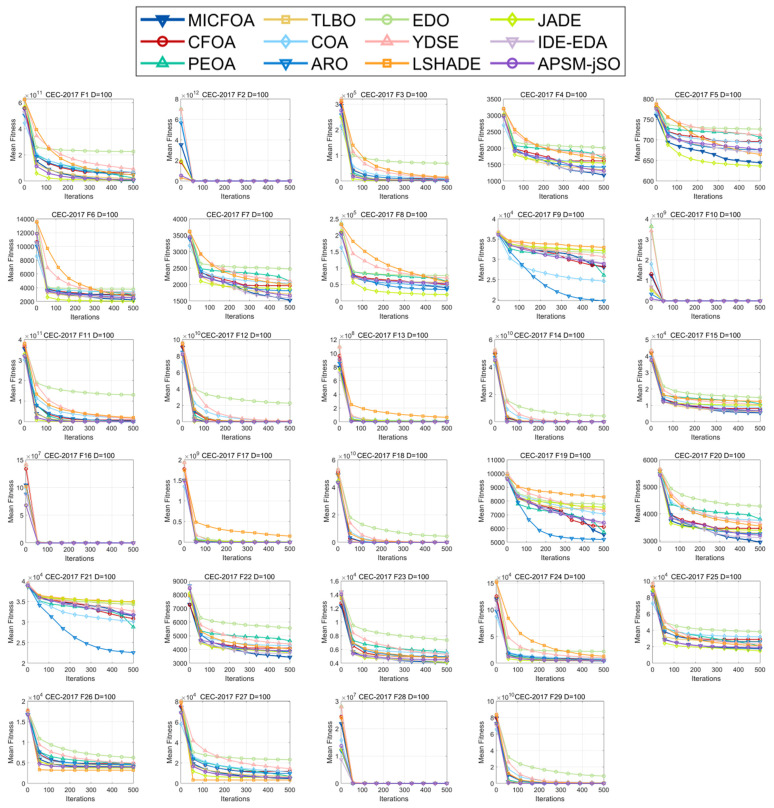
The convergence curves of MICFOA and competitors on CEC 2017 (D = 100).

**Figure A5:**
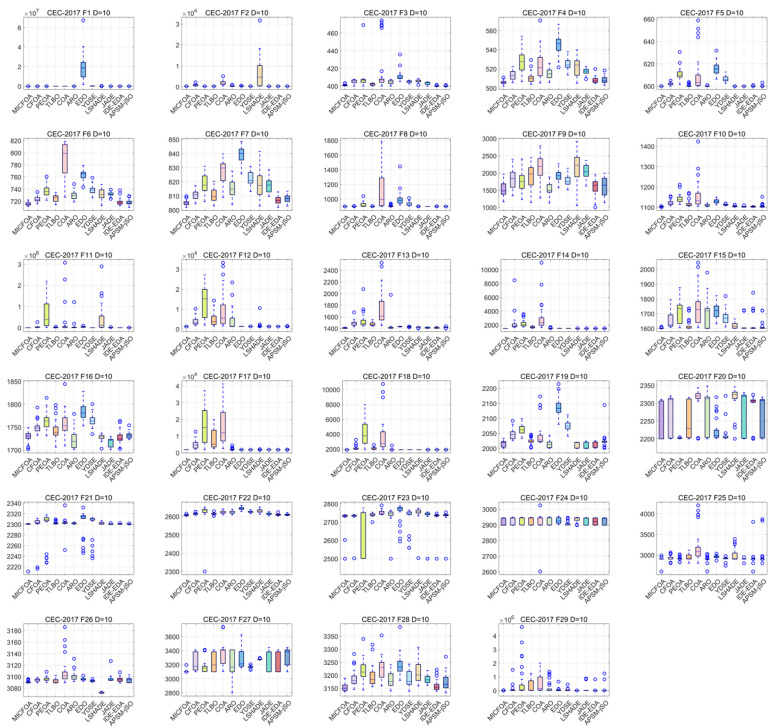
The box plots of MICFOA and competitors on CEC 2017 (D = 10).

**Figure A6:**
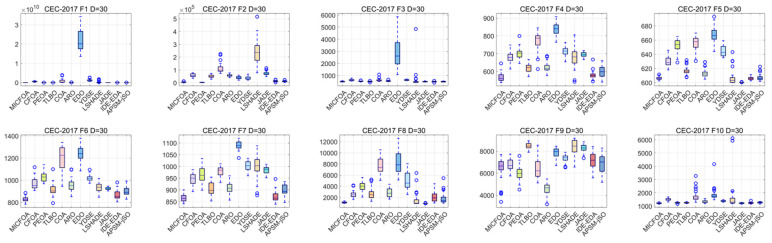

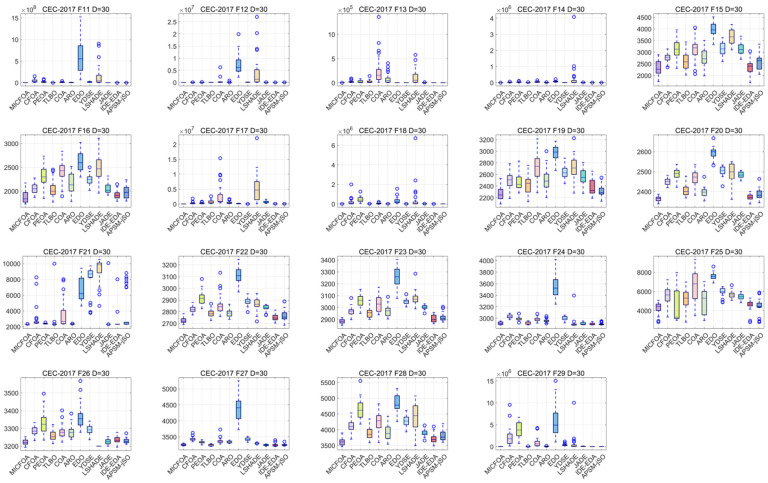
The box plots of MICFOA and competitors on CEC 2017 (D = 30).

**Figure A7:**
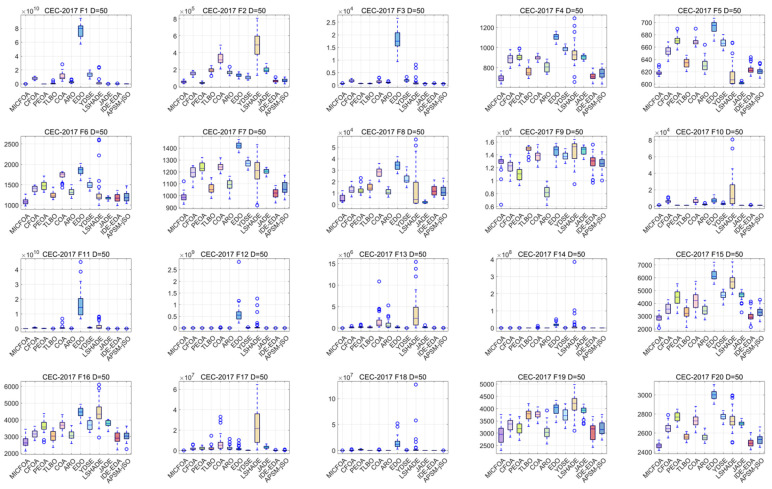

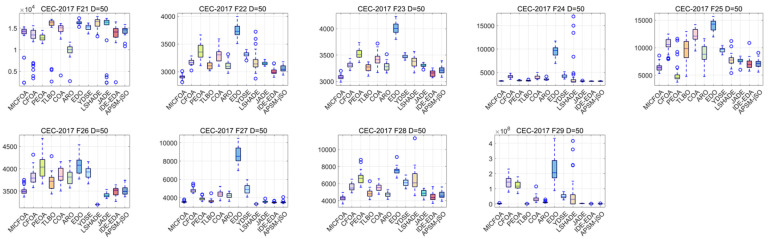
The box plots of MICFOA and competitors on CEC 2017 (D = 50).

**Figure A8:**
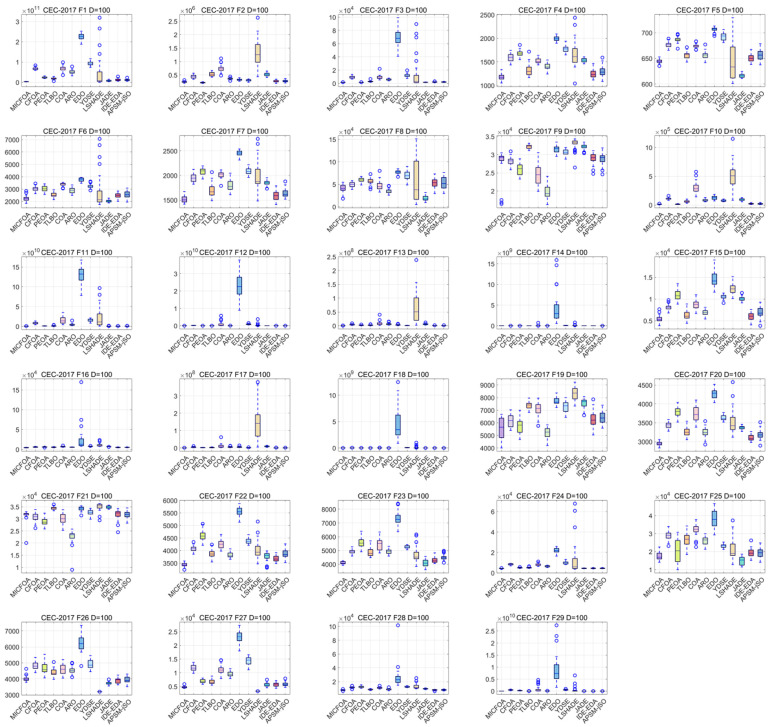
The box plots of MICFOA and competitors on CEC 2017 (D = 100).

In this subsection, the convergence and stability of MICFOA on the CEC2017 test set are analyzed. Due to the fact that the CEC2022 test set is low dimensional, this part displays the convergence plots and box plots for the six CEC2017 test functions on 100D. Convergence curves and box plots for all CEC2017 test functions are available in Figure A1, Figure A2, Figure A3, Figure A4, Figure A5, Figure A6, Figure A7 and Figure A8 from Appendix A.

**Figure 8:**
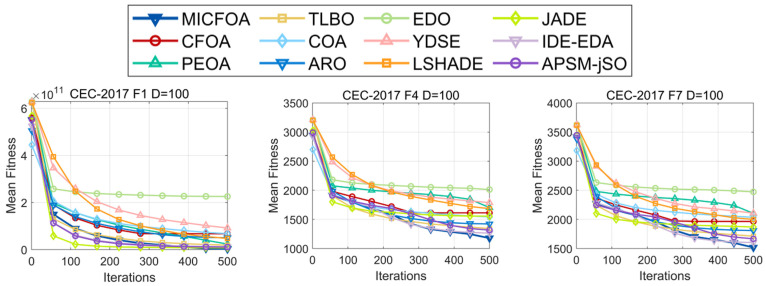

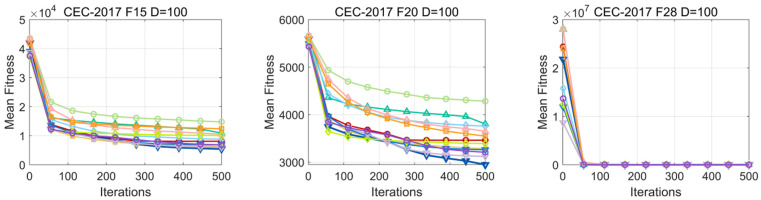
Convergence curves of MICFOA and competitors based on CEC2017 100D.

Figure 8 exhibits the average fitness convergence curves for MICFOA and the competitors based on the results of the six CEC2017 100D test functions. In terms of convergence speed, JADE can converge quickly, but the accuracy is inferior to that of MICFOA and IDE-EDA. MICFOA can converge to a higher precision in the late stage than any of the other competitors and exhibits the fastest late-stage convergence speed.

**Figure 9:**
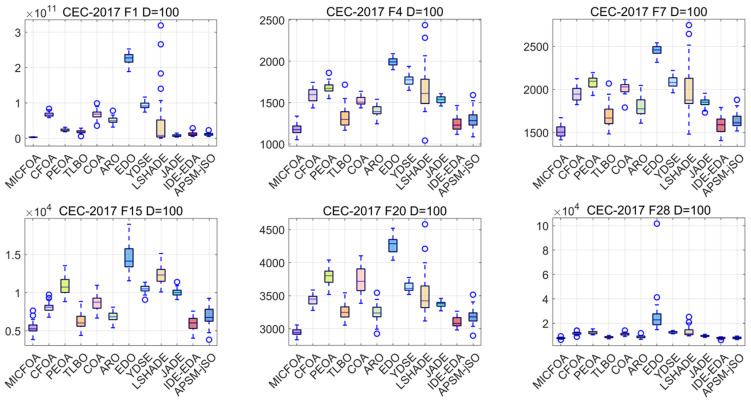
Box plots of MICFOA and competitors based on CEC2017 100D.

Box plots can be employed to assess the robustness of an algorithm. The width and height of a box reflect the quality of an algorithm. The symbol ‘o’ denotes an anomalous solution that deviates from the set of central solutions among the 30 results. The six CEC2017 100D test function results based on MICFOA and competitors solved independently 30 times are depicted in Figure 9. As observed in Figure 9, MICFOA has no outliers on F1, F4, F7, and F20 and has the narrowest and shortest box, and a few outliers on F15 and F28, but also has the narrowest and shortest box. The implication is that MICFOA exhibits the best robustness while having high performance.

### 4.4. Comparison with Other Competitors Using the CEC 2022 Test Set

**Figure 10:**
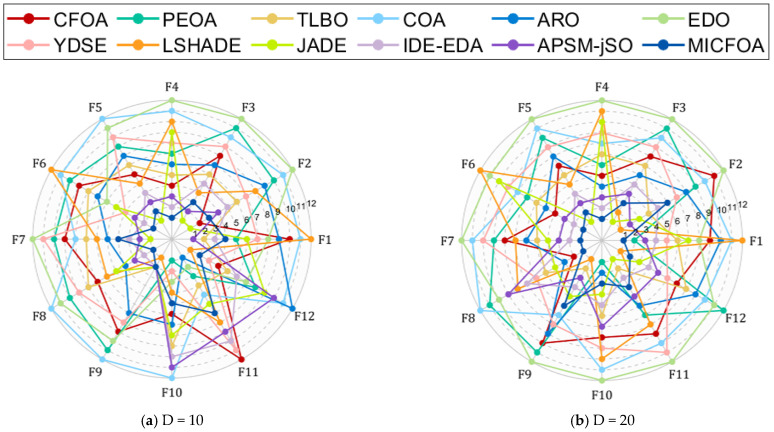
Rankings based on “Mean” of MICFOA and competitors on the CEC2022 test set.

To evaluate the performance of MICFOA more extensively, the CEC 2022 test set is implemented to further examine the search capabilities of MICFOA. The comparison algorithms are selected in the same way as in Section 4.3. The results of these algorithms on the CEC 2022 test set (D = 10, 20) are recorded in Table A5 and Table A6 within Appendix A. Similarly, the spider plots show the average rankings of MICFOA and its competitors on each function, as shown in Figure 10. We can roughly conclude from Figure 10 that MICFOA outperforms its competitors on both dimensions.

#### 4.4.1. Analysis of the Wilcoxon Rank Sum Test Results

**Figure 11:**
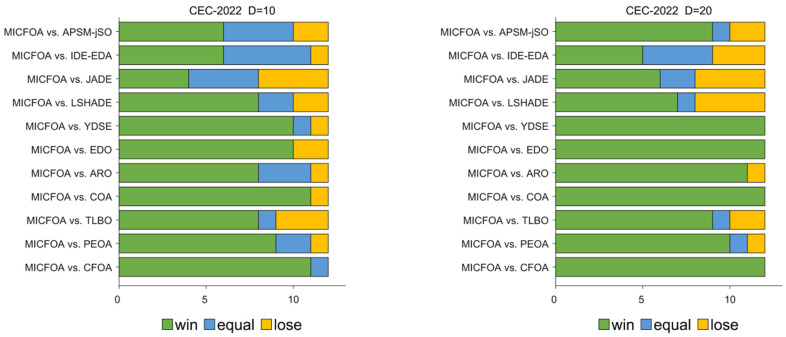
The Wilcoxon rank sum test results from the perspective of the test function dimension.

The results of the Wilcoxon rank sum test for MICFOA and the competitors on the CEC 2022 test set are recorded in Table 7. The quantities by which MICFOA outperforms, performs similarly to, and underperforms the competitors are visualized in Figure 11. Specific analyses are presented below.

For D = 10, MICFOA is superior (inferior) to CFOA, PEOA, TLBO, COA, ARO, EDO, YDSE, LSHADE, JADE, IDE-EDA, and APSM-jSO on 11(0), 9(1), 8(3), 11(1), 8(1), 10(2), 10(1), 8(2), 4(4), 6(1), and 6(2). In other words, MICFOA outperforms the competitors on 10D.

For D = 20, MICFOA is superior (inferior) to CFOA, PEOA, TLBO, COA, ARO, EDO, YDSE, LSHADE, JADE, IDE-EDA, and APSM-jSO on 12(0), 10(1), 9(2), 12(0), 11(1), 12(0), 12(0), 7(4), 6(4), 5(3), and 9(2). In other words, MICFOA outperforms the competitors on 20D.

According to the data shown in the last column of ‘Total’ in Table 7, MICFOA obtains more ‘+’ than ‘−’. This indicates that MICFOA outperforms the other participants in the CEC 2022 test set and has the best overall performance.

#### 4.4.2. Analysis of the Friedman Test Results

**Figure 12:**
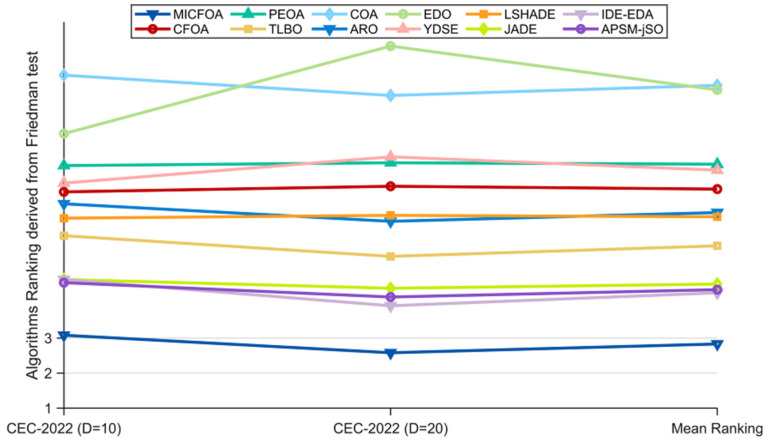
Ranking of MICFOA and competitors based on the Friedman test.

The results of the Friedman test between MICFOA and the participants on the CEC2022 test set are shown in Table 8 and visualized in Figure 12. The *p*-value in Table 8 is not greater than 0.05, which indicates that there is a significant difference between MICFOA and the participants. ‘Mean Ranking’ shows that MICFOA is ranked first with a mean Friedman score of 2.83, followed by IDE-EDA and APSM-jSO. The detailed analysis is as follows.

(1) For 10D, MICFOA ranks first, followed by APSM-jSO, IDE-EDA/JADE, TLBO, LSHADE, ARO, CFOA, YDSE, PEOA, EDO, and COA. That is, MICFOA outperforms all competitors on 10D.
(2) For 20D, MICFOA ranks first, followed by IDE-EDA, APSM-jSO, JADE, TLBO, ARO, LSHADE, CFOA, PEOA, YDSE, COA, and EDO. That is, MICFOA outperforms all competitors on 20D.

Based on these specific results, MICFOA outperforms CFOA, PEOA, TLBO, COA, ARO, EDO, YDSE, LSHADE, JADE, IDE-EDA, and APSM-jSO in all dimensions of the CEC 2022 test suite. Therefore, the Friedman test results support the conclusion that MICFOA significantly outperforms all competitors in the CEC 2022 test suite, which indicates that MICFOA notably improves the performance of CFOA.

#### 4.4.3. Analysis of Convergence and Robustness

**Figure 13:**
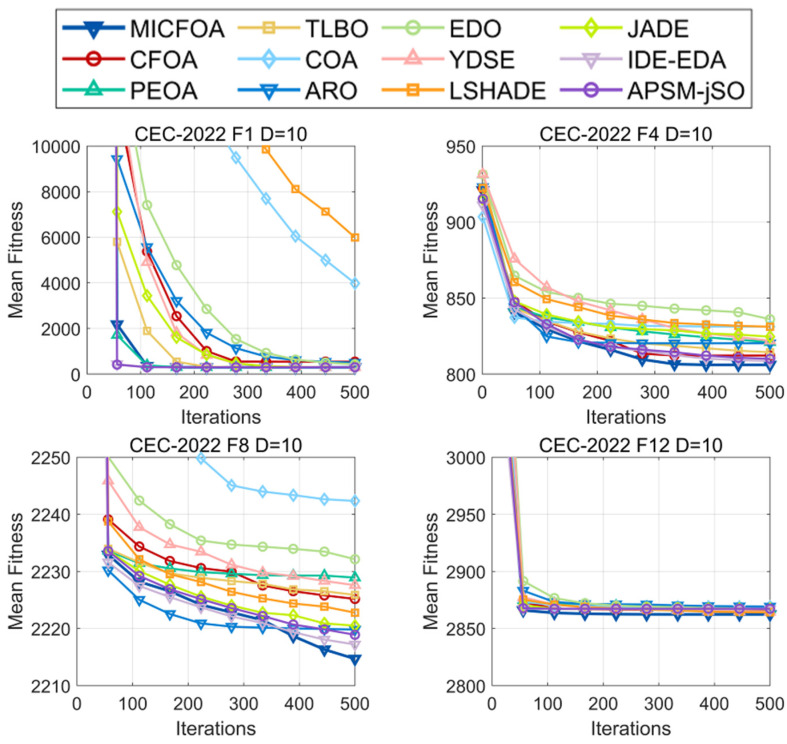
Convergence curves of MICFOA and competitors based on CEC2022 10D.

**Figure 14:**
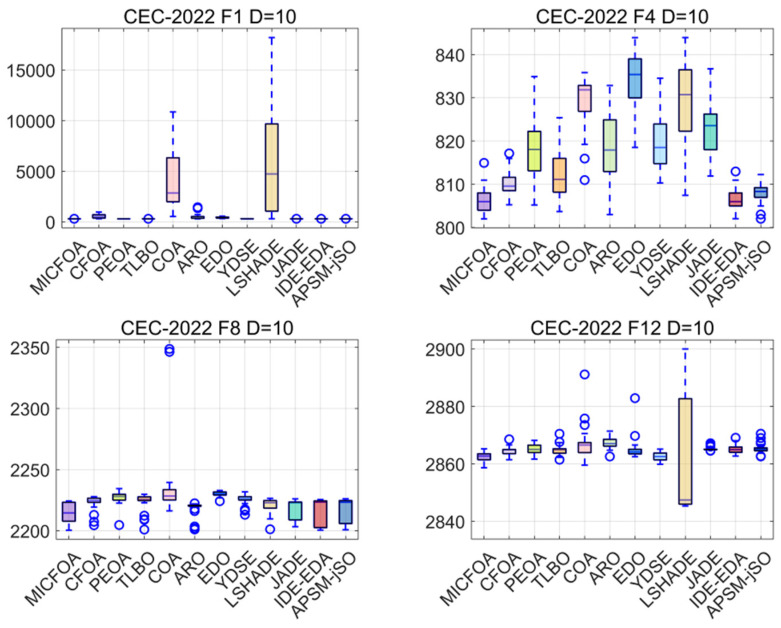
Box plots of MICFOA and competitors based on CEC2022 10D.

**Figure A9:**
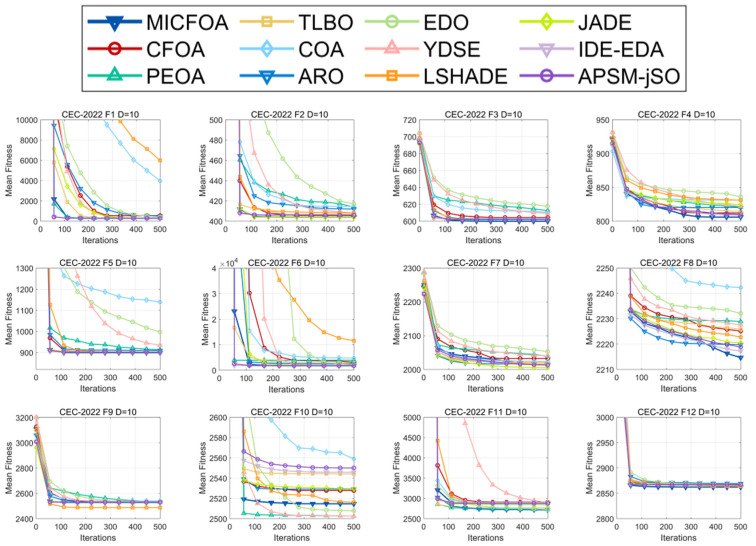
The convergence curves of MICFOA and competitors on CEC 2022 (D = 10).

**Figure A10:**
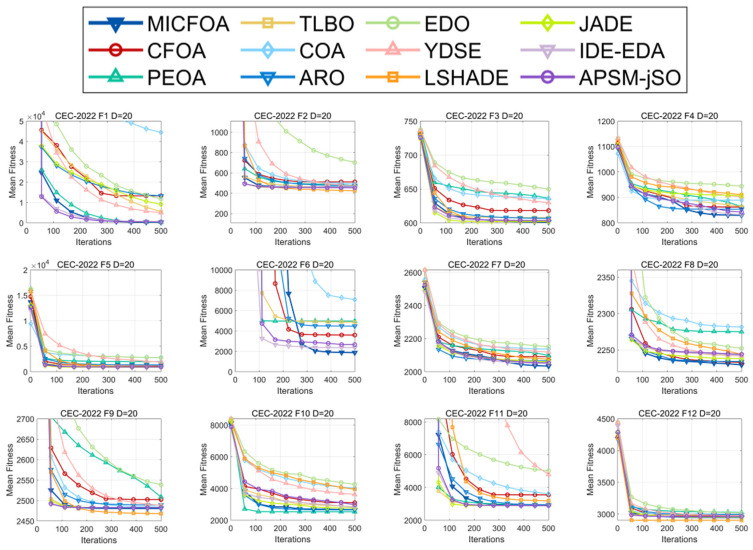
The convergence curves of MICFOA and competitors on CEC 2022 (D = 20).

**Figure A11:**
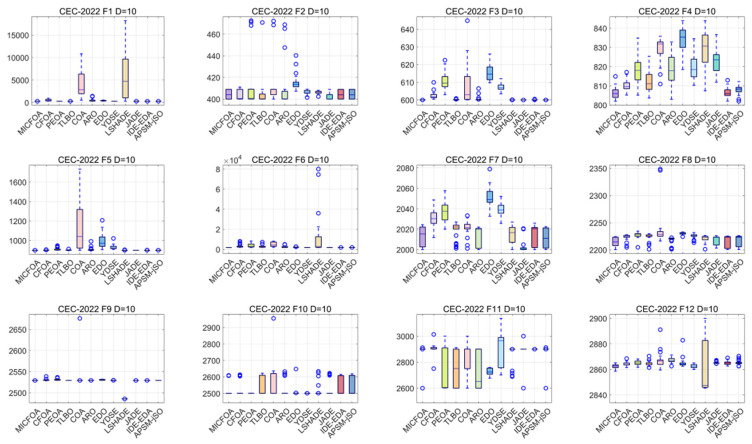
The box plots of MICFOA and competitors on CEC 2022 (D = 10).

**Figure A12:**
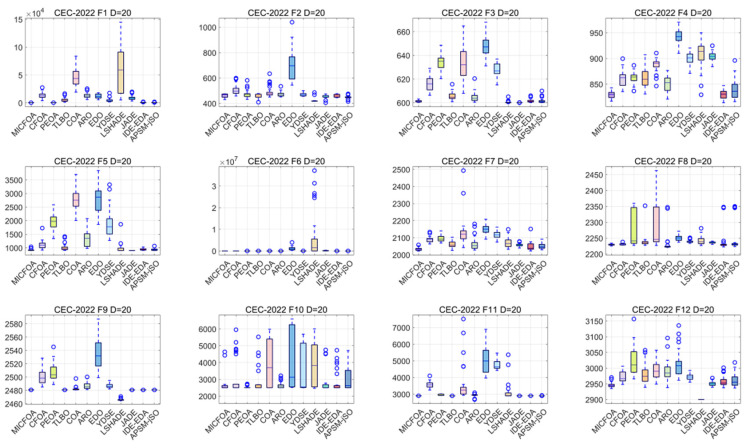
The box plots of MICFOA and competitors on CEC 2022 (D = 20).

With the performance of MICFOA on high-dimensional functions discussed in Section 4.3.3, this subsection further analyzes the performance of MICFOA on low-dimensional functions. Figure 13 and Figure 14 show the convergence curves and box plots for the four CEC2022 10D test functions. The entire convergence curves and box plots can be found in Figure A9, Figure A10, Figure A11 and Figure A12 from Appendix A.

In terms of convergence speed, MICFOA fails to demonstrate faster convergence than IDE-EDA and APSM-jSO. However, MICFOA has better convergence accuracy and exhibits the fastest late-stage convergence speed.

From the box plots in Figure 14, we can derive that MICFOA has fewer anomalies than its two excellent competitors, IDE-EDA and APSM-jSO. Although MICFOA does not have the narrowest box, it is the lowest overall. That is, the other competitors are stable but not very accurate, and MICFOA does not show the best robustness, but the overall results are excellent.

## 5. Conclusions

In this work, a novel CFOA variant called MICFOA is proposed to improve the performance of CFOA by introducing multiple improvement strategies incorporating a Lévy-based differential independent search strategy, weight-balanced selection mechanism, and fishermen position replacement strategy. In order to evaluate the effectiveness of the improvement strategies and the superiority of the proposed MICFOA, a comprehensive validation was performed on the CEC2017 test set and the CEC2022 test set. The analysis of strategy effectiveness shows that the three strategies designed to enhance CFOA can significantly improve the performance of CFOA. In order to fully reflect the performance of MICFOA, six basic algorithms are selected in this paper, including two human-based algorithms, PEOA and TLBO; two physics-based algorithms, EDO and YDSE; and two swarm-based algorithms, COA and ARO. Furthermore, four state-of-the-art evolution-based algorithms, LSHADE, JADE, IDE-EDA, and APSM-jSO, are also employed for comparison. The analysis of statistics, convergence and robustness confirms that MICFOA outperforms CFOA, POEA, TLBO, COA, ARO, EDO, YDSE, LSHADE, JADE, IDE-EDA, and APSM-jSO. In conclusion, the MICFOA proposed in this paper is a promising CFOA variant that can effectively promote the performance of CFOA.

The above experiments and analyses have validated the superiority of our algorithm. However, there is still some work to be conducted to continue the implementation. The parameters of CFOA can be further discussed to enhance its performance. Multi-objective and binary versions of MICFOA can be implemented for solving multi-objective optimization tasks and feature selection missions. The structure of MICFOA can be further refined so that it can be applied to solve real-time optimization problems with high timeliness requirements.

## Figures and Tables

**Table 1:** The Parameter settings for MICFOA and ten competitors.

Algorithm	Name of the Parameter	Value of the Parameter
MICFOA	c, p_max_, p_min_, rs, β	[3,4], 0.8, 0.4, 0.2, 1.5
CFOA	c	[3,4]
PEOA	Re, I	0.5, [1,2]
TLBO	TF	[1,2]
COA	C_1,_ C_3_, µ, σ	0.2, 3, 25, 3
ARO	k	1
EDO	switch	0.5
YDSE	L, I, ζ	1, 0.01, 0.28
LSHADE	M_s_, P_s_, H	5, 5, 6
JADE	c, p, µCR, µF	0.1, 0.05, 0.5, 0.5
IDE-EDA	K, H, τ	3, 5, 0.9
APSM-jSO	F, CR, H, Arate	0.3, 0.8, 6, 1.3

**Table 2:** Details of MICFOA and three MICFOA-derived algorithms.

Strategy	MICFOA	MICFOA-1	MICFOA-2	MICFOA-3
LDIS	Yes	Yes	No	No
BSM	Yes	No	Yes	No
FPRS	Yes	No	No	Yes

**Table 3:** Details of MICFOA and three MICFOA-derived algorithms.

Algorithm	CEC2017 Test Set				CEC2022 Test Set		Mean Ranking
	D = 10	D = 30	D = 50	D = 100	D = 10	D = 20	
CFOA	4.517	4.517	4.552	4.517	4.500	4.583	4.531
MICFOA-1	3.621	3.621	3.655	3.103	3.583	3.167	3.458
MICFOA-2	3.552	3.483	3.207	3.345	3.250	3.583	3.403
MICFOA-3	2.000	2.000	2.207	2.690	1.833	2.083	2.136
MICFOA	1.310	1.379	1.379	1.345	1.833	1.583	1.472
*p*-value	2.31 × 10^−16^	1.26 × 10^−15^	1.28 × 10^−14^	1.70 × 10^−12^	3.47 × 10^−05^	1.60 × 10^−05^	N/A

**Table 4:** The results of Wilcoxon rank sum test between MICFOA and derived algorithms.

vs.CFOA +/=/−	CEC2017 Test Set				CEC2022 Test Set	
	D = 10	D = 30	D = 50	D = 100	D = 10	D = 20
MICFOA-1	6/20/3	15/8/6	18/5/6	20/5/4	5/5/2	7/3/2
MICFOA-2	8/18/3	20/8/1	20/8/1	19/9/1	5/6/1	6/5/1
MICFOA-3	25/4/0	29/0/0	27/2/0	28/1/0	11/1/0	12/0/0
MICFOA	29/0/0	28/0/1	27/1/1	26/1/2	10/2/0	11/1/0

**Table 5:** The results of the Wilcoxon rank sum test between MICFOA and other competitors.

MICFOA vs. +/=/−	CEC2017 Test Set				
	D = 10	D = 30	D = 50	D = 100	Total
CFOA	28/1/0	28/1/0	27/1/1	28/0/1	111/3/2
PEOA	27/2/0	25/1/3	23/2/4	23/3/3	98/8/10
TLBO	27/1/1	22/4/3	24/4/1	26/3/0	99/12/5
COA	29/0/0	28/1/0	29/0/0	26/1/2	112/2/2
ARO	24/3/2	28/0/1	26/1/2	25/1/3	103/5/8
EDO	28/1/0	29/0/0	29/0/0	29/0/0	115/1/0
YDSE	27/1/1	29/0/0	29/0/0	29/0/0	114/1/1
LSHADE	23/3/3	26/1/2	23/3/3	24/3/2	96/10/10
JADE	17/5/7	20/5/4	19/5/5	18/4/7	74/19/23
IDE-EDA	14/10/5	18/7/4	15/10/4	22/6/1	69/33/14
APSM-JSO	17/8/4	18/8/3	19/7/3	22/6/1	76/29/11

**Table 6:** The results of the Friedman test between MICFOA and other competitors.

Algorithm	CEC2017 Test Set				Mean Ranking
	D = 10 ^a^	D = 30 ^b^	D = 50 ^c^	D = 100 ^d^	
MICFOA	2.17	2.07	2.10	1.93	2.07
CFOA	7.14	7.48	7.17	7.69	7.37
PEOA	8.38	7.59	7.10	6.55	7.41
TLBO	6.79	5.24	5.48	5.76	5.82
COA	10.62	9.14	9.10	8.59	9.36
ARO	6.52	6.21	5.72	5.86	6.08
EDO	9.45	11.03	11.14	11.21	10.71
YDSE	6.66	8.52	9.00	9.10	8.32
LSHADE	7.21	9.07	9.00	8.90	8.54
JADE	4.52	5.48	5.62	4.72	5.09
IDE-EDA	3.97	2.76	2.97	3.52	3.30
APSM-JSO	4.59	3.41	3.59	4.17	3.94

^a^ The *p*-value of the Friedman test is 1.31 × 10^−24^, and the chi-square is 140.46. ^b^ The *p*-value of the Friedman test is 2.96 × 10^−35^, and the chi-square is 192.28. ^c^ The *p*-value of the Friedman test is 2.16 × 10^−34^, and the chi-square is 188.11. ^d^ The *p*-value of the Friedman test is 2.78 × 10^−32^, and the chi-square is 177.89.

**Table 7:** The results of the Wilcoxon rank sum test between MICFOA and other competitors.

MICFOA vs. +/=/−	CEC2022 Test Set		
	D = 10	D = 20	Total
CFOA	11/1/0	12/0/0	23/1/0
PEOA	9/2/1	10/1/1	19/3/2
TLBO	8/1/3	9/1/2	17/2/5
COA	11/0/1	12/0/0	23/0/1
ARO	8/3/1	11/0/1	19/3/4
EDO	10/0/2	12/0/0	22/0/2
YDSE	10/1/1	12/0/0	22/1/1
LSHADE	8/2/2	7/1/4	15/3/6
JADE	4/4/4	6/2/4	10/6/8
IDE-EDA	6/5/1	5/4/3	11/9/4
APSM-JSO	6/4/2	9/1/2	15/5/4

**Table 8:** The results of the Friedman test between MICFOA and other competitors.

Algorithm	CEC2022 Test Set		Mean Ranking
	D = 10	D = 20	
MICFOA	3.08	2.58	2.83
CFOA	7.17	7.33	7.25
PEOA	7.92	8.00	7.96
TLBO	5.92	5.33	5.63
COA	10.50	9.92	10.21
ARO	6.83	6.33	6.58
EDO	8.83	11.33	10.08
YDSE	7.42	8.17	7.79
LSHADE	6.42	6.50	6.46
JADE	4.67	4.42	4.54
IDE-EDA	4.67	3.92	4.29
APSM-JSO	4.58	4.17	4.38
*p*-value	7.61 × 10^−6^	2.62 × 10^−10^	N/A

## Data Availability

Data will be made available on request.

## Appendix A

**Table A1:** Comparison of results on CEC 2017 (D = 10).

Function	Metric	MICFOA	CFOA	PEOA	TLBO	COA	ARO	EDO	YDSE	LSHADE	JADE	IDE-EDA	APSM-JSO
F1	Best	1.0000 × 10^+02^	1.1669 × 10^+02^	1.0006 × 10^+02^	1.1950 × 10^+02^	6.6271 × 10^+02^	1.0912 × 10^+02^	1.7708 × 10^+06^	6.0970 × 10^+04^	1.0029 × 10^+02^	1.0000 × 10^+02^	1.0000 × 10^+02^	1.1669 × 10^+02^
	Mean	1.0005 × 10^+02^	1.3922 × 10^+03^	1.8143 × 10^+03^	2.4137 × 10^+03^	5.0787 × 10^+03^	1.2763 × 10^+03^	1.8673 × 10^+07^	1.6407 × 10^+05^	2.7321 × 10^+04^	1.0000 × 10^+02^	1.0005 × 10^+02^	1.3922 × 10^+03^
	Std	1.0530 × 10^−01^	1.9842 × 10^+03^	2.5125 × 10^+03^	2.2284 × 10^+03^	3.5780 × 10^+03^	1.3299 × 10^+03^	1.3434 × 10^+07^	8.3271 × 10^+04^	8.0106 × 10^+04^	4.9450 × 10^−03^	1.0530 × 10^−01^	1.9842 × 10^+03^
F2	Best	3.0000 × 10^+02^	3.3199 × 10^+02^	3.0000 × 10^+02^	3.0000 × 10^+02^	3.9933 × 10^+02^	3.0726 × 10^+02^	3.1564 × 10^+02^	3.0067 × 10^+02^	3.0317 × 10^+02^	3.0000 × 10^+02^	3.0000 × 10^+02^	3.3199 × 10^+02^
	Mean	3.0000 × 10^+02^	8.5056 × 10^+02^	3.0000 × 10^+02^	3.0000 × 10^+02^	2.0311 × 10^+03^	4.1197 × 10^+02^	4.0130 × 10^+02^	3.0246 × 10^+02^	6.3668 × 10^+03^	3.0003 × 10^+02^	3.0000 × 10^+02^	8.5056 × 10^+02^
	Std	9.8897 × 10^−08^	4.8760 × 10^+02^	9.8731 × 10^−05^	2.3809 × 10^−11^	1.2258 × 10^+03^	1.4143 × 10^+02^	7.1155 × 10^+01^	1.1259 × 10^+00^	7.1356 × 10^+03^	6.9260 × 10^−02^	9.8897 × 10^−08^	4.8760 × 10^+02^
F3	Best	4.0000 × 10^+02^	4.0001 × 10^+02^	4.0003 × 10^+02^	4.0011 × 10^+02^	4.0001 × 10^+02^	4.0011 × 10^+02^	4.0530 × 10^+02^	4.0244 × 10^+02^	4.0141 × 10^+02^	4.0001 × 10^+02^	4.0000 × 10^+02^	4.0001 × 10^+02^
	Mean	4.0113 × 10^+02^	4.0540 × 10^+02^	4.0757 × 10^+02^	4.0205 × 10^+02^	4.1645 × 10^+02^	4.0442 × 10^+02^	4.1123 × 10^+02^	4.0494 × 10^+02^	4.0596 × 10^+02^	4.0326 × 10^+02^	4.0113 × 10^+02^	4.0540 × 10^+02^
	Std	8.5905 × 10^−01^	1.9294 × 10^+00^	1.1887 × 10^+01^	1.1321 × 10^+00^	2.4613 × 10^+01^	2.2908 × 10^+00^	5.8387 × 10^+00^	1.1458 × 10^+00^	1.5371 × 10^+00^	1.5956 × 10^+00^	8.5905 × 10^−01^	1.9294 × 10^+00^
F4	Best	5.0199 × 10^+02^	5.0578 × 10^+02^	5.0669 × 10^+02^	5.0398 × 10^+02^	5.0535 × 10^+02^	5.0597 × 10^+02^	5.2179 × 10^+02^	5.1367 × 10^+02^	5.0515 × 10^+02^	5.0935 × 10^+02^	5.0199 × 10^+02^	5.0578 × 10^+02^
	Mean	5.0618 × 10^+02^	5.1348 × 10^+02^	5.2783 × 10^+02^	5.1099 × 10^+02^	5.2405 × 10^+02^	5.1517 × 10^+02^	5.4520 × 10^+02^	5.2445 × 10^+02^	5.2206 × 10^+02^	5.1728 × 10^+02^	5.0618 × 10^+02^	5.1348 × 10^+02^
	Std	2.3366 × 10^+00^	4.7540 × 10^+00^	1.1657 × 10^+01^	5.7967 × 10^+00^	1.4123 × 10^+01^	5.3684 × 10^+00^	9.4406 × 10^+00^	5.8041 × 10^+00^	9.4418 × 10^+00^	3.2787 × 10^+00^	2.3366 × 10^+00^	4.7540 × 10^+00^
F5	Best	6.0000 × 10^+02^	6.0013 × 10^+02^	6.0149 × 10^+02^	6.0000 × 10^+02^	6.0002 × 10^+02^	6.0000 × 10^+02^	6.0630 × 10^+02^	6.0265 × 10^+02^	6.0000 × 10^+02^	6.0000 × 10^+02^	6.0000 × 10^+02^	6.0013 × 10^+02^
	Mean	6.0000 × 10^+02^	6.0209 × 10^+02^	6.1076 × 10^+02^	6.0044 × 10^+02^	6.1052 × 10^+02^	6.0011 × 10^+02^	6.1553 × 10^+02^	6.0669 × 10^+02^	6.0000 × 10^+02^	6.0000 × 10^+02^	6.0000 × 10^+02^	6.0209 × 10^+02^
	Std	9.4120 × 10^−03^	1.1531 × 10^+00^	5.2730 × 10^+00^	9.3028 × 10^−01^	1.6778 × 10^+01^	1.9730 × 10^−01^	5.5897 × 10^+00^	2.4821 × 10^+00^	1.0316 × 10^−02^	2.1377 × 10^−06^	9.4120 × 10^−03^	1.1531 × 10^+00^
F6	Best	7.1155 × 10^+02^	7.1576 × 10^+02^	7.2165 × 10^+02^	7.1425 × 10^+02^	7.2674 × 10^+02^	7.1872 × 10^+02^	7.4304 × 10^+02^	7.2570 × 10^+02^	7.1289 × 10^+02^	7.2407 × 10^+02^	7.1155 × 10^+02^	7.1576 × 10^+02^
	Mean	7.1590 × 10^+02^	7.2351 × 10^+02^	7.3665 × 10^+02^	7.2496 × 10^+02^	7.9054 × 10^+02^	7.3014 × 10^+02^	7.6323 × 10^+02^	7.3875 × 10^+02^	7.3123 × 10^+02^	7.3182 × 10^+02^	7.1590 × 10^+02^	7.2351 × 10^+02^
	Std	2.7690 × 10^+00^	4.4570 × 10^+00^	9.1670 × 10^+00^	6.0023 × 10^+00^	2.5869 × 10^+01^	6.7962 × 10^+00^	9.0686 × 10^+00^	7.1092 × 10^+00^	8.8628 × 10^+00^	3.2619 × 10^+00^	2.7690 × 10^+00^	4.4570 × 10^+00^
F7	Best	8.0199 × 10^+02^	8.0457 × 10^+02^	8.0613 × 10^+02^	8.0298 × 10^+02^	8.0895 × 10^+02^	8.0398 × 10^+02^	8.2574 × 10^+02^	8.1322 × 10^+02^	8.0299 × 10^+02^	8.0970 × 10^+02^	8.0199 × 10^+02^	8.0457 × 10^+02^
	Mean	8.0530 × 10^+02^	8.1074 × 10^+02^	8.1844 × 10^+02^	8.1051 × 10^+02^	8.2677 × 10^+02^	8.1549 × 10^+02^	8.3901 × 10^+02^	8.2175 × 10^+02^	8.1746 × 10^+02^	8.1748 × 10^+02^	8.0530 × 10^+02^	8.1074 × 10^+02^
	Std	1.8829 × 10^+00^	3.4842 × 10^+00^	7.0385 × 10^+00^	5.1003 × 10^+00^	8.6879 × 10^+00^	6.2593 × 10^+00^	6.2985 × 10^+00^	5.1382 × 10^+00^	9.5846 × 10^+00^	4.4432 × 10^+00^	1.8829 × 10^+00^	3.4842 × 10^+00^
F8	Best	9.0000 × 10^+02^	9.0000 × 10^+02^	9.0063 × 10^+02^	9.0000 × 10^+02^	9.0115 × 10^+02^	9.0000 × 10^+02^	9.1955 × 10^+02^	9.0384 × 10^+02^	9.0000 × 10^+02^	9.0000 × 10^+02^	9.0000 × 10^+02^	9.0000 × 10^+02^
	Mean	9.0002 × 10^+02^	9.0107 × 10^+02^	9.2457 × 10^+02^	9.0094 × 10^+02^	1.1267 × 10^+03^	9.0422 × 10^+02^	1.0001 × 10^+03^	9.3560 × 10^+02^	9.0009 × 10^+02^	9.0000 × 10^+02^	9.0002 × 10^+02^	9.0107 × 10^+02^
	Std	8.4907 × 10^−02^	1.8037 × 10^+00^	3.1283 × 10^+01^	1.4007 × 10^+00^	2.5917 × 10^+02^	9.0805 × 10^+00^	9.9134 × 10^+01^	2.5848 × 10^+01^	4.9886 × 10^−01^	0.0000 × 10^+00^	8.4907 × 10^−02^	1.8037 × 10^+00^
F9	Best	1.1654 × 10^+03^	1.3360 × 10^+03^	1.2283 × 10^+03^	1.1443 × 10^+03^	1.2758 × 10^+03^	1.1305 × 10^+03^	1.5708 × 10^+03^	1.5154 × 10^+03^	1.0540 × 10^+03^	1.6209 × 10^+03^	1.1654 × 10^+03^	1.3360 × 10^+03^
	Mean	1.5148 × 10^+03^	1.8290 × 10^+03^	1.7645 × 10^+03^	1.9135 × 10^+03^	2.1486 × 10^+03^	1.5287 × 10^+03^	1.9181 × 10^+03^	1.7833 × 10^+03^	2.1493 × 10^+03^	2.0364 × 10^+03^	1.5148 × 10^+03^	1.8290 × 10^+03^
	Std	1.9552 × 10^+02^	2.8374 × 10^+02^	3.0437 × 10^+02^	3.6490 × 10^+02^	3.9061 × 10^+02^	1.9877 × 10^+02^	1.4981 × 10^+02^	1.4820 × 10^+02^	3.9589 × 10^+02^	2.0544 × 10^+02^	1.9552 × 10^+02^	2.8374 × 10^+02^
F10	Best	1.1001 × 10^+03^	1.1074 × 10^+03^	1.1144 × 10^+03^	1.1056 × 10^+03^	1.1049 × 10^+03^	1.1034 × 10^+03^	1.1121 × 10^+03^	1.1075 × 10^+03^	1.1007 × 10^+03^	1.1008 × 10^+03^	1.1001 × 10^+03^	1.1074 × 10^+03^
	Mean	1.1029 × 10^+03^	1.1247 × 10^+03^	1.1425 × 10^+03^	1.1184 × 10^+03^	1.1594 × 10^+03^	1.1099 × 10^+03^	1.1305 × 10^+03^	1.1124 × 10^+03^	1.1055 × 10^+03^	1.1029 × 10^+03^	1.1029 × 10^+03^	1.1247 × 10^+03^
	Std	1.4200 × 10^+00^	1.3603 × 10^+01^	2.0865 × 10^+01^	1.5942 × 10^+01^	6.8992 × 10^+01^	4.9995 × 10^+00^	1.0004 × 10^+01^	2.8054 × 10^+00^	2.7371 × 10^+00^	1.9239 × 10^+00^	1.4200 × 10^+00^	1.3603 × 10^+01^
F11	Best	1.2502 × 10^+03^	5.3458 × 10^+03^	1.2031 × 10^+04^	2.8533 × 10^+03^	4.5426 × 10^+03^	2.1213 × 10^+03^	9.9070 × 10^+03^	1.7928 × 10^+03^	1.6455 × 10^+03^	2.9625 × 10^+03^	1.2502 × 10^+03^	5.3458 × 10^+03^
	Mean	1.3787 × 10^+03^	3.1148 × 10^+04^	6.7869 × 10^+05^	1.7549 × 10^+04^	2.5006 × 10^+05^	5.9796 × 10^+04^	3.7581 × 10^+04^	2.3833 × 10^+03^	4.0401 × 10^+05^	1.1385 × 10^+04^	1.3787 × 10^+03^	3.1148 × 10^+04^
	Std	8.0009 × 10^+01^	4.5753 × 10^+04^	6.7072 × 10^+05^	1.0997 × 10^+04^	7.0200 × 10^+05^	2.1983 × 10^+05^	1.8770 × 10^+04^	2.7740 × 10^+02^	6.4219 × 10^+05^	5.0685 × 10^+03^	8.0009 × 10^+01^	4.5753 × 10^+04^
F12	Best	1.3007 × 10^+03^	1.7401 × 10^+03^	2.0286 × 10^+03^	1.5372 × 10^+03^	1.5898 × 10^+03^	1.3096 × 10^+03^	1.3503 × 10^+03^	1.3239 × 10^+03^	1.3081 × 10^+03^	1.3027 × 10^+03^	1.3007 × 10^+03^	1.7401 × 10^+03^
	Mean	1.3138 × 10^+03^	3.9716 × 10^+03^	1.3576 × 10^+04^	4.9030 × 10^+03^	9.2522 × 10^+03^	4.2000 × 10^+03^	1.3863 × 10^+03^	1.3397 × 10^+03^	1.6901 × 10^+03^	1.3137 × 10^+03^	1.3138 × 10^+03^	3.9716 × 10^+03^
	Std	4.3952 × 10^+00^	1.9904 × 10^+03^	8.1529 × 10^+03^	3.2340 × 10^+03^	9.1502 × 10^+03^	5.1837 × 10^+03^	1.8115 × 10^+01^	1.1163 × 10^+01^	1.6761 × 10^+03^	1.2025 × 10^+01^	4.3952 × 10^+00^	1.9904 × 10^+03^
F13	Best	1.4000 × 10^+03^	1.4305 × 10^+03^	1.4436 × 10^+03^	1.4408 × 10^+03^	1.4516 × 10^+03^	1.4020 × 10^+03^	1.4254 × 10^+03^	1.4190 × 10^+03^	1.4023 × 10^+03^	1.4020 × 10^+03^	1.4000 × 10^+03^	1.4305 × 10^+03^
	Mean	1.4091 × 10^+03^	1.4911 × 10^+03^	1.5319 × 10^+03^	1.4757 × 10^+03^	1.7342 × 10^+03^	1.4317 × 10^+03^	1.4312 × 10^+03^	1.4281 × 10^+03^	1.4220 × 10^+03^	1.4138 × 10^+03^	1.4091 × 10^+03^	1.4911 × 10^+03^
	Std	7.7426 × 10^+00^	4.8864 × 10^+01^	1.2537 × 10^+02^	2.8851 × 10^+01^	2.8185 × 10^+02^	1.0385 × 10^+02^	3.2427 × 10^+00^	2.8865 × 10^+00^	6.1830 × 10^+00^	9.2866 × 10^+00^	7.7426 × 10^+00^	4.8864 × 10^+01^
F14	Best	1.5006 × 10^+03^	1.6361 × 10^+03^	1.7177 × 10^+03^	1.5318 × 10^+03^	1.6367 × 10^+03^	1.5011 × 10^+03^	1.5071 × 10^+03^	1.5069 × 10^+03^	1.5016 × 10^+03^	1.5002 × 10^+03^	1.5006 × 10^+03^	1.6361 × 10^+03^
	Mean	1.5022 × 10^+03^	2.2399 × 10^+03^	2.2000 × 10^+03^	1.6917 × 10^+03^	3.0223 × 10^+03^	1.5258 × 10^+03^	1.5209 × 10^+03^	1.5125 × 10^+03^	1.5072 × 10^+03^	1.5028 × 10^+03^	1.5022 × 10^+03^	2.2399 × 10^+03^
	Std	1.1398 × 10^+00^	1.2708 × 10^+03^	4.8564 × 10^+02^	9.6804 × 10^+01^	1.9929 × 10^+03^	4.8488 × 10^+01^	6.1567 × 10^+00^	3.7212 × 10^+00^	5.8141 × 10^+00^	4.3185 × 10^+00^	1.1398 × 10^+00^	1.2708 × 10^+03^
F15	Best	1.6008 × 10^+03^	1.6078 × 10^+03^	1.6052 × 10^+03^	1.6017 × 10^+03^	1.6071 × 10^+03^	1.6004 × 10^+03^	1.6325 × 10^+03^	1.6106 × 10^+03^	1.6010 × 10^+03^	1.6017 × 10^+03^	1.6008 × 10^+03^	1.6078 × 10^+03^
	Mean	1.6033 × 10^+03^	1.6575 × 10^+03^	1.7211 × 10^+03^	1.6259 × 10^+03^	1.7398 × 10^+03^	1.7091 × 10^+03^	1.7215 × 10^+03^	1.6753 × 10^+03^	1.6223 × 10^+03^	1.6187 × 10^+03^	1.6033 × 10^+03^	1.6575 × 10^+03^
	Std	3.2917 × 10^+00^	5.8895 × 10^+01^	8.6271 × 10^+01^	4.3344 × 10^+01^	1.2146 × 10^+02^	1.0340 × 10^+02^	4.9312 × 10^+01^	4.6093 × 10^+01^	2.0773 × 10^+01^	4.1148 × 10^+01^	3.2917 × 10^+00^	5.8895 × 10^+01^
F16	Best	1.7019 × 10^+03^	1.7323 × 10^+03^	1.7312 × 10^+03^	1.7065 × 10^+03^	1.7091 × 10^+03^	1.7014 × 10^+03^	1.7529 × 10^+03^	1.7348 × 10^+03^	1.7032 × 10^+03^	1.7007 × 10^+03^	1.7019 × 10^+03^	1.7323 × 10^+03^
	Mean	1.7287 × 10^+03^	1.7499 × 10^+03^	1.7631 × 10^+03^	1.7438 × 10^+03^	1.7559 × 10^+03^	1.7235 × 10^+03^	1.7827 × 10^+03^	1.7651 × 10^+03^	1.7281 × 10^+03^	1.7162 × 10^+03^	1.7287 × 10^+03^	1.7499 × 10^+03^
	Std	1.2756 × 10^+01^	1.3258 × 10^+01^	1.8297 × 10^+01^	1.9899 × 10^+01^	2.6681 × 10^+01^	2.1340 × 10^+01^	1.6387 × 10^+01^	1.3359 × 10^+01^	6.8811 × 10^+00^	9.2780 × 10^+00^	1.2756 × 10^+01^	1.3258 × 10^+01^
F17	Best	1.8053 × 10^+03^	2.1097 × 10^+03^	1.9210 × 10^+03^	2.4266 × 10^+03^	2.4591 × 10^+03^	1.8043 × 10^+03^	1.8324 × 10^+03^	1.8247 × 10^+03^	1.8034 × 10^+03^	1.8007 × 10^+03^	1.8053 × 10^+03^	2.1097 × 10^+03^
	Mean	1.8129 × 10^+03^	5.0447 × 10^+03^	1.6233 × 10^+04^	8.1866 × 10^+03^	1.6310 × 10^+04^	2.0171 × 10^+03^	1.8625 × 10^+03^	1.8314 × 10^+03^	1.8398 × 10^+03^	1.8169 × 10^+03^	1.8129 × 10^+03^	5.0447 × 10^+03^
	Std	3.8518 × 10^+00^	2.5679 × 10^+03^	1.0316 × 10^+04^	5.9971 × 10^+03^	1.1611 × 10^+04^	5.4003 × 10^+02^	1.6453 × 10^+01^	3.0079 × 10^+00^	6.4269 × 10^+01^	1.4397 × 10^+01^	3.8518 × 10^+00^	2.5679 × 10^+03^
F18	Best	1.9012 × 10^+03^	1.9320 × 10^+03^	1.9488 × 10^+03^	1.9172 × 10^+03^	1.9242 × 10^+03^	1.9006 × 10^+03^	1.9065 × 10^+03^	1.9046 × 10^+03^	1.9011 × 10^+03^	1.9001 × 10^+03^	1.9012 × 10^+03^	1.9320 × 10^+03^
	Mean	1.9030 × 10^+03^	2.1253 × 10^+03^	4.2013 × 10^+03^	2.0645 × 10^+03^	3.8782 × 10^+03^	1.9219 × 10^+03^	1.9096 × 10^+03^	1.9064 × 10^+03^	1.9028 × 10^+03^	1.9015 × 10^+03^	1.9030 × 10^+03^	2.1253 × 10^+03^
	Std	6.8807 × 10^−01^	2.6745 × 10^+02^	1.7345 × 10^+03^	1.4614 × 10^+02^	2.5493 × 10^+03^	1.0380 × 10^+02^	1.6026 × 10^+00^	1.0266 × 10^+00^	9.5192 × 10^−01^	1.3603 × 10^+00^	6.8807 × 10^−01^	2.6745 × 10^+02^
F19	Best	2.0003 × 10^+03^	2.0258 × 10^+03^	2.0343 × 10^+03^	2.0026 × 10^+03^	2.0201 × 10^+03^	2.0001 × 10^+03^	2.0801 × 10^+03^	2.0410 × 10^+03^	2.0000 × 10^+03^	2.0000 × 10^+03^	2.0003 × 10^+03^	2.0258 × 10^+03^
	Mean	2.0152 × 10^+03^	2.0479 × 10^+03^	2.0627 × 10^+03^	2.0249 × 10^+03^	2.0462 × 10^+03^	2.0133 × 10^+03^	2.1369 × 10^+03^	2.0747 × 10^+03^	2.0132 × 10^+03^	2.0080 × 10^+03^	2.0152 × 10^+03^	2.0479 × 10^+03^
	Std	1.0434 × 10^+01^	1.7333 × 10^+01^	1.6311 × 10^+01^	1.0630 × 10^+01^	4.2668 × 10^+01^	1.1941 × 10^+01^	3.2108 × 10^+01^	1.5884 × 10^+01^	8.6871 × 10^+00^	9.6705 × 10^+00^	1.0434 × 10^+01^	1.7333 × 10^+01^
F20	Best	2.2000 × 10^+03^	2.2003 × 10^+03^	2.2007 × 10^+03^	2.2003 × 10^+03^	2.2006 × 10^+03^	2.2003 × 10^+03^	2.2012 × 10^+03^	2.2013 × 10^+03^	2.2004 × 10^+03^	2.2000 × 10^+03^	2.2000 × 10^+03^	2.2003 × 10^+03^
	Mean	2.2498 × 10^+03^	2.2688 × 10^+03^	2.2021 × 10^+03^	2.2560 × 10^+03^	2.3106 × 10^+03^	2.2755 × 10^+03^	2.2230 × 10^+03^	2.2118 × 10^+03^	2.3142 × 10^+03^	2.2820 × 10^+03^	2.2498 × 10^+03^	2.2688 × 10^+03^
	Std	5.4121 × 10^+01^	5.5405 × 10^+01^	9.0736 × 10^−01^	5.3233 × 10^+01^	3.7877 × 10^+01^	5.6494 × 10^+01^	3.0206 × 10^+01^	2.3872 × 10^+01^	3.2699 × 10^+01^	5.6794 × 10^+01^	5.4121 × 10^+01^	5.5405 × 10^+01^
F21	Best	2.2112 × 10^+03^	2.2164 × 10^+03^	2.2276 × 10^+03^	2.3003 × 10^+03^	2.2517 × 10^+03^	2.3003 × 10^+03^	2.2461 × 10^+03^	2.2361 × 10^+03^	2.3000 × 10^+03^	2.3000 × 10^+03^	2.2112 × 10^+03^	2.2164 × 10^+03^
	Mean	2.2977 × 10^+03^	2.2991 × 10^+03^	2.2981 × 10^+03^	2.3024 × 10^+03^	2.3023 × 10^+03^	2.3021 × 10^+03^	2.3050 × 10^+03^	2.3002 × 10^+03^	2.3024 × 10^+03^	2.3006 × 10^+03^	2.2977 × 10^+03^	2.2991 × 10^+03^
	Std	1.6344 × 10^+01^	2.2327 × 10^+01^	2.9238 × 10^+01^	2.0696 × 10^+00^	1.1412 × 10^+01^	1.0804 × 10^+00^	2.3056 × 10^+01^	2.2879 × 10^+01^	2.0735 × 10^+00^	6.0791 × 10^−01^	1.6344 × 10^+01^	2.2327 × 10^+01^
F22	Best	2.6001 × 10^+03^	2.6065 × 10^+03^	2.3004 × 10^+03^	2.6025 × 10^+03^	2.6092 × 10^+03^	2.6085 × 10^+03^	2.6276 × 10^+03^	2.6148 × 10^+03^	2.6123 × 10^+03^	2.6032 × 10^+03^	2.6001 × 10^+03^	2.6065 × 10^+03^
	Mean	2.6075 × 10^+03^	2.6144 × 10^+03^	2.6204 × 10^+03^	2.6148 × 10^+03^	2.6235 × 10^+03^	2.6229 × 10^+03^	2.6433 × 10^+03^	2.6258 × 10^+03^	2.6298 × 10^+03^	2.6142 × 10^+03^	2.6075 × 10^+03^	2.6144 × 10^+03^
	Std	3.2083 × 10^+00^	5.2316 × 10^+00^	6.1411 × 10^+01^	5.5941 × 10^+00^	8.0505 × 10^+00^	8.5666 × 10^+00^	6.4893 × 10^+00^	5.0897 × 10^+00^	1.0510 × 10^+01^	5.1383 × 10^+00^	3.2083 × 10^+00^	5.2316 × 10^+00^
F23	Best	2.5000 × 10^+03^	2.5025 × 10^+03^	2.5012 × 10^+03^	2.7000 × 10^+03^	2.7399 × 10^+03^	2.5000 × 10^+03^	2.5943 × 10^+03^	2.5596 × 10^+03^	2.5034 × 10^+03^	2.5000 × 10^+03^	2.5000 × 10^+03^	2.5025 × 10^+03^
	Mean	2.6994 × 10^+03^	2.7278 × 10^+03^	2.5789 × 10^+03^	2.7400 × 10^+03^	2.7534 × 10^+03^	2.7072 × 10^+03^	2.7547 × 10^+03^	2.7294 × 10^+03^	2.7494 × 10^+03^	2.7288 × 10^+03^	2.6994 × 10^+03^	2.7278 × 10^+03^
	Std	8.3198 × 10^+01^	4.2748 × 10^+01^	1.1993 × 10^+02^	9.6855 × 10^+00^	1.2329 × 10^+01^	9.4746 × 10^+01^	5.2250 × 10^+01^	5.8128 × 10^+01^	4.7718 × 10^+01^	6.2407 × 10^+01^	8.3198 × 10^+01^	4.2748 × 10^+01^
F24	Best	2.8977 × 10^+03^	2.8977 × 10^+03^	2.8983 × 10^+03^	2.8978 × 10^+03^	2.6025 × 10^+03^	2.8978 × 10^+03^	2.9014 × 10^+03^	2.8990 × 10^+03^	2.8983 × 10^+03^	2.8977 × 10^+03^	2.8977 × 10^+03^	2.8977 × 10^+03^
	Mean	2.9105 × 10^+03^	2.9308 × 10^+03^	2.9301 × 10^+03^	2.9237 × 10^+03^	2.9220 × 10^+03^	2.9303 × 10^+03^	2.9277 × 10^+03^	2.9083 × 10^+03^	2.9339 × 10^+03^	2.9258 × 10^+03^	2.9105 × 10^+03^	2.9308 × 10^+03^
	Std	2.0450 × 10^+01^	2.1207 × 10^+01^	2.2100 × 10^+01^	2.3578 × 10^+01^	6.6411 × 10^+01^	2.2343 × 10^+01^	1.7252 × 10^+01^	1.3933 × 10^+01^	1.9262 × 10^+01^	2.2655 × 10^+01^	2.0450 × 10^+01^	2.1207 × 10^+01^
F25	Best	2.6000 × 10^+03^	2.8013 × 10^+03^	2.8215 × 10^+03^	2.8000 × 10^+03^	2.8001 × 10^+03^	2.6003 × 10^+03^	2.8634 × 10^+03^	2.7380 × 10^+03^	2.9000 × 10^+03^	2.8000 × 10^+03^	2.6000 × 10^+03^	2.8013 × 10^+03^
	Mean	2.8992 × 10^+03^	2.9195 × 10^+03^	2.9054 × 10^+03^	2.9338 × 10^+03^	3.2004 × 10^+03^	2.8961 × 10^+03^	2.9606 × 10^+03^	2.9100 × 10^+03^	2.9944 × 10^+03^	2.8965 × 10^+03^	2.8992 × 10^+03^	2.9195 × 10^+03^
	Std	6.0589 × 10^+01^	5.7689 × 10^+01^	4.0211 × 10^+01^	8.7536 × 10^+01^	4.0501 × 10^+02^	7.0216 × 10^+01^	3.1724 × 10^+01^	4.1252 × 10^+01^	1.4458 × 10^+02^	2.8812 × 10^+01^	6.0589 × 10^+01^	5.7689 × 10^+01^
F26	Best	3.0889 × 10^+03^	3.0895 × 10^+03^	3.0901 × 10^+03^	3.0890 × 10^+03^	3.0895 × 10^+03^	3.0916 × 10^+03^	3.0921 × 10^+03^	3.0904 × 10^+03^	3.0711 × 10^+03^	3.0934 × 10^+03^	3.0889 × 10^+03^	3.0895 × 10^+03^
	Mean	3.0906 × 10^+03^	3.0942 × 10^+03^	3.0961 × 10^+03^	3.0927 × 10^+03^	3.1096 × 10^+03^	3.1009 × 10^+03^	3.0955 × 10^+03^	3.0927 × 10^+03^	3.0724 × 10^+03^	3.0965 × 10^+03^	3.0906 × 10^+03^	3.0942 × 10^+03^
	Std	1.9634 × 10^+00^	2.5529 × 10^+00^	3.5116 × 10^+00^	3.4104 × 10^+00^	2.2538 × 10^+01^	7.9561 × 10^+00^	2.0250 × 10^+00^	1.1444 × 10^+00^	1.1291 × 10^+00^	6.1190 × 10^+00^	1.9634 × 10^+00^	2.5529 × 10^+00^
F27	Best	3.1000 × 10^+03^	3.1003 × 10^+03^	3.1019 × 10^+03^	3.1000 × 10^+03^	3.1045 × 10^+03^	2.8000 × 10^+03^	3.1560 × 10^+03^	3.1275 × 10^+03^	3.2725 × 10^+03^	3.1000 × 10^+03^	3.1000 × 10^+03^	3.1003 × 10^+03^
	Mean	3.1032 × 10^+03^	3.2379 × 10^+03^	3.1833 × 10^+03^	3.2359 × 10^+03^	3.3649 × 10^+03^	3.2003 × 10^+03^	3.2814 × 10^+03^	3.1665 × 10^+03^	3.2755 × 10^+03^	3.2775 × 10^+03^	3.1032 × 10^+03^	3.2379 × 10^+03^
	Std	1.7631 × 10^+01^	1.2899 × 10^+02^	1.0777 × 10^+02^	1.2995 × 10^+02^	1.4669 × 10^+02^	1.7276 × 10^+02^	1.4133 × 10^+02^	1.9108 × 10^+01^	6.3127 × 10^+00^	1.4180 × 10^+02^	1.7631 × 10^+01^	1.2899 × 10^+02^
F28	Best	3.1362 × 10^+03^	3.1453 × 10^+03^	3.1433 × 10^+03^	3.1564 × 10^+03^	3.1589 × 10^+03^	3.1397 × 10^+03^	3.1808 × 10^+03^	3.1383 × 10^+03^	3.1590 × 10^+03^	3.1610 × 10^+03^	3.1362 × 10^+03^	3.1453 × 10^+03^
	Mean	3.1527 × 10^+03^	3.1879 × 10^+03^	3.2172 × 10^+03^	3.1929 × 10^+03^	3.2226 × 10^+03^	3.1847 × 10^+03^	3.2383 × 10^+03^	3.1874 × 10^+03^	3.2147 × 10^+03^	3.1839 × 10^+03^	3.1527 × 10^+03^	3.1879 × 10^+03^
	Std	1.2497 × 10^+01^	3.0467 × 10^+01^	3.7575 × 10^+01^	3.8568 × 10^+01^	4.4668 × 10^+01^	2.6334 × 10^+01^	4.2185 × 10^+01^	3.0143 × 10^+01^	4.3286 × 10^+01^	1.4081 × 10^+01^	1.2497 × 10^+01^	3.0467 × 10^+01^
F29	Best	3.4040 × 10^+03^	4.3963 × 10^+03^	4.7175 × 10^+03^	7.4088 × 10^+03^	4.4875 × 10^+03^	4.6238 × 10^+03^	3.8651 × 10^+03^	3.9087 × 10^+03^	3.2033 × 10^+03^	4.7568 × 10^+03^	3.4040 × 10^+03^	4.3963 × 10^+03^
	Mean	4.2590 × 10^+03^	9.6716 × 10^+04^	6.2174 × 10^+05^	3.4688 × 10^+05^	5.9819 × 10^+05^	2.1717 × 10^+05^	4.5821 × 10^+04^	3.7407 × 10^+04^	4.1094 × 10^+03^	1.2201 × 10^+05^	4.2590 × 10^+03^	9.6716 × 10^+04^
	Std	4.5316 × 10^+03^	2.8246 × 10^+05^	1.1833 × 10^+06^	4.3634 × 10^+05^	7.1192 × 10^+05^	4.0510 × 10^+05^	1.1932 × 10^+05^	8.3213 × 10^+04^	1.6638 × 10^+03^	2.9084 × 10^+05^	4.5316 × 10^+03^	2.8246 × 10^+05^

**Table A2:** Comparison of results on CEC 2017 (D = 30).

Function	Metric	MICFOA	CFOA	PEOA	TLBO	COA	ARO	EDO	YDSE	LSHADE	JADE	IDE-EDA	APSM-JSO
F1	Best	1.4153 × 10^+04^	1.2912 × 10^+08^	1.9838 × 10^+02^	5.0632 × 10^+02^	2.4560 × 10^+07^	8.8647 × 10^+06^	1.3591 × 10^+10^	5.3427 × 10^+08^	3.5073 × 10^+03^	1.2377 × 10^+02^	2.3920 × 10^+03^	3.7583 × 10^+03^
	Mean	7.0730 × 10^+04^	5.5694 × 10^+08^	3.0508 × 10^+03^	3.1228 × 10^+04^	7.4229 × 10^+08^	3.4843 × 10^+07^	2.2022 × 10^+10^	1.2422 × 10^+09^	1.6492 × 10^+08^	3.7683 × 10^+03^	7.7696 × 10^+04^	5.1539 × 10^+04^
	Std	2.8627 × 10^+04^	2.0734 × 10^+08^	3.4544 × 10^+03^	6.8397 × 10^+04^	9.8313 × 10^+08^	2.5439 × 10^+07^	6.0387 × 10^+09^	5.2794 × 10^+08^	4.4175 × 10^+08^	4.0614 × 10^+03^	1.5959 × 10^+05^	6.2960 × 10^+04^
F2	Best	1.3553 × 10^+03^	3.4930 × 10^+04^	1.1182 × 10^+03^	2.8371 × 10^+04^	7.3265 × 10^+04^	4.0585 × 10^+04^	2.0411 × 10^+04^	2.1156 × 10^+04^	7.8424 × 10^+04^	4.9472 × 10^+04^	3.3318 × 10^+03^	2.4164 × 10^+03^
	Mean	4.9646 × 10^+03^	5.8934 × 10^+04^	2.8670 × 10^+03^	5.0734 × 10^+04^	1.1275 × 10^+05^	5.7993 × 10^+04^	4.0155 × 10^+04^	3.7793 × 10^+04^	2.3514 × 10^+05^	7.3342 × 10^+04^	1.2927 × 10^+04^	1.1577 × 10^+04^
	Std	2.4562 × 10^+03^	1.2317 × 10^+04^	9.2669 × 10^+02^	1.1556 × 10^+04^	3.8752 × 10^+04^	9.0882 × 10^+03^	9.7441 × 10^+03^	1.1572 × 10^+04^	9.1838 × 10^+04^	1.4399 × 10^+04^	4.8231 × 10^+03^	4.5503 × 10^+03^
F3	Best	4.6741 × 10^+02^	5.4664 × 10^+02^	4.9491 × 10^+02^	4.3476 × 10^+02^	5.2184 × 10^+02^	4.9502 × 10^+02^	1.0884 × 10^+03^	5.8395 × 10^+02^	4.2599 × 10^+02^	4.6765 × 10^+02^	4.1552 × 10^+02^	4.6847 × 10^+02^
	Mean	5.0750 × 10^+02^	6.3791 × 10^+02^	5.3202 × 10^+02^	5.0610 × 10^+02^	6.3037 × 10^+02^	5.5008 × 10^+02^	2.8710 × 10^+03^	6.4017 × 10^+02^	6.4790 × 10^+02^	5.0095 × 10^+02^	4.9964 × 10^+02^	5.0339 × 10^+02^
	Std	2.6506 × 10^+01^	6.2229 × 10^+01^	2.5840 × 10^+01^	2.6246 × 10^+01^	1.1086 × 10^+02^	3.3034 × 10^+01^	1.1865 × 10^+03^	3.8262 × 10^+01^	8.0451 × 10^+02^	2.4659 × 10^+01^	2.8112 × 10^+01^	2.5734 × 10^+01^
F4	Best	5.3698 × 10^+02^	6.1364 × 10^+02^	6.4470 × 10^+02^	5.7069 × 10^+02^	6.1328 × 10^+02^	5.7641 × 10^+02^	7.6476 × 10^+02^	6.5456 × 10^+02^	5.4199 × 10^+02^	6.6684 × 10^+02^	5.4423 × 10^+02^	5.3992 × 10^+02^
	Mean	5.6692 × 10^+02^	6.7850 × 10^+02^	7.0292 × 10^+02^	6.2024 × 10^+02^	7.6961 × 10^+02^	6.2364 × 10^+02^	8.3911 × 10^+02^	7.1500 × 10^+02^	6.7617 × 10^+02^	6.9413 × 10^+02^	5.7866 × 10^+02^	6.0154 × 10^+02^
	Std	2.3345 × 10^+01^	3.3533 × 10^+01^	3.0018 × 10^+01^	2.5104 × 10^+01^	5.3925 × 10^+01^	2.6727 × 10^+01^	3.5776 × 10^+01^	2.4986 × 10^+01^	5.5087 × 10^+01^	1.3382 × 10^+01^	2.2986 × 10^+01^	3.3458 × 10^+01^
F5	Best	6.0250 × 10^+02^	6.1807 × 10^+02^	6.2847 × 10^+02^	6.0764 × 10^+02^	6.3056 × 10^+02^	6.0493 × 10^+02^	6.4378 × 10^+02^	6.3512 × 10^+02^	6.0000 × 10^+02^	6.0000 × 10^+02^	6.0262 × 10^+02^	6.0181 × 10^+02^
	Mean	6.0621 × 10^+02^	6.3012 × 10^+02^	6.5181 × 10^+02^	6.1658 × 10^+02^	6.5448 × 10^+02^	6.1353 × 10^+02^	6.6708 × 10^+02^	6.4394 × 10^+02^	6.0668 × 10^+02^	6.0013 × 10^+02^	6.0619 × 10^+02^	6.0700 × 10^+02^
	Std	2.2553 × 10^+00^	6.6788 × 10^+00^	8.9084 × 10^+00^	5.2932 × 10^+00^	1.0896 × 10^+01^	6.3327 × 10^+00^	1.1060 × 10^+01^	6.9462 × 10^+00^	1.0742 × 10^+01^	2.5644 × 10^−01^	2.1993 × 10^+00^	4.6405 × 10^+00^
F6	Best	7.8307 × 10^+02^	9.0617 × 10^+02^	9.6863 × 10^+02^	7.9326 × 10^+02^	9.4513 × 10^+02^	8.5122 × 10^+02^	1.0839 × 10^+03^	9.7237 × 10^+02^	8.4620 × 10^+02^	9.0327 × 10^+02^	8.0396 × 10^+02^	8.2873 × 10^+02^
	Mean	8.2817 × 10^+02^	9.6694 × 10^+02^	1.0303 × 10^+03^	9.1334 × 10^+02^	1.2026 × 10^+03^	9.5195 × 10^+02^	1.2341 × 10^+03^	1.0187 × 10^+03^	9.3394 × 10^+02^	9.2598 × 10^+02^	8.6338 × 10^+02^	8.9802 × 10^+02^
	Std	2.2710 × 10^+01^	5.1346 × 10^+01^	4.4686 × 10^+01^	5.3918 × 10^+01^	1.1023 × 10^+02^	5.9893 × 10^+01^	7.6294 × 10^+01^	2.7214 × 10^+01^	3.7730 × 10^+01^	1.2543 × 10^+01^	4.5135 × 10^+01^	4.1709 × 10^+01^
F7	Best	8.4104 × 10^+02^	8.9284 × 10^+02^	8.9877 × 10^+02^	8.5314 × 10^+02^	9.3167 × 10^+02^	8.5681 × 10^+02^	1.0360 × 10^+03^	9.6022 × 10^+02^	8.7707 × 10^+02^	9.5195 × 10^+02^	8.3928 × 10^+02^	8.4660 × 10^+02^
	Mean	8.6635 × 10^+02^	9.4702 × 10^+02^	9.6566 × 10^+02^	9.0227 × 10^+02^	9.7913 × 10^+02^	9.0730 × 10^+02^	1.0892 × 10^+03^	1.0030 × 10^+03^	1.0001 × 10^+03^	9.8377 × 10^+02^	8.7167 × 10^+02^	8.9789 × 10^+02^
	Std	1.5456 × 10^+01^	2.4884 × 10^+01^	3.2542 × 10^+01^	2.5681 × 10^+01^	2.1225 × 10^+01^	2.2631 × 10^+01^	1.8007 × 10^+01^	1.9406 × 10^+01^	5.3153 × 10^+01^	1.5738 × 10^+01^	2.2934 × 10^+01^	2.2797 × 10^+01^
F8	Best	9.3393 × 10^+02^	1.6295 × 10^+03^	2.0607 × 10^+03^	1.3050 × 10^+03^	5.0586 × 10^+03^	1.6976 × 10^+03^	5.1397 × 10^+03^	2.5188 × 10^+03^	9.0018 × 10^+02^	9.0018 × 10^+02^	9.9743 × 10^+02^	9.7204 × 10^+02^
	Mean	1.1055 × 10^+03^	2.5268 × 10^+03^	3.8625 × 10^+03^	2.7142 × 10^+03^	7.6889 × 10^+03^	2.8052 × 10^+03^	8.1603 × 10^+03^	5.1014 × 10^+03^	1.5523 × 10^+03^	9.0739 × 10^+02^	2.0173 × 10^+03^	1.8981 × 10^+03^
	Std	1.1786 × 10^+02^	6.2672 × 10^+02^	8.0872 × 10^+02^	1.0763 × 10^+03^	1.5342 × 10^+03^	7.3351 × 10^+02^	2.0748 × 10^+03^	1.5057 × 10^+03^	1.2849 × 10^+03^	2.0814 × 10^+01^	8.1939 × 10^+02^	1.0395 × 10^+03^
F9	Best	3.3947 × 10^+03^	5.7506 × 10^+03^	4.5107 × 10^+03^	7.9020 × 10^+03^	5.1145 × 10^+03^	3.1836 × 10^+03^	6.6927 × 10^+03^	6.5241 × 10^+03^	6.5571 × 10^+03^	7.3562 × 10^+03^	5.5769 × 10^+03^	5.1790 × 10^+03^
	Mean	6.4138 × 10^+03^	6.7382 × 10^+03^	5.9804 × 10^+03^	8.4774 × 10^+03^	6.4791 × 10^+03^	4.5175 × 10^+03^	7.8480 × 10^+03^	7.3194 × 10^+03^	8.3161 × 10^+03^	8.2693 × 10^+03^	7.0864 × 10^+03^	6.8687 × 10^+03^
	Std	1.0742 × 10^+03^	5.2825 × 10^+02^	6.7791 × 10^+02^	2.5836 × 10^+02^	9.3647 × 10^+02^	6.2069 × 10^+02^	4.5965 × 10^+02^	3.7545 × 10^+02^	7.3960 × 10^+02^	3.8355 × 10^+02^	7.3888 × 10^+02^	8.5182 × 10^+02^
F10	Best	1.1431 × 10^+03^	1.3070 × 10^+03^	1.1595 × 10^+03^	1.1841 × 10^+03^	1.2883 × 10^+03^	1.1792 × 10^+03^	1.4727 × 10^+03^	1.2906 × 10^+03^	1.1774 × 10^+03^	1.1437 × 10^+03^	1.1512 × 10^+03^	1.1567 × 10^+03^
	Mean	1.2234 × 10^+03^	1.5088 × 10^+03^	1.2553 × 10^+03^	1.2515 × 10^+03^	1.7514 × 10^+03^	1.3326 × 10^+03^	1.8651 × 10^+03^	1.3875 × 10^+03^	1.7314 × 10^+03^	1.2187 × 10^+03^	1.2362 × 10^+03^	1.2594 × 10^+03^
	Std	4.5183 × 10^+01^	9.8685 × 10^+01^	4.3119 × 10^+01^	3.9539 × 10^+01^	4.3252 × 10^+02^	9.1741 × 10^+01^	4.8162 × 10^+02^	4.8357 × 10^+01^	1.2025 × 10^+03^	4.0266 × 10^+01^	5.3959 × 10^+01^	4.8726 × 10^+01^
F11	Best	8.4902 × 10^+04^	5.7533 × 10^+06^	1.3684 × 10^+06^	5.6045 × 10^+04^	6.1542 × 10^+05^	6.9727 × 10^+05^	6.9297 × 10^+07^	5.1001 × 10^+06^	3.4601 × 10^+05^	1.3960 × 10^+04^	3.7191 × 10^+04^	1.6762 × 10^+04^
	Mean	7.6286 × 10^+05^	4.3003 × 10^+07^	1.9315 × 10^+07^	3.2297 × 10^+05^	1.3746 × 10^+07^	4.2516 × 10^+06^	6.1490 × 10^+08^	1.6241 × 10^+07^	1.5362 × 10^+08^	2.5355 × 10^+05^	1.7262 × 10^+05^	1.8048 × 10^+05^
	Std	6.3699 × 10^+05^	3.3296 × 10^+07^	1.8308 × 10^+07^	2.7613 × 10^+05^	1.0863 × 10^+07^	3.1972 × 10^+06^	3.8783 × 10^+08^	9.1421 × 10^+06^	2.3969 × 10^+08^	1.9173 × 10^+05^	1.9039 × 10^+05^	1.4413 × 10^+05^
F12	Best	2.3551 × 10^+03^	1.4362 × 10^+04^	2.4018 × 10^+04^	2.4028 × 10^+03^	1.0163 × 10^+04^	3.2287 × 10^+03^	2.1974 × 10^+06^	3.1787 × 10^+04^	1.5905 × 10^+04^	5.3871 × 10^+03^	1.9875 × 10^+03^	3.4375 × 10^+03^
	Mean	4.3305 × 10^+03^	4.0614 × 10^+04^	7.1326 × 10^+04^	2.1890 × 10^+04^	4.0602 × 10^+05^	4.1078 × 10^+04^	7.1851 × 10^+06^	1.1588 × 10^+05^	4.2216 × 10^+06^	3.6997 × 10^+04^	9.4816 × 10^+03^	1.2018 × 10^+04^
	Std	1.5267 × 10^+03^	1.8748 × 10^+04^	3.4032 × 10^+04^	1.6874 × 10^+04^	1.1800 × 10^+06^	1.3987 × 10^+05^	3.8425 × 10^+06^	4.5875 × 10^+04^	6.4533 × 10^+06^	1.9466 × 10^+04^	8.8716 × 10^+03^	1.2389 × 10^+04^
F13	Best	1.4680 × 10^+03^	3.8312 × 10^+03^	2.3235 × 10^+03^	2.1479 × 10^+03^	1.2071 × 10^+04^	3.3927 × 10^+03^	1.8133 × 10^+03^	1.5472 × 10^+03^	1.6101 × 10^+03^	1.7812 × 10^+03^	1.4512 × 10^+03^	1.4740 × 10^+03^
	Mean	1.4926 × 10^+03^	2.3720 × 10^+04^	2.8819 × 10^+04^	2.6712 × 10^+04^	2.4965 × 10^+05^	8.5342 × 10^+04^	2.4137 × 10^+03^	1.5929 × 10^+03^	1.1885 × 10^+05^	4.0133 × 10^+03^	1.5371 × 10^+03^	1.5678 × 10^+03^
	Std	1.1165 × 10^+01^	2.4339 × 10^+04^	2.5682 × 10^+04^	2.8127 × 10^+04^	2.9340 × 10^+05^	1.0367 × 10^+05^	4.7711 × 10^+02^	2.4094 × 10^+01^	1.5112 × 10^+05^	2.6818 × 10^+03^	3.4783 × 10^+01^	7.1393 × 10^+01^
F14	Best	1.6607 × 10^+03^	9.2388 × 10^+03^	9.7013 × 10^+03^	1.8664 × 10^+03^	6.6626 × 10^+03^	1.9206 × 10^+03^	1.5061 × 10^+04^	2.9382 × 10^+03^	5.5882 × 10^+03^	2.8012 × 10^+03^	1.5962 × 10^+03^	1.6857 × 10^+03^
	Mean	1.7611 × 10^+03^	2.6898 × 10^+04^	3.3263 × 10^+04^	5.3867 × 10^+03^	4.2863 × 10^+04^	8.6773 × 10^+03^	8.0163 × 10^+04^	6.0902 × 10^+03^	2.6930 × 10^+05^	8.6771 × 10^+03^	2.0146 × 10^+03^	1.9598 × 10^+03^
	Std	6.2993 × 10^+01^	1.4000 × 10^+04^	2.1362 × 10^+04^	5.3916 × 10^+03^	3.7265 × 10^+04^	8.2636 × 10^+03^	4.3091 × 10^+04^	2.1319 × 10^+03^	7.6056 × 10^+05^	3.7980 × 10^+03^	6.2771 × 10^+02^	2.1688 × 10^+02^
F15	Best	1.7396 × 10^+03^	2.3501 × 10^+03^	2.3056 × 10^+03^	2.0180 × 10^+03^	2.0609 × 10^+03^	1.9843 × 10^+03^	3.3243 × 10^+03^	2.5941 × 10^+03^	3.1021 × 10^+03^	2.6986 × 10^+03^	1.6882 × 10^+03^	2.0519 × 10^+03^
	Mean	2.3233 × 10^+03^	2.7580 × 10^+03^	3.1467 × 10^+03^	2.6424 × 10^+03^	3.0987 × 10^+03^	2.7556 × 10^+03^	3.9947 × 10^+03^	3.1648 × 10^+03^	3.6720 × 10^+03^	3.1445 × 10^+03^	2.3928 × 10^+03^	2.5610 × 10^+03^
	Std	3.0155 × 10^+02^	1.9592 × 10^+02^	3.7222 × 10^+02^	4.0259 × 10^+02^	4.3866 × 10^+02^	3.6236 × 10^+02^	2.9152 × 10^+02^	2.7697 × 10^+02^	3.1632 × 10^+02^	2.4835 × 10^+02^	2.9967 × 10^+02^	2.9287 × 10^+02^
F16	Best	1.7303 × 10^+03^	1.8708 × 10^+03^	1.8460 × 10^+03^	1.7710 × 10^+03^	1.8936 × 10^+03^	1.7834 × 10^+03^	2.2954 × 10^+03^	2.0154 × 10^+03^	1.9587 × 10^+03^	1.9161 × 10^+03^	1.7881 × 10^+03^	1.7926 × 10^+03^
	Mean	1.8717 × 10^+03^	2.0489 × 10^+03^	2.3210 × 10^+03^	2.0522 × 10^+03^	2.3963 × 10^+03^	2.1573 × 10^+03^	2.6155 × 10^+03^	2.2382 × 10^+03^	2.4966 × 10^+03^	2.0492 × 10^+03^	1.9237 × 10^+03^	1.9815 × 10^+03^
	Std	1.1887 × 10^+02^	1.0910 × 10^+02^	2.0316 × 10^+02^	1.8533 × 10^+02^	2.3767 × 10^+02^	2.0930 × 10^+02^	1.9915 × 10^+02^	1.0458 × 10^+02^	2.8163 × 10^+02^	1.0614 × 10^+02^	9.2886 × 10^+01^	1.2951 × 10^+02^
F17	Best	1.9360 × 10^+03^	4.6067 × 10^+04^	5.5392 × 10^+04^	1.2063 × 10^+05^	3.5723 × 10^+05^	7.7787 × 10^+04^	3.2641 × 10^+04^	5.6644 × 10^+03^	6.2320 × 10^+04^	2.2437 × 10^+05^	4.5962 × 10^+03^	3.2559 × 10^+03^
	Mean	2.0885 × 10^+03^	2.6370 × 10^+05^	3.0759 × 10^+05^	7.3022 × 10^+05^	2.6680 × 10^+06^	4.7564 × 10^+05^	1.2456 × 10^+05^	1.3352 × 10^+04^	5.3380 × 10^+06^	6.6710 × 10^+05^	2.4456 × 10^+04^	2.2381 × 10^+04^
	Std	1.0899 × 10^+02^	3.3854 × 10^+05^	2.1807 × 10^+05^	5.8117 × 10^+05^	3.6017 × 10^+06^	3.8868 × 10^+05^	6.4756 × 10^+04^	6.5980 × 10^+03^	4.9881 × 10^+06^	3.1213 × 10^+05^	1.8579 × 10^+04^	1.2542 × 10^+04^
F18	Best	1.9467 × 10^+03^	6.2498 × 10^+03^	4.4898 × 10^+04^	1.9655 × 10^+03^	4.3423 × 10^+03^	1.9706 × 10^+03^	3.1763 × 10^+04^	2.6829 × 10^+03^	2.5329 × 10^+03^	2.1920 × 10^+03^	1.9708 × 10^+03^	1.9743 × 10^+03^
	Mean	1.9835 × 10^+03^	2.0144 × 10^+05^	4.5064 × 10^+05^	6.8182 × 10^+03^	3.6752 × 10^+04^	6.8709 × 10^+03^	3.2969 × 10^+05^	6.2404 × 10^+03^	4.6703 × 10^+05^	9.0507 × 10^+03^	2.0871 × 10^+03^	2.0942 × 10^+03^
	Std	2.5384 × 10^+01^	3.7344 × 10^+05^	2.8761 × 10^+05^	7.4415 × 10^+03^	4.5282 × 10^+04^	4.6824 × 10^+03^	3.1590 × 10^+05^	2.9218 × 10^+03^	1.2905 × 10^+06^	7.4908 × 10^+03^	1.7872 × 10^+02^	7.1224 × 10^+01^
F19	Best	2.0915 × 10^+03^	2.1868 × 10^+03^	2.2818 × 10^+03^	2.1304 × 10^+03^	2.2851 × 10^+03^	2.2029 × 10^+03^	2.6672 × 10^+03^	2.4414 × 10^+03^	2.2788 × 10^+03^	2.2737 × 10^+03^	2.1910 × 10^+03^	2.1396 × 10^+03^
	Mean	2.2603 × 10^+03^	2.5013 × 10^+03^	2.4640 × 10^+03^	2.4317 × 10^+03^	2.7104 × 10^+03^	2.5047 × 10^+03^	2.9552 × 10^+03^	2.6508 × 10^+03^	2.7178 × 10^+03^	2.5455 × 10^+03^	2.3787 × 10^+03^	2.3119 × 10^+03^
	Std	1.0559 × 10^+02^	1.4174 × 10^+02^	1.2759 × 10^+02^	1.6650 × 10^+02^	2.2117 × 10^+02^	1.6900 × 10^+02^	1.4707 × 10^+02^	1.0718 × 10^+02^	1.9717 × 10^+02^	1.3032 × 10^+02^	1.3989 × 10^+02^	9.8594 × 10^+01^
F20	Best	2.3389 × 10^+03^	2.4199 × 10^+03^	2.4216 × 10^+03^	2.3681 × 10^+03^	2.3819 × 10^+03^	2.3533 × 10^+03^	2.5310 × 10^+03^	2.4268 × 10^+03^	2.3610 × 10^+03^	2.4540 × 10^+03^	2.3380 × 10^+03^	2.3443 × 10^+03^
	Mean	2.3639 × 10^+03^	2.4511 × 10^+03^	2.4861 × 10^+03^	2.4057 × 10^+03^	2.4714 × 10^+03^	2.3963 × 10^+03^	2.5959 × 10^+03^	2.5033 × 10^+03^	2.4937 × 10^+03^	2.4827 × 10^+03^	2.3720 × 10^+03^	2.3855 × 10^+03^
	Std	1.2618 × 10^+01^	1.6492 × 10^+01^	3.1373 × 10^+01^	2.8460 × 10^+01^	3.9635 × 10^+01^	2.3250 × 10^+01^	2.6645 × 10^+01^	2.2700 × 10^+01^	4.6980 × 10^+01^	1.5760 × 10^+01^	1.7406 × 10^+01^	2.3938 × 10^+01^
F21	Best	2.3014 × 10^+03^	2.4063 × 10^+03^	2.3314 × 10^+03^	2.3002 × 10^+03^	2.3226 × 10^+03^	2.3219 × 10^+03^	4.6231 × 10^+03^	3.7389 × 10^+03^	4.6341 × 10^+03^	2.3000 × 10^+03^	2.3003 × 10^+03^	2.3003 × 10^+03^
	Mean	2.3066 × 10^+03^	2.9747 × 10^+03^	2.3792 × 10^+03^	2.5733 × 10^+03^	3.5038 × 10^+03^	2.3443 × 10^+03^	6.7144 × 10^+03^	8.0698 × 10^+03^	9.1984 × 10^+03^	2.5595 × 10^+03^	2.5490 × 10^+03^	3.5939 × 10^+03^
	Std	8.1372 × 10^+00^	1.3863 × 10^+03^	4.1358 × 10^+01^	1.4020 × 10^+03^	1.7158 × 10^+03^	2.0386 × 10^+01^	1.5100 × 10^+03^	1.8077 × 10^+03^	1.4495 × 10^+03^	1.4171 × 10^+03^	1.0712 × 10^+03^	2.3715 × 10^+03^
F22	Best	2.6895 × 10^+03^	2.7723 × 10^+03^	2.8262 × 10^+03^	2.7249 × 10^+03^	2.7589 × 10^+03^	2.7350 × 10^+03^	2.9685 × 10^+03^	2.7970 × 10^+03^	2.7176 × 10^+03^	2.7720 × 10^+03^	2.7033 × 10^+03^	2.6847 × 10^+03^
	Mean	2.7227 × 10^+03^	2.8213 × 10^+03^	2.9137 × 10^+03^	2.7865 × 10^+03^	2.8575 × 10^+03^	2.7869 × 10^+03^	3.1176 × 10^+03^	2.8881 × 10^+03^	2.8625 × 10^+03^	2.8332 × 10^+03^	2.7509 × 10^+03^	2.7689 × 10^+03^
	Std	1.9340 × 10^+01^	2.6658 × 10^+01^	5.7286 × 10^+01^	3.3059 × 10^+01^	8.1439 × 10^+01^	3.4859 × 10^+01^	6.4662 × 10^+01^	2.9062 × 10^+01^	5.5111 × 10^+01^	2.3347 × 10^+01^	2.7614 × 10^+01^	4.6887 × 10^+01^
F23	Best	2.8510 × 10^+03^	2.8981 × 10^+03^	2.9506 × 10^+03^	2.9006 × 10^+03^	2.9392 × 10^+03^	2.9041 × 10^+03^	3.0766 × 10^+03^	3.0054 × 10^+03^	2.9886 × 10^+03^	2.9507 × 10^+03^	2.8635 × 10^+03^	2.8771 × 10^+03^
	Mean	2.8848 × 10^+03^	2.9677 × 10^+03^	3.0561 × 10^+03^	2.9575 × 10^+03^	3.0314 × 10^+03^	2.9682 × 10^+03^	3.2600 × 10^+03^	3.0511 × 10^+03^	3.0741 × 10^+03^	3.0029 × 10^+03^	2.9056 × 10^+03^	2.9176 × 10^+03^
	Std	1.6540 × 10^+01^	3.4857 × 10^+01^	5.2991 × 10^+01^	3.9256 × 10^+01^	7.0020 × 10^+01^	4.5134 × 10^+01^	7.5357 × 10^+01^	2.5133 × 10^+01^	5.4734 × 10^+01^	1.6645 × 10^+01^	2.9134 × 10^+01^	3.1260 × 10^+01^
F24	Best	2.8843 × 10^+03^	2.9761 × 10^+03^	2.9249 × 10^+03^	2.8851 × 10^+03^	2.9164 × 10^+03^	2.9101 × 10^+03^	3.2450 × 10^+03^	2.9221 × 10^+03^	2.8796 × 10^+03^	2.8876 × 10^+03^	2.8843 × 10^+03^	2.8840 × 10^+03^
	Mean	2.9136 × 10^+03^	3.0330 × 10^+03^	2.9879 × 10^+03^	2.9183 × 10^+03^	2.9819 × 10^+03^	2.9585 × 10^+03^	3.5453 × 10^+03^	3.0022 × 10^+03^	2.9108 × 10^+03^	2.9084 × 10^+03^	2.9019 × 10^+03^	2.8994 × 10^+03^
	Std	2.2238 × 10^+01^	2.9030 × 10^+01^	2.9832 × 10^+01^	2.2769 × 10^+01^	3.7876 × 10^+01^	2.8313 × 10^+01^	1.9462 × 10^+02^	2.9217 × 10^+01^	9.2549 × 10^+01^	1.9444 × 10^+01^	1.8301 × 10^+01^	1.9730 × 10^+01^
F25	Best	2.8061 × 10^+03^	3.3334 × 10^+03^	3.0023 × 10^+03^	2.8034 × 10^+03^	3.4798 × 10^+03^	2.9739 × 10^+03^	6.8843 × 10^+03^	4.8722 × 10^+03^	5.0805 × 10^+03^	4.8336 × 10^+03^	2.8043 × 10^+03^	2.8029 × 10^+03^
	Mean	4.2168 × 10^+03^	5.4441 × 10^+03^	4.4066 × 10^+03^	5.1056 × 10^+03^	6.3407 × 10^+03^	5.0520 × 10^+03^	7.5623 × 10^+03^	5.9822 × 10^+03^	5.6756 × 10^+03^	5.4489 × 10^+03^	4.5573 × 10^+03^	4.4560 × 10^+03^
	Std	6.8729 × 10^+02^	1.2019 × 10^+03^	1.7594 × 10^+03^	1.1704 × 10^+03^	1.6968 × 10^+03^	1.1946 × 10^+03^	3.8967 × 10^+02^	4.1542 × 10^+02^	3.7174 × 10^+02^	2.9863 × 10^+02^	6.3363 × 10^+02^	7.2146 × 10^+02^
F26	Best	3.1937 × 10^+03^	3.2308 × 10^+03^	3.2340 × 10^+03^	3.2140 × 10^+03^	3.2223 × 10^+03^	3.2375 × 10^+03^	3.2778 × 10^+03^	3.2356 × 10^+03^	3.2000 × 10^+03^	3.1994 × 10^+03^	3.1944 × 10^+03^	3.2102 × 10^+03^
	Mean	3.2220 × 10^+03^	3.2862 × 10^+03^	3.3310 × 10^+03^	3.2601 × 10^+03^	3.2811 × 10^+03^	3.2795 × 10^+03^	3.3625 × 10^+03^	3.2896 × 10^+03^	3.2000 × 10^+03^	3.2252 × 10^+03^	3.2357 × 10^+03^	3.2288 × 10^+03^
	Std	1.5542 × 10^+01^	2.1792 × 10^+01^	6.0358 × 10^+01^	2.8944 × 10^+01^	3.7619 × 10^+01^	3.2600 × 10^+01^	6.6158 × 10^+01^	2.6298 × 10^+01^	1.7590 × 10^−04^	1.5503 × 10^+01^	2.1247 × 10^+01^	1.3300 × 10^+01^
F27	Best	3.2118 × 10^+03^	3.3481 × 10^+03^	3.2476 × 10^+03^	3.2022 × 10^+03^	3.2749 × 10^+03^	3.2881 × 10^+03^	3.7219 × 10^+03^	3.3240 × 10^+03^	3.2890 × 10^+03^	3.2049 × 10^+03^	3.1922 × 10^+03^	3.2057 × 10^+03^
	Mean	3.2528 × 10^+03^	3.4293 × 10^+03^	3.3320 × 10^+03^	3.2454 × 10^+03^	3.3708 × 10^+03^	3.3383 × 10^+03^	4.3751 × 10^+03^	3.4246 × 10^+03^	3.2996 × 10^+03^	3.2454 × 10^+03^	3.2430 × 10^+03^	3.2520 × 10^+03^
	Std	2.8228 × 10^+01^	5.8194 × 10^+01^	3.5237 × 10^+01^	2.6228 × 10^+01^	8.5635 × 10^+01^	3.0669 × 10^+01^	3.9221 × 10^+02^	5.0466 × 10^+01^	2.0441 × 10^+00^	2.2929 × 10^+01^	4.2838 × 10^+01^	2.9152 × 10^+01^
F28	Best	3.4598 × 10^+03^	3.6988 × 10^+03^	4.1768 × 10^+03^	3.5869 × 10^+03^	3.5910 × 10^+03^	3.5392 × 10^+03^	4.2541 × 10^+03^	3.9330 × 10^+03^	3.4920 × 10^+03^	3.6556 × 10^+03^	3.4880 × 10^+03^	3.6033 × 10^+03^
	Mean	3.6140 × 10^+03^	4.0996 × 10^+03^	4.6572 × 10^+03^	3.8822 × 10^+03^	4.2510 × 10^+03^	3.9085 × 10^+03^	4.8103 × 10^+03^	4.2699 × 10^+03^	4.3863 × 10^+03^	3.8952 × 10^+03^	3.7115 × 10^+03^	3.8140 × 10^+03^
	Std	1.1361 × 10^+02^	1.9016 × 10^+02^	2.9702 × 10^+02^	1.8108 × 10^+02^	2.8338 × 10^+02^	2.1868 × 10^+02^	2.9526 × 10^+02^	1.8748 × 10^+02^	4.0386 × 10^+02^	1.0710 × 10^+02^	1.4026 × 10^+02^	1.6936 × 10^+02^
F29	Best	7.3335 × 10^+03^	1.4368 × 10^+05^	7.4228 × 10^+05^	5.7883 × 10^+03^	1.2266 × 10^+05^	7.9655 × 10^+03^	1.0559 × 10^+06^	1.5008 × 10^+05^	3.3502 × 10^+03^	2.0112 × 10^+04^	6.0771 × 10^+03^	6.4711 × 10^+03^
	Mean	1.6414 × 10^+04^	2.1958 × 10^+06^	3.9694 × 10^+06^	1.3890 × 10^+04^	9.2527 × 10^+05^	5.7236 × 10^+04^	5.9794 × 10^+06^	4.0874 × 10^+05^	6.7222 × 10^+05^	5.5759 × 10^+04^	1.1911 × 10^+04^	1.3115 × 10^+04^
	Std	7.3173 × 10^+03^	2.0462 × 10^+06^	1.7817 × 10^+06^	1.2785 × 10^+04^	1.0244 × 10^+06^	4.2130 × 10^+04^	3.6983 × 10^+06^	2.6934 × 10^+05^	1.8466 × 10^+06^	3.3325 × 10^+04^	4.0532 × 10^+03^	5.1206 × 10^+03^

**Table A3:** Comparison of results on CEC 2017 (D = 50).

Function	Metric	MICFOA	CFOA	PEOA	TLBO	COA	ARO	EDO	YDSE	LSHADE	JADE	IDE-EDA	APSM-JSO
F1	Best	1.0012 × 10^+07^	4.7809 × 10^+09^	5.6454 × 10^+05^	2.9161 × 10^+07^	3.7990 × 10^+09^	4.5254 × 10^+08^	5.7358 × 10^+10^	7.1090 × 10^+09^	1.1268 × 10^+05^	6.4369 × 10^+05^	3.2493 × 10^+07^	1.1784 × 10^+07^
	Mean	3.4421 × 10^+07^	7.9460 × 10^+09^	4.5711 × 10^+06^	6.8967 × 10^+08^	1.1290 × 10^+10^	2.7576 × 10^+09^	7.7669 × 10^+10^	1.3573 × 10^+10^	2.5570 × 10^+09^	5.5285 × 10^+07^	1.2763 × 10^+08^	1.0216 × 10^+08^
	Std	1.7556 × 10^+07^	1.9394 × 10^+09^	3.7388 × 10^+06^	1.0876 × 10^+09^	5.5628 × 10^+09^	1.4067 × 10^+09^	1.0758 × 10^+10^	3.3661 × 10^+09^	5.9900 × 10^+09^	6.6480 × 10^+07^	1.2892 × 10^+08^	7.9338 × 10^+07^
F2	Best	3.2259 × 10^+04^	1.0839 × 10^+05^	2.8190 × 10^+04^	1.2421 × 10^+05^	2.0703 × 10^+05^	1.2771 × 10^+05^	8.8829 × 10^+04^	7.4970 × 10^+04^	1.3181 × 10^+05^	1.5218 × 10^+05^	3.6068 × 10^+04^	3.0422 × 10^+04^
	Mean	5.5096 × 10^+04^	1.5390 × 10^+05^	4.2233 × 10^+04^	1.9118 × 10^+05^	3.3348 × 10^+05^	1.6801 × 10^+05^	1.3421 × 10^+05^	1.0738 × 10^+05^	4.8712 × 10^+05^	1.9790 × 10^+05^	6.5137 × 10^+04^	6.9491 × 10^+04^
	Std	1.3720 × 10^+04^	2.1369 × 10^+04^	8.4401 × 10^+03^	3.0467 × 10^+04^	6.4619 × 10^+04^	2.1664 × 10^+04^	2.0337 × 10^+04^	2.1067 × 10^+04^	1.5148 × 10^+05^	3.1885 × 10^+04^	1.6624 × 10^+04^	1.9860 × 10^+04^
F3	Best	5.6825 × 10^+02^	1.1765 × 10^+03^	5.9018 × 10^+02^	5.1875 × 10^+02^	7.6211 × 10^+02^	7.4917 × 10^+02^	9.3859 × 10^+03^	1.1860 × 10^+03^	4.4804 × 10^+02^	4.9833 × 10^+02^	5.5213 × 10^+02^	5.2143 × 10^+02^
	Mean	6.6359 × 10^+02^	1.7706 × 10^+03^	6.8353 × 10^+02^	6.7847 × 10^+02^	1.5224 × 10^+03^	1.0945 × 10^+03^	1.7797 × 10^+04^	1.8795 × 10^+03^	1.2729 × 10^+03^	6.6171 × 10^+02^	6.6141 × 10^+02^	6.4408 × 10^+02^
	Std	6.3869 × 10^+01^	4.1022 × 10^+02^	5.6239 × 10^+01^	8.2079 × 10^+01^	6.1819 × 10^+02^	1.9856 × 10^+02^	4.3725 × 10^+03^	4.2332 × 10^+02^	1.7838 × 10^+03^	5.9186 × 10^+01^	5.7956 × 10^+01^	4.7211 × 10^+01^
F4	Best	6.3856 × 10^+02^	7.9339 × 10^+02^	8.2039 × 10^+02^	6.9254 × 10^+02^	8.5409 × 10^+02^	7.3529 × 10^+02^	1.0333 × 10^+03^	9.3408 × 10^+02^	6.5865 × 10^+02^	8.6500 × 10^+02^	6.4102 × 10^+02^	6.3784 × 10^+02^
	Mean	6.9725 × 10^+02^	8.8663 × 10^+02^	9.0299 × 10^+02^	7.6201 × 10^+02^	8.9871 × 10^+02^	8.0652 × 10^+02^	1.1065 × 10^+03^	9.8605 × 10^+02^	9.3943 × 10^+02^	9.0459 × 10^+02^	7.1647 × 10^+02^	7.4462 × 10^+02^
	Std	3.0209 × 10^+01^	4.7921 × 10^+01^	4.1141 × 10^+01^	4.7197 × 10^+01^	2.0225 × 10^+01^	4.5535 × 10^+01^	3.5928 × 10^+01^	2.6173 × 10^+01^	1.3092 × 10^+02^	2.1152 × 10^+01^	3.9731 × 10^+01^	5.5002 × 10^+01^
F5	Best	6.1259 × 10^+02^	6.3676 × 10^+02^	6.5532 × 10^+02^	6.2026 × 10^+02^	6.6001 × 10^+02^	6.1590 × 10^+02^	6.6956 × 10^+02^	6.5397 × 10^+02^	6.0003 × 10^+02^	6.0025 × 10^+02^	6.1277 × 10^+02^	6.0950 × 10^+02^
	Mean	6.1831 × 10^+02^	6.5256 × 10^+02^	6.7027 × 10^+02^	6.3345 × 10^+02^	6.6828 × 10^+02^	6.3127 × 10^+02^	6.9240 × 10^+02^	6.6727 × 10^+02^	6.1523 × 10^+02^	6.0164 × 10^+02^	6.2376 × 10^+02^	6.2042 × 10^+02^
	Std	3.9764 × 10^+00^	7.4398 × 10^+00^	7.6395 × 10^+00^	7.8487 × 10^+00^	5.7364 × 10^+00^	9.9696 × 10^+00^	9.4656 × 10^+00^	6.8676 × 10^+00^	1.9337 × 10^+01^	1.2840 × 10^+00^	7.6209 × 10^+00^	6.3084 × 10^+00^
F6	Best	9.8078 × 10^+02^	1.2606 × 10^+03^	1.3160 × 10^+03^	1.1347 × 10^+03^	1.4291 × 10^+03^	1.1642 × 10^+03^	1.5999 × 10^+03^	1.3444 × 10^+03^	9.9179 × 10^+02^	1.0987 × 10^+03^	9.8915 × 10^+02^	1.0410 × 10^+03^
	Mean	1.0918 × 10^+03^	1.3968 × 10^+03^	1.4795 × 10^+03^	1.2463 × 10^+03^	1.7225 × 10^+03^	1.3229 × 10^+03^	1.8541 × 10^+03^	1.4925 × 10^+03^	1.3289 × 10^+03^	1.1725 × 10^+03^	1.1774 × 10^+03^	1.1982 × 10^+03^
	Std	6.9510 × 10^+01^	7.1216 × 10^+01^	1.1202 × 10^+02^	8.3077 × 10^+01^	1.0685 × 10^+02^	8.6772 × 10^+01^	1.0107 × 10^+02^	7.7648 × 10^+01^	4.0952 × 10^+02^	2.9944 × 10^+01^	1.0222 × 10^+02^	1.0285 × 10^+02^
F7	Best	9.2892 × 10^+02^	1.0673 × 10^+03^	1.1387 × 10^+03^	9.7709 × 10^+02^	1.1834 × 10^+03^	9.7245 × 10^+02^	1.3606 × 10^+03^	1.2139 × 10^+03^	9.1865 × 10^+02^	1.1573 × 10^+03^	9.3950 × 10^+02^	9.6470 × 10^+02^
	Mean	9.9177 × 10^+02^	1.1908 × 10^+03^	1.2367 × 10^+03^	1.0572 × 10^+03^	1.2403 × 10^+03^	1.0887 × 10^+03^	1.4197 × 10^+03^	1.2725 × 10^+03^	1.2009 × 10^+03^	1.2039 × 10^+03^	1.0194 × 10^+03^	1.0694 × 10^+03^
	Std	3.8060 × 10^+01^	4.2277 × 10^+01^	4.2728 × 10^+01^	3.9721 × 10^+01^	3.0109 × 10^+01^	4.3870 × 10^+01^	2.8976 × 10^+01^	2.7137 × 10^+01^	1.2299 × 10^+02^	2.2552 × 10^+01^	3.6904 × 10^+01^	5.4131 × 10^+01^
F8	Best	1.7694 × 10^+03^	6.6691 × 10^+03^	8.0787 × 10^+03^	5.9094 × 10^+03^	1.9938 × 10^+04^	6.4295 × 10^+03^	2.6848 × 10^+04^	1.4709 × 10^+04^	9.6090 × 10^+02^	9.4273 × 10^+02^	6.5292 × 10^+03^	4.7706 × 10^+03^
	Mean	5.2280 × 10^+03^	1.2833 × 10^+04^	1.2353 × 10^+04^	1.4294 × 10^+04^	2.8023 × 10^+04^	1.1108 × 10^+04^	3.4059 × 10^+04^	2.2488 × 10^+04^	1.3605 × 10^+04^	1.9416 × 10^+03^	1.2570 × 10^+04^	1.1639 × 10^+04^
	Std	3.2041 × 10^+03^	3.2504 × 10^+03^	3.0696 × 10^+03^	3.7072 × 10^+03^	4.4214 × 10^+03^	2.4100 × 10^+03^	4.0593 × 10^+03^	4.4651 × 10^+03^	1.7211 × 10^+04^	7.6491 × 10^+02^	4.5480 × 10^+03^	4.8453 × 10^+03^
F9	Best	6.2859 × 10^+03^	9.8923 × 10^+03^	9.2355 × 10^+03^	1.3182 × 10^+04^	1.2091 × 10^+04^	6.1781 × 10^+03^	1.2066 × 10^+04^	1.2667 × 10^+04^	9.4618 × 10^+03^	1.3275 × 10^+04^	9.6832 × 10^+03^	1.0031 × 10^+04^
	Mean	1.2647 × 10^+04^	1.2107 × 10^+04^	1.0961 × 10^+04^	1.4841 × 10^+04^	1.3738 × 10^+04^	8.1449 × 10^+03^	1.4455 × 10^+04^	1.3785 × 10^+04^	1.4481 × 10^+04^	1.4707 × 10^+04^	1.2751 × 10^+04^	1.2665 × 10^+04^
	Std	1.3959 × 10^+03^	1.0057 × 10^+03^	9.9232 × 10^+02^	5.6817 × 10^+02^	7.8429 × 10^+02^	9.7915 × 10^+02^	1.0715 × 10^+03^	5.6633 × 10^+02^	1.7241 × 10^+03^	6.6982 × 10^+02^	1.3446 × 10^+03^	1.0175 × 10^+03^
F10	Best	1.3048 × 10^+03^	3.2146 × 10^+03^	1.3423 × 10^+03^	1.2841 × 10^+03^	2.3729 × 10^+03^	1.5405 × 10^+03^	3.5973 × 10^+03^	2.0804 × 10^+03^	1.6867 × 10^+03^	1.2849 × 10^+03^	1.2782 × 10^+03^	1.2786 × 10^+03^
	Mean	1.4529 × 10^+03^	6.0020 × 10^+03^	1.4916 × 10^+03^	1.4272 × 10^+03^	6.3684 × 10^+03^	2.4080 × 10^+03^	7.4859 × 10^+03^	3.0576 × 10^+03^	1.8968 × 10^+04^	1.4381 × 10^+03^	1.4419 × 10^+03^	1.4691 × 10^+03^
	Std	9.2930 × 10^+01^	1.6899 × 10^+03^	7.8585 × 10^+01^	6.6945 × 10^+01^	2.3848 × 10^+03^	6.5575 × 10^+02^	2.7643 × 10^+03^	7.1068 × 10^+02^	2.0445 × 10^+04^	6.7140 × 10^+01^	1.5032 × 10^+02^	9.8788 × 10^+01^
F11	Best	2.2525 × 10^+06^	2.0195 × 10^+08^	8.5289 × 10^+06^	5.8370 × 10^+05^	4.3902 × 10^+07^	2.4298 × 10^+07^	6.5437 × 10^+09^	2.6483 × 10^+08^	2.3619 × 10^+06^	3.5267 × 10^+05^	1.4676 × 10^+06^	3.9099 × 10^+05^
	Mean	9.8815 × 10^+06^	5.0047 × 10^+08^	1.0019 × 10^+08^	1.0194 × 10^+07^	6.6528 × 10^+08^	8.5452 × 10^+07^	1.7015 × 10^+10^	6.2240 × 10^+08^	1.8253 × 10^+09^	2.4702 × 10^+06^	7.5252 × 10^+06^	8.0318 × 10^+06^
	Std	5.1386 × 10^+06^	2.6773 × 10^+08^	6.5887 × 10^+07^	1.0320 × 10^+07^	1.3691 × 10^+09^	4.2392 × 10^+07^	9.5084 × 10^+09^	2.1898 × 10^+08^	2.4255 × 10^+09^	1.3165 × 10^+06^	6.5148 × 10^+06^	6.4418 × 10^+06^
F12	Best	3.4215 × 10^+03^	7.0073 × 10^+04^	1.9373 × 10^+04^	5.3728 × 10^+03^	2.3320 × 10^+05^	2.4731 × 10^+04^	2.1336 × 10^+08^	4.4113 × 10^+06^	5.2031 × 10^+03^	5.2288 × 10^+03^	5.2311 × 10^+03^	5.0572 × 10^+03^
	Mean	1.1265 × 10^+04^	8.2332 × 10^+05^	7.4306 × 10^+04^	1.3492 × 10^+04^	3.3127 × 10^+06^	2.5057 × 10^+05^	6.3460 × 10^+08^	1.2732 × 10^+07^	1.1891 × 10^+08^	1.2500 × 10^+04^	1.3231 × 10^+04^	1.7016 × 10^+04^
	Std	8.2396 × 10^+03^	7.2675 × 10^+05^	3.4250 × 10^+04^	8.9675 × 10^+03^	6.0746 × 10^+06^	1.9484 × 10^+05^	4.7933 × 10^+08^	6.9738 × 10^+06^	3.0421 × 10^+08^	8.3055 × 10^+03^	8.4412 × 10^+03^	9.7655 × 10^+03^
F13	Best	1.6344 × 10^+03^	3.0217 × 10^+04^	1.0991 × 10^+04^	3.0393 × 10^+04^	2.0906 × 10^+05^	5.3545 × 10^+04^	2.4117 × 10^+04^	2.5645 × 10^+03^	1.4093 × 10^+04^	3.2963 × 10^+04^	1.8841 × 10^+03^	1.8657 × 10^+03^
	Mean	1.7104 × 10^+03^	1.5822 × 10^+05^	2.6236 × 10^+05^	1.8384 × 10^+05^	1.6781 × 10^+06^	9.1057 × 10^+05^	1.8353 × 10^+05^	5.3430 × 10^+03^	3.7218 × 10^+06^	1.8523 × 10^+05^	1.9615 × 10^+04^	8.2163 × 10^+03^
	Std	3.8239 × 10^+01^	1.1883 × 10^+05^	1.7073 × 10^+05^	1.2339 × 10^+05^	2.1552 × 10^+06^	1.0998 × 10^+06^	1.2576 × 10^+05^	2.2607 × 10^+03^	4.1708 × 10^+06^	1.3222 × 10^+05^	2.5590 × 10^+04^	1.3176 × 10^+04^
F14	Best	2.6817 × 10^+03^	5.9716 × 10^+03^	1.5390 × 10^+04^	1.8987 × 10^+03^	3.4784 × 10^+04^	3.1713 × 10^+03^	3.5908 × 10^+06^	9.3901 × 10^+04^	9.4889 × 10^+03^	4.7204 × 10^+03^	2.1693 × 10^+03^	2.4873 × 10^+03^
	Mean	3.8501 × 10^+03^	2.0443 × 10^+04^	3.9412 × 10^+04^	1.1741 × 10^+04^	6.1668 × 10^+05^	1.3257 × 10^+04^	1.9574 × 10^+07^	2.8161 × 10^+05^	2.2913 × 10^+07^	1.6023 × 10^+04^	6.6847 × 10^+03^	9.3104 × 10^+03^
	Std	2.3627 × 10^+03^	1.0635 × 10^+04^	2.2761 × 10^+04^	6.9608 × 10^+03^	2.2423 × 10^+06^	7.1980 × 10^+03^	1.0586 × 10^+07^	1.6335 × 10^+05^	7.2615 × 10^+07^	1.0310 × 10^+04^	4.1439 × 10^+03^	6.1552 × 10^+03^
F15	Best	2.0731 × 10^+03^	2.7844 × 10^+03^	3.2080 × 10^+03^	2.1168 × 10^+03^	2.8628 × 10^+03^	2.7072 × 10^+03^	5.4929 × 10^+03^	3.8654 × 10^+03^	4.6945 × 10^+03^	3.2978 × 10^+03^	2.1562 × 10^+03^	2.5646 × 10^+03^
	Mean	2.8336 × 10^+03^	3.5747 × 10^+03^	4.4737 × 10^+03^	3.3136 × 10^+03^	4.2003 × 10^+03^	3.4733 × 10^+03^	6.1693 × 10^+03^	4.6278 × 10^+03^	5.6181 × 10^+03^	4.6164 × 10^+03^	2.9784 × 10^+03^	3.3355 × 10^+03^
	Std	3.1451 × 10^+02^	4.1362 × 10^+02^	6.0818 × 10^+02^	5.2280 × 10^+02^	6.3211 × 10^+02^	4.2271 × 10^+02^	3.7462 × 10^+02^	3.0304 × 10^+02^	5.3352 × 10^+02^	3.5263 × 10^+02^	4.4091 × 10^+02^	4.3839 × 10^+02^
F16	Best	2.1345 × 10^+03^	2.7596 × 10^+03^	2.6631 × 10^+03^	2.3526 × 10^+03^	3.0216 × 10^+03^	2.5816 × 10^+03^	3.7759 × 10^+03^	3.2392 × 10^+03^	2.9431 × 10^+03^	3.2936 × 10^+03^	2.2027 × 10^+03^	2.2498 × 10^+03^
	Mean	2.7115 × 10^+03^	3.1561 × 10^+03^	3.6254 × 10^+03^	3.0291 × 10^+03^	3.6380 × 10^+03^	3.1161 × 10^+03^	4.4424 × 10^+03^	3.6779 × 10^+03^	4.4550 × 10^+03^	3.7577 × 10^+03^	2.9479 × 10^+03^	3.0510 × 10^+03^
	Std	3.4375 × 10^+02^	2.2788 × 10^+02^	3.7411 × 10^+02^	3.2323 × 10^+02^	3.1517 × 10^+02^	2.6928 × 10^+02^	2.9130 × 10^+02^	2.5470 × 10^+02^	6.8130 × 10^+02^	2.0782 × 10^+02^	3.2459 × 10^+02^	2.9240 × 10^+02^
F17	Best	8.3192 × 10^+03^	2.7957 × 10^+05^	3.3258 × 10^+05^	5.3379 × 10^+05^	9.7698 × 10^+05^	6.2069 × 10^+05^	4.6286 × 10^+05^	4.6692 × 10^+04^	5.4002 × 10^+05^	1.2739 × 10^+06^	2.6530 × 10^+04^	2.8906 × 10^+04^
	Mean	3.5535 × 10^+04^	1.9544 × 10^+06^	2.1809 × 10^+06^	2.3624 × 10^+06^	7.7120 × 10^+06^	2.9535 × 10^+06^	1.9586 × 10^+06^	1.8418 × 10^+05^	2.3520 × 10^+07^	3.4158 × 10^+06^	1.9820 × 10^+05^	1.6088 × 10^+05^
	Std	2.1678 × 10^+04^	1.5998 × 10^+06^	1.3448 × 10^+06^	2.5825 × 10^+06^	8.3977 × 10^+06^	2.8456 × 10^+06^	2.0965 × 10^+06^	1.1043 × 10^+05^	1.8083 × 10^+07^	1.4008 × 10^+06^	1.4064 × 10^+05^	1.8021 × 10^+05^
F18	Best	2.2389 × 10^+03^	2.5939 × 10^+04^	1.6450 × 10^+05^	2.3599 × 10^+03^	5.8543 × 10^+04^	3.5911 × 10^+03^	2.4964 × 10^+06^	8.5186 × 10^+04^	3.3344 × 10^+03^	7.4763 × 10^+03^	2.1526 × 10^+03^	2.1274 × 10^+03^
	Mean	6.3014 × 10^+03^	3.4323 × 10^+05^	1.5220 × 10^+06^	1.7577 × 10^+04^	3.4448 × 10^+05^	1.8329 × 10^+04^	1.4930 × 10^+07^	3.6202 × 10^+05^	8.1351 × 10^+06^	2.2899 × 10^+04^	1.5856 × 10^+04^	1.2408 × 10^+04^
	Std	4.9914 × 10^+03^	4.3921 × 10^+05^	9.1700 × 10^+05^	8.6447 × 10^+03^	4.1303 × 10^+05^	1.0650 × 10^+04^	1.1377 × 10^+07^	1.7532 × 10^+05^	2.5089 × 10^+07^	8.9328 × 10^+03^	1.0499 × 10^+04^	9.3551 × 10^+03^
F19	Best	2.2956 × 10^+03^	2.8393 × 10^+03^	2.7054 × 10^+03^	3.2722 × 10^+03^	3.3889 × 10^+03^	2.5596 × 10^+03^	3.4301 × 10^+03^	3.1897 × 10^+03^	3.0997 × 10^+03^	3.3714 × 10^+03^	2.4225 × 10^+03^	2.7224 × 10^+03^
	Mean	2.9171 × 10^+03^	3.3211 × 10^+03^	3.1776 × 10^+03^	3.7456 × 10^+03^	3.7497 × 10^+03^	3.0577 × 10^+03^	3.9887 × 10^+03^	3.7456 × 10^+03^	4.1401 × 10^+03^	3.8965 × 10^+03^	3.0447 × 10^+03^	3.1830 × 10^+03^
	Std	3.8150 × 10^+02^	2.6061 × 10^+02^	2.5092 × 10^+02^	2.2174 × 10^+02^	1.5138 × 10^+02^	3.0784 × 10^+02^	2.2626 × 10^+02^	2.8232 × 10^+02^	4.3098 × 10^+02^	2.0001 × 10^+02^	3.6533 × 10^+02^	2.6341 × 10^+02^
F20	Best	2.4162 × 10^+03^	2.5476 × 10^+03^	2.6602 × 10^+03^	2.4983 × 10^+03^	2.6024 × 10^+03^	2.4908 × 10^+03^	2.9026 × 10^+03^	2.6891 × 10^+03^	2.4993 × 10^+03^	2.6561 × 10^+03^	2.4240 × 10^+03^	2.4493 × 10^+03^
	Mean	2.4665 × 10^+03^	2.6488 × 10^+03^	2.7666 × 10^+03^	2.5563 × 10^+03^	2.7392 × 10^+03^	2.5570 × 10^+03^	2.9990 × 10^+03^	2.7708 × 10^+03^	2.7424 × 10^+03^	2.7022 × 10^+03^	2.5015 × 10^+03^	2.5342 × 10^+03^
	Std	2.6158 × 10^+01^	5.6267 × 10^+01^	5.4412 × 10^+01^	3.4922 × 10^+01^	7.0395 × 10^+01^	4.0659 × 10^+01^	5.6286 × 10^+01^	3.3866 × 10^+01^	1.2471 × 10^+02^	2.4280 × 10^+01^	4.4816 × 10^+01^	5.7910 × 10^+01^
F21	Best	2.3438 × 10^+03^	3.3427 × 10^+03^	1.1383 × 10^+04^	2.6095 × 10^+03^	4.0171 × 10^+03^	2.6977 × 10^+03^	1.5289 × 10^+04^	1.3648 × 10^+04^	1.3081 × 10^+04^	2.3356 × 10^+03^	2.4399 × 10^+03^	1.0796 × 10^+04^
	Mean	1.2781 × 10^+04^	1.2164 × 10^+04^	1.2821 × 10^+04^	1.5118 × 10^+04^	1.4561 × 10^+04^	9.7507 × 10^+03^	1.6239 × 10^+04^	1.5165 × 10^+04^	1.6106 × 10^+04^	1.3667 × 10^+04^	1.3607 × 10^+04^	1.4356 × 10^+04^
	Std	3.8961 × 10^+03^	3.5953 × 10^+03^	7.7124 × 10^+02^	3.6693 × 10^+03^	2.1705 × 10^+03^	1.6097 × 10^+03^	4.1327 × 10^+02^	6.5962 × 10^+02^	1.2969 × 10^+03^	5.5793 × 10^+03^	2.4562 × 10^+03^	1.0995 × 10^+03^
F22	Best	2.8093 × 10^+03^	3.0204 × 10^+03^	3.1089 × 10^+03^	3.0139 × 10^+03^	3.0990 × 10^+03^	2.9702 × 10^+03^	3.5063 × 10^+03^	3.2017 × 10^+03^	2.8739 × 10^+03^	3.0983 × 10^+03^	2.8978 × 10^+03^	2.9375 × 10^+03^
	Mean	2.9050 × 10^+03^	3.1733 × 10^+03^	3.3620 × 10^+03^	3.1106 × 10^+03^	3.2913 × 10^+03^	3.1083 × 10^+03^	3.7334 × 10^+03^	3.3113 × 10^+03^	3.1854 × 10^+03^	3.1488 × 10^+03^	3.0029 × 10^+03^	3.0488 × 10^+03^
	Std	3.8218 × 10^+01^	5.8076 × 10^+01^	1.3706 × 10^+02^	6.7034 × 10^+01^	1.1721 × 10^+02^	8.3459 × 10^+01^	1.1733 × 10^+02^	4.5255 × 10^+01^	1.8129 × 10^+02^	2.9774 × 10^+01^	5.8045 × 10^+01^	6.1160 × 10^+01^
F23	Best	2.9858 × 10^+03^	3.2065 × 10^+03^	3.3568 × 10^+03^	3.0999 × 10^+03^	3.1687 × 10^+03^	3.1595 × 10^+03^	3.7966 × 10^+03^	3.4028 × 10^+03^	3.1608 × 10^+03^	3.2238 × 10^+03^	3.0688 × 10^+03^	3.0801 × 10^+03^
	Mean	3.0821 × 10^+03^	3.3138 × 10^+03^	3.5254 × 10^+03^	3.2574 × 10^+03^	3.4192 × 10^+03^	3.2828 × 10^+03^	4.0035 × 10^+03^	3.4697 × 10^+03^	3.3712 × 10^+03^	3.3015 × 10^+03^	3.1548 × 10^+03^	3.2194 × 10^+03^
	Std	4.4550 × 10^+01^	5.9962 × 10^+01^	9.7242 × 10^+01^	7.9795 × 10^+01^	1.2294 × 10^+02^	8.0502 × 10^+01^	1.0609 × 10^+02^	3.8208 × 10^+01^	1.0069 × 10^+02^	2.8721 × 10^+01^	5.8210 × 10^+01^	7.2986 × 10^+01^
F24	Best	3.1144 × 10^+03^	3.5408 × 10^+03^	3.1713 × 10^+03^	3.1519 × 10^+03^	3.4713 × 10^+03^	3.3302 × 10^+03^	6.9342 × 10^+03^	3.6019 × 10^+03^	2.9360 × 10^+03^	3.0796 × 10^+03^	3.0876 × 10^+03^	3.0591 × 10^+03^
	Mean	3.1895 × 10^+03^	4.1291 × 10^+03^	3.3088 × 10^+03^	3.2466 × 10^+03^	3.9768 × 10^+03^	3.5478 × 10^+03^	9.5843 × 10^+03^	4.2582 × 10^+03^	4.6070 × 10^+03^	3.1607 × 10^+03^	3.1411 × 10^+03^	3.1516 × 10^+03^
	Std	4.3090 × 10^+01^	2.9653 × 10^+02^	8.8974 × 10^+01^	8.0069 × 10^+01^	4.1317 × 10^+02^	1.6858 × 10^+02^	1.1402 × 10^+03^	3.9892 × 10^+02^	3.7486 × 10^+03^	4.3968 × 10^+01^	4.1339 × 10^+01^	4.9422 × 10^+01^
F25	Best	5.3251 × 10^+03^	7.9215 × 10^+03^	3.7437 × 10^+03^	4.7957 × 10^+03^	9.4050 × 10^+03^	4.7828 × 10^+03^	1.1966 × 10^+04^	8.7076 × 10^+03^	5.3962 × 10^+03^	6.0018 × 10^+03^	5.7559 × 10^+03^	5.5701 × 10^+03^
	Mean	6.4977 × 10^+03^	1.0446 × 10^+04^	5.3865 × 10^+03^	9.4738 × 10^+03^	1.2191 × 10^+04^	8.9264 × 10^+03^	1.4027 × 10^+04^	9.5836 × 10^+03^	7.7463 × 10^+03^	7.6088 × 10^+03^	7.1475 × 10^+03^	7.0804 × 10^+03^
	Std	7.7477 × 10^+02^	1.2439 × 10^+03^	2.0702 × 10^+03^	2.1979 × 10^+03^	1.3195 × 10^+03^	1.6300 × 10^+03^	9.6034 × 10^+02^	4.4109 × 10^+02^	1.1180 × 10^+03^	4.5403 × 10^+02^	9.7943 × 10^+02^	7.3365 × 10^+02^
F26	Best	3.3670 × 10^+03^	3.5782 × 10^+03^	3.6600 × 10^+03^	3.4365 × 10^+03^	3.5022 × 10^+03^	3.5695 × 10^+03^	3.7726 × 10^+03^	3.6632 × 10^+03^	3.2000 × 10^+03^	3.3325 × 10^+03^	3.2697 × 10^+03^	3.3735 × 10^+03^
	Mean	3.5067 × 10^+03^	3.8211 × 10^+03^	4.0629 × 10^+03^	3.7087 × 10^+03^	3.8614 × 10^+03^	3.8282 × 10^+03^	4.0786 × 10^+03^	3.9140 × 10^+03^	3.2000 × 10^+03^	3.4133 × 10^+03^	3.4805 × 10^+03^	3.5209 × 10^+03^
	Std	9.9061 × 10^+01^	1.6097 × 10^+02^	2.3645 × 10^+02^	1.6975 × 10^+02^	1.5990 × 10^+02^	1.7281 × 10^+02^	2.0397 × 10^+02^	1.3122 × 10^+02^	1.3118 × 10^−04^	5.9771 × 10^+01^	1.0098 × 10^+02^	9.8928 × 10^+01^
F27	Best	3.4049 × 10^+03^	4.4627 × 10^+03^	3.5717 × 10^+03^	3.4335 × 10^+03^	3.6483 × 10^+03^	3.5876 × 10^+03^	6.9606 × 10^+03^	4.0540 × 10^+03^	3.3000 × 10^+03^	3.3799 × 10^+03^	3.3715 × 10^+03^	3.3424 × 10^+03^
	Mean	3.5344 × 10^+03^	4.8012 × 10^+03^	3.8860 × 10^+03^	3.6303 × 10^+03^	4.4083 × 10^+03^	4.2130 × 10^+03^	8.6873 × 10^+03^	4.9695 × 10^+03^	3.3000 × 10^+03^	3.5500 × 10^+03^	3.4772 × 10^+03^	3.4843 × 10^+03^
	Std	8.6606 × 10^+01^	2.7118 × 10^+02^	1.9154 × 10^+02^	1.9861 × 10^+02^	3.6079 × 10^+02^	2.7731 × 10^+02^	9.3440 × 10^+02^	5.4663 × 10^+02^	1.7169 × 10^−04^	1.0582 × 10^+02^	6.5544 × 10^+01^	1.2935 × 10^+02^
F28	Best	3.6098 × 10^+03^	4.9207 × 10^+03^	5.5750 × 10^+03^	4.1126 × 10^+03^	4.7465 × 10^+03^	4.1485 × 10^+03^	6.6463 × 10^+03^	5.3806 × 10^+03^	4.5860 × 10^+03^	4.0876 × 10^+03^	3.6839 × 10^+03^	3.9289 × 10^+03^
	Mean	4.2602 × 10^+03^	5.6111 × 10^+03^	6.6996 × 10^+03^	4.8618 × 10^+03^	5.5489 × 10^+03^	4.6875 × 10^+03^	7.5336 × 10^+03^	6.1074 × 10^+03^	6.4592 × 10^+03^	4.8673 × 10^+03^	4.4613 × 10^+03^	4.6635 × 10^+03^
	Std	2.9956 × 10^+02^	4.6239 × 10^+02^	7.6869 × 10^+02^	4.8339 × 10^+02^	4.6864 × 10^+02^	2.8474 × 10^+02^	5.0872 × 10^+02^	3.9704 × 10^+02^	1.3132 × 10^+03^	3.6966 × 10^+02^	4.4554 × 10^+02^	4.3502 × 10^+02^
F29	Best	1.1849 × 10^+06^	7.8104 × 10^+07^	6.8566 × 10^+07^	7.6620 × 10^+05^	9.8327 × 10^+06^	5.8864 × 10^+06^	8.8323 × 10^+07^	2.7305 × 10^+07^	4.9494 × 10^+03^	2.3066 × 10^+06^	7.2884 × 10^+05^	9.8489 × 10^+05^
	Mean	2.7094 × 10^+06^	1.4361 × 10^+08^	1.2402 × 10^+08^	1.0909 × 10^+06^	3.5247 × 10^+07^	1.1330 × 10^+07^	2.2681 × 10^+08^	5.3513 × 10^+07^	7.0463 × 10^+07^	3.4365 × 10^+06^	1.3108 × 10^+06^	1.8524 × 10^+06^
	Std	1.3067 × 10^+06^	3.9025 × 10^+07^	2.5312 × 10^+07^	2.2000 × 10^+05^	2.0778 × 10^+07^	4.6536 × 10^+06^	9.6335 × 10^+07^	1.5513 × 10^+07^	1.1052 × 10^+08^	8.3554 × 10^+05^	3.7930 × 10^+05^	6.7129 × 10^+05^

**Table A4:** Comparison of results on CEC 2017 (D = 100).

Function	Metric	MICFOA	CFOA	PEOA	TLBO	COA	ARO	EDO	YDSE	LSHADE	JADE	IDE-EDA	APSM-JSO
F1	Best	1.8088 × 10^+09^	5.8305 × 10^+10^	1.8170 × 10^+10^	5.7438 × 10^+09^	3.5337 × 10^+10^	3.1640 × 10^+10^	1.8851 × 10^+11^	7.3864 × 10^+10^	7.3193 × 10^+07^	3.1546 × 10^+09^	5.1172 × 10^+09^	4.7897 × 10^+09^
	Mean	2.9701 × 10^+09^	6.7379 × 10^+10^	2.3593 × 10^+10^	1.8756 × 10^+10^	6.7834 × 10^+10^	5.0140 × 10^+10^	2.2551 × 10^+11^	9.1872 × 10^+10^	4.7557 × 10^+10^	7.9427 × 10^+09^	1.2396 × 10^+10^	1.0933 × 10^+10^
	Std	5.2189 × 10^+08^	6.1014 × 10^+09^	3.4128 × 10^+09^	5.4612 × 10^+09^	1.3229 × 10^+10^	9.7228 × 10^+09^	1.4998 × 10^+10^	9.2634 × 10^+09^	8.0225 × 10^+10^	3.1002 × 10^+09^	5.4651 × 10^+09^	3.5965 × 10^+09^
F2	Best	1.9152 × 10^+05^	3.4038 × 10^+05^	1.8299 × 10^+05^	4.1800 × 10^+05^	4.7710 × 10^+05^	2.8109 × 10^+05^	2.6710 × 10^+05^	2.3020 × 10^+05^	4.3724 × 10^+05^	4.1018 × 10^+05^	1.8719 × 10^+05^	2.0074 × 10^+05^
	Mean	2.3971 × 10^+05^	4.4166 × 10^+05^	2.1331 × 10^+05^	5.2679 × 10^+05^	7.3971 × 10^+05^	3.6547 × 10^+05^	3.1911 × 10^+05^	3.0099 × 10^+05^	1.2893 × 10^+06^	5.2219 × 10^+05^	2.6335 × 10^+05^	2.7220 × 10^+05^
	Std	2.6927 × 10^+04^	6.1366 × 10^+04^	1.5855 × 10^+04^	7.7200 × 10^+04^	1.3200 × 10^+05^	3.4370 × 10^+04^	2.7642 × 10^+04^	3.1105 × 10^+04^	5.2644 × 10^+05^	5.7423 × 10^+04^	3.6280 × 10^+04^	4.0081 × 10^+04^
F3	Best	1.2303 × 10^+03^	6.4745 × 10^+03^	1.2353 × 10^+03^	1.6127 × 10^+03^	5.2761 × 10^+03^	3.8049 × 10^+03^	4.0997 × 10^+04^	7.5173 × 10^+03^	9.0686 × 10^+02^	1.2414 × 10^+03^	1.4320 × 10^+03^	1.3298 × 10^+03^
	Mean	1.4823 × 10^+03^	9.4405 × 10^+03^	1.4549 × 10^+03^	2.8528 × 10^+03^	9.2627 × 10^+03^	5.9634 × 10^+03^	6.9036 × 10^+04^	1.1972 × 10^+04^	1.4459 × 10^+04^	1.6412 × 10^+03^	1.9940 × 10^+03^	2.0145 × 10^+03^
	Std	1.7281 × 10^+02^	1.9185 × 10^+03^	1.6573 × 10^+02^	1.0109 × 10^+03^	3.0199 × 10^+03^	1.0862 × 10^+03^	1.3650 × 10^+04^	2.4172 × 10^+03^	2.4197 × 10^+04^	2.3491 × 10^+02^	3.9201 × 10^+02^	4.3631 × 10^+02^
F4	Best	1.0517 × 10^+03^	1.4360 × 10^+03^	1.5480 × 10^+03^	1.1672 × 10^+03^	1.4371 × 10^+03^	1.2438 × 10^+03^	1.8979 × 10^+03^	1.6494 × 10^+03^	1.0417 × 10^+03^	1.4601 × 10^+03^	1.1167 × 10^+03^	1.0861 × 10^+03^
	Mean	1.1785 × 10^+03^	1.5926 × 10^+03^	1.6833 × 10^+03^	1.3240 × 10^+03^	1.5229 × 10^+03^	1.4018 × 10^+03^	1.9922 × 10^+03^	1.7679 × 10^+03^	1.6651 × 10^+03^	1.5363 × 10^+03^	1.2444 × 10^+03^	1.2969 × 10^+03^
	Std	6.3657 × 10^+01^	8.0792 × 10^+01^	6.7422 × 10^+01^	1.2175 × 10^+02^	5.2195 × 10^+01^	6.6268 × 10^+01^	4.9992 × 10^+01^	6.3975 × 10^+01^	2.7484 × 10^+02^	4.1787 × 10^+01^	8.8242 × 10^+01^	1.1000 × 10^+02^
F5	Best	6.3519 × 10^+02^	6.6577 × 10^+02^	6.6889 × 10^+02^	6.4295 × 10^+02^	6.6659 × 10^+02^	6.4180 × 10^+02^	6.9369 × 10^+02^	6.8097 × 10^+02^	6.0119 × 10^+02^	6.1092 × 10^+02^	6.3820 × 10^+02^	6.3755 × 10^+02^
	Mean	6.4443 × 10^+02^	6.7644 × 10^+02^	6.8658 × 10^+02^	6.5598 × 10^+02^	6.7412 × 10^+02^	6.5629 × 10^+02^	7.0645 × 10^+02^	6.9268 × 10^+02^	6.4478 × 10^+02^	6.1651 × 10^+02^	6.5025 × 10^+02^	6.5613 × 10^+02^
	Std	4.0462 × 10^+00^	5.9278 × 10^+00^	5.4068 × 10^+00^	6.4346 × 10^+00^	3.7172 × 10^+00^	7.7084 × 10^+00^	4.2412 × 10^+00^	6.9174 × 10^+00^	3.9556 × 10^+01^	3.8061 × 10^+00^	6.9843 × 10^+00^	1.0902 × 10^+01^
F6	Best	1.8457 × 10^+03^	2.6342 × 10^+03^	2.5677 × 10^+03^	2.1475 × 10^+03^	3.0480 × 10^+03^	2.4989 × 10^+03^	3.4746 × 10^+03^	2.8961 × 10^+03^	1.7425 × 10^+03^	1.8835 × 10^+03^	2.0144 × 10^+03^	1.9491 × 10^+03^
	Mean	2.2377 × 10^+03^	3.0223 × 10^+03^	3.0204 × 10^+03^	2.5348 × 10^+03^	3.3820 × 10^+03^	2.8816 × 10^+03^	3.7651 × 10^+03^	3.2186 × 10^+03^	2.7562 × 10^+03^	2.0353 × 10^+03^	2.4835 × 10^+03^	2.5709 × 10^+03^
	Std	2.1676 × 10^+02^	2.0055 × 10^+02^	2.4448 × 10^+02^	1.8681 × 10^+02^	1.3185 × 10^+02^	2.1548 × 10^+02^	1.3822 × 10^+02^	1.5861 × 10^+02^	1.4419 × 10^+03^	9.5392 × 10^+01^	1.9981 × 10^+02^	2.9156 × 10^+02^
F7	Best	1.4152 × 10^+03^	1.8225 × 10^+03^	1.9295 × 10^+03^	1.4842 × 10^+03^	1.7897 × 10^+03^	1.6085 × 10^+03^	2.3147 × 10^+03^	1.9608 × 10^+03^	1.4810 × 10^+03^	1.7310 × 10^+03^	1.4066 × 10^+03^	1.5178 × 10^+03^
	Mean	1.5183 × 10^+03^	1.9453 × 10^+03^	2.0836 × 10^+03^	1.6917 × 10^+03^	2.0165 × 10^+03^	1.7889 × 10^+03^	2.4528 × 10^+03^	2.0884 × 10^+03^	1.9796 × 10^+03^	1.8496 × 10^+03^	1.5823 × 10^+03^	1.6464 × 10^+03^
	Std	7.4099 × 10^+01^	7.8084 × 10^+01^	6.4847 × 10^+01^	1.4021 × 10^+02^	6.1580 × 10^+01^	1.0689 × 10^+02^	5.4594 × 10^+01^	6.7044 × 10^+01^	2.7700 × 10^+02^	4.6856 × 10^+01^	9.6625 × 10^+01^	9.7122 × 10^+01^
F8	Best	1.8085 × 10^+04^	3.8778 × 10^+04^	4.8441 × 10^+04^	3.9025 × 10^+04^	3.2796 × 10^+04^	2.5926 × 10^+04^	6.6685 × 10^+04^	4.8627 × 10^+04^	4.8262 × 10^+03^	9.5332 × 10^+03^	2.9631 × 10^+04^	2.9664 × 10^+04^
	Mean	4.0310 × 10^+04^	4.9629 × 10^+04^	5.9606 × 10^+04^	5.6868 × 10^+04^	4.6523 × 10^+04^	3.4782 × 10^+04^	7.6970 × 10^+04^	6.9962 × 10^+04^	5.8558 × 10^+04^	1.9251 × 10^+04^	5.3014 × 10^+04^	5.2954 × 10^+04^
	Std	9.3601 × 10^+03^	5.4490 × 10^+03^	4.7637 × 10^+03^	6.6545 × 10^+03^	1.0247 × 10^+04^	4.6256 × 10^+03^	4.1524 × 10^+03^	9.9306 × 10^+03^	4.8816 × 10^+04^	5.7643 × 10^+03^	9.6767 × 10^+03^	1.3028 × 10^+04^
F9	Best	1.6431 × 10^+04^	2.5845 × 10^+04^	2.3289 × 10^+04^	3.1141 × 10^+04^	1.9906 × 10^+04^	1.6246 × 10^+04^	2.9532 × 10^+04^	2.8762 × 10^+04^	2.6450 × 10^+04^	3.0395 × 10^+04^	2.4723 × 10^+04^	2.4576 × 10^+04^
	Mean	2.8033 × 10^+04^	2.8231 × 10^+04^	2.6058 × 10^+04^	3.2165 × 10^+04^	2.4580 × 10^+04^	1.9696 × 10^+04^	3.1498 × 10^+04^	3.0647 × 10^+04^	3.2917 × 10^+04^	3.2098 × 10^+04^	2.9007 × 10^+04^	2.8824 × 10^+04^
	Std	3.8393 × 10^+03^	1.1528 × 10^+03^	1.6164 × 10^+03^	5.0949 × 10^+02^	2.9029 × 10^+03^	1.9294 × 10^+03^	9.7906 × 10^+02^	7.3778 × 10^+02^	1.5944 × 10^+03^	7.4951 × 10^+02^	1.5196 × 10^+03^	1.5465 × 10^+03^
F10	Best	9.2131 × 10^+03^	8.0683 × 10^+04^	7.6818 × 10^+03^	2.9962 × 10^+04^	1.4256 × 10^+05^	5.5999 × 10^+04^	7.0797 × 10^+04^	5.7235 × 10^+04^	8.3968 × 10^+04^	5.6020 × 10^+04^	9.1663 × 10^+03^	6.5442 × 10^+03^
	Mean	1.3166 × 10^+04^	1.1018 × 10^+05^	1.4408 × 10^+04^	6.2770 × 10^+04^	3.0691 × 10^+05^	8.2632 × 10^+04^	1.2643 × 10^+05^	7.7874 × 10^+04^	4.9119 × 10^+05^	9.6015 × 10^+04^	2.1832 × 10^+04^	2.0031 × 10^+04^
	Std	3.1754 × 10^+03^	1.6093 × 10^+04^	4.3289 × 10^+03^	1.9723 × 10^+04^	9.7581 × 10^+04^	1.8167 × 10^+04^	3.0749 × 10^+04^	1.3035 × 10^+04^	2.2194 × 10^+05^	1.8046 × 10^+04^	8.3545 × 10^+03^	8.4999 × 10^+03^
F11	Best	1.5019 × 10^+08^	3.8558 × 10^+09^	4.4833 × 10^+08^	1.7349 × 10^+08^	4.0300 × 10^+09^	1.7870 × 10^+09^	7.8264 × 10^+10^	8.2411 × 10^+09^	6.0952 × 10^+08^	1.1663 × 10^+08^	1.2780 × 10^+08^	9.4395 × 10^+07^
	Mean	4.1613 × 10^+08^	8.3283 × 10^+09^	1.0119 × 10^+09^	1.0729 × 10^+09^	1.5876 × 10^+10^	4.5452 × 10^+09^	1.3091 × 10^+11^	1.5520 × 10^+10^	2.0387 × 10^+10^	3.8062 × 10^+08^	4.3229 × 10^+08^	4.2819 × 10^+08^
	Std	1.5736 × 10^+08^	2.4797 × 10^+09^	2.8820 × 10^+08^	8.8138 × 10^+08^	8.6287 × 10^+09^	2.4669 × 10^+09^	2.0401 × 10^+10^	3.3820 × 10^+09^	2.5423 × 10^+10^	3.0856 × 10^+08^	2.4172 × 10^+08^	2.8743 × 10^+08^
F12	Best	2.2312 × 10^+04^	3.7081 × 10^+07^	2.4299 × 10^+04^	2.3663 × 10^+04^	7.9728 × 10^+06^	3.6502 × 10^+06^	8.9056 × 10^+09^	4.4054 × 10^+08^	1.8186 × 10^+04^	1.2868 × 10^+04^	1.6737 × 10^+04^	2.5954 × 10^+04^
	Mean	3.8757 × 10^+04^	1.4821 × 10^+08^	4.8362 × 10^+04^	6.8779 × 10^+04^	9.5415 × 10^+08^	1.7760 × 10^+07^	2.2688 × 10^+10^	9.1032 × 10^+08^	2.6426 × 10^+08^	2.2366 × 10^+04^	7.8005 × 10^+04^	9.2125 × 10^+04^
	Std	1.2859 × 10^+04^	5.8555 × 10^+07^	1.7892 × 10^+04^	9.0667 × 10^+04^	1.5955 × 10^+09^	1.4185 × 10^+07^	6.9401 × 10^+09^	3.3931 × 10^+08^	7.5121 × 10^+08^	7.3454 × 10^+03^	7.3388 × 10^+04^	1.0832 × 10^+05^
F13	Best	2.1902 × 10^+04^	1.4550 × 10^+06^	9.0316 × 10^+05^	2.7461 × 10^+05^	1.4245 × 10^+06^	2.2991 × 10^+06^	1.1535 × 10^+06^	1.9857 × 10^+05^	2.1309 × 10^+06^	2.4586 × 10^+06^	1.6465 × 10^+05^	8.2224 × 10^+04^
	Mean	1.9147 × 10^+05^	4.4471 × 10^+06^	2.1025 × 10^+06^	2.0232 × 10^+06^	9.6828 × 10^+06^	6.1752 × 10^+06^	3.2619 × 10^+06^	8.5977 × 10^+05^	6.4894 × 10^+07^	6.0014 × 10^+06^	6.3688 × 10^+05^	5.7509 × 10^+05^
	Std	2.5169 × 10^+05^	2.0103 × 10^+06^	8.8568 × 10^+05^	1.2227 × 10^+06^	7.3799 × 10^+06^	3.2621 × 10^+06^	1.4710 × 10^+06^	4.2029 × 10^+05^	5.5916 × 10^+07^	2.1645 × 10^+06^	4.2512 × 10^+05^	4.1012 × 10^+05^
F14	Best	3.1418 × 10^+03^	2.8654 × 10^+05^	1.2437 × 10^+04^	3.3725 × 10^+03^	1.3131 × 10^+05^	5.4405 × 10^+04^	5.4439 × 10^+08^	1.3113 × 10^+07^	3.5474 × 10^+03^	3.6574 × 10^+03^	2.9308 × 10^+03^	5.1292 × 10^+03^
	Mean	6.8270 × 10^+03^	1.7522 × 10^+06^	3.4632 × 10^+04^	8.7079 × 10^+03^	3.1095 × 10^+07^	3.4070 × 10^+05^	4.1963 × 10^+09^	5.3965 × 10^+07^	5.1549 × 10^+07^	7.5131 × 10^+03^	9.0156 × 10^+03^	1.5195 × 10^+04^
	Std	2.4495 × 10^+03^	1.2213 × 10^+06^	1.4117 × 10^+04^	4.2653 × 10^+03^	1.3168 × 10^+08^	5.3099 × 10^+05^	3.8259 × 10^+09^	2.6699 × 10^+07^	1.5014 × 10^+08^	3.2072 × 10^+03^	4.2877 × 10^+03^	1.1530 × 10^+04^
F15	Best	3.8624 × 10^+03^	6.7865 × 10^+03^	8.8219 × 10^+03^	4.4024 × 10^+03^	6.6724 × 10^+03^	5.4054 × 10^+03^	1.1566 × 10^+04^	9.0772 × 10^+03^	1.0099 × 10^+04^	9.0966 × 10^+03^	4.0453 × 10^+03^	3.8342 × 10^+03^
	Mean	5.4147 × 10^+03^	8.0639 × 10^+03^	1.0877 × 10^+04^	6.2995 × 10^+03^	8.7535 × 10^+03^	6.8750 × 10^+03^	1.4738 × 10^+04^	1.0492 × 10^+04^	1.2365 × 10^+04^	1.0065 × 10^+04^	5.9802 × 10^+03^	6.8288 × 10^+03^
	Std	7.5603 × 10^+02^	7.2560 × 10^+02^	1.2097 × 10^+03^	1.0785 × 10^+03^	1.0639 × 10^+03^	7.1700 × 10^+02^	1.7985 × 10^+03^	4.9098 × 10^+02^	1.2119 × 10^+03^	5.0527 × 10^+02^	9.5688 × 10^+02^	1.1828 × 10^+03^
F16	Best	3.5604 × 10^+03^	4.7767 × 10^+03^	4.5275 × 10^+03^	4.1332 × 10^+03^	5.2956 × 10^+03^	4.9403 × 10^+03^	8.1350 × 10^+03^	6.8366 × 10^+03^	7.4627 × 10^+03^	6.2048 × 10^+03^	3.9293 × 10^+03^	4.2298 × 10^+03^
	Mean	4.4020 × 10^+03^	5.9618 × 10^+03^	6.0903 × 10^+03^	5.4440 × 10^+03^	6.7523 × 10^+03^	5.5166 × 10^+03^	2.4037 × 10^+04^	7.6285 × 10^+03^	1.0972 × 10^+04^	7.0382 × 10^+03^	4.9454 × 10^+03^	5.1440 × 10^+03^
	Std	4.5368 × 10^+02^	6.9351 × 10^+02^	6.0296 × 10^+02^	7.7659 × 10^+02^	9.1838 × 10^+02^	4.1188 × 10^+02^	3.1519 × 10^+04^	4.0686 × 10^+02^	4.0763 × 10^+03^	2.8133 × 10^+02^	6.3645 × 10^+02^	5.0577 × 10^+02^
F17	Best	1.1181 × 10^+05^	1.6035 × 10^+06^	1.2436 × 10^+06^	9.2087 × 10^+05^	2.3683 × 10^+06^	1.9900 × 10^+06^	2.4158 × 10^+06^	5.2597 × 10^+05^	5.4478 × 10^+06^	3.9464 × 10^+06^	2.1032 × 10^+05^	2.3077 × 10^+05^
	Mean	3.7516 × 10^+05^	4.5801 × 10^+06^	2.4878 × 10^+06^	3.0775 × 10^+06^	1.2067 × 10^+07^	5.6350 × 10^+06^	5.7367 × 10^+06^	1.3518 × 10^+06^	1.5039 × 10^+08^	9.3883 × 10^+06^	8.2256 × 10^+05^	8.0413 × 10^+05^
	Std	2.9435 × 10^+05^	2.4567 × 10^+06^	8.4374 × 10^+05^	1.5483 × 10^+06^	1.1245 × 10^+07^	2.8588 × 10^+06^	2.8636 × 10^+06^	8.3889 × 10^+05^	1.1153 × 10^+08^	2.5883 × 10^+06^	5.9838 × 10^+05^	4.9102 × 10^+05^
F18	Best	2.7282 × 10^+03^	2.2934 × 10^+06^	3.8753 × 10^+05^	2.5680 × 10^+03^	2.8733 × 10^+06^	2.6460 × 10^+05^	8.7824 × 10^+08^	2.4846 × 10^+07^	2.5326 × 10^+03^	2.4057 × 10^+03^	2.8209 × 10^+03^	3.3285 × 10^+03^
	Mean	1.0633 × 10^+04^	8.9331 × 10^+06^	7.3659 × 10^+06^	1.0667 × 10^+04^	1.7406 × 10^+07^	6.4198 × 10^+05^	4.3720 × 10^+09^	6.8678 × 10^+07^	7.6583 × 10^+07^	7.8127 × 10^+03^	1.1523 × 10^+04^	1.4428 × 10^+04^
	Std	7.6416 × 10^+03^	9.3045 × 10^+06^	5.3223 × 10^+06^	1.4773 × 10^+04^	1.6650 × 10^+07^	4.5312 × 10^+05^	3.0215 × 10^+09^	2.4062 × 10^+07^	2.1348 × 10^+08^	5.0275 × 10^+03^	1.0261 × 10^+04^	1.1127 × 10^+04^
F19	Best	4.0056 × 10^+03^	5.3926 × 10^+03^	4.6814 × 10^+03^	6.8283 × 10^+03^	5.7557 × 10^+03^	4.1984 × 10^+03^	7.2138 × 10^+03^	6.4361 × 10^+03^	7.3284 × 10^+03^	6.5940 × 10^+03^	5.0369 × 10^+03^	5.5775 × 10^+03^
	Mean	5.5533 × 10^+03^	6.1121 × 10^+03^	5.7108 × 10^+03^	7.3583 × 10^+03^	7.0468 × 10^+03^	5.1871 × 10^+03^	7.7274 × 10^+03^	7.2761 × 10^+03^	8.2753 × 10^+03^	7.5304 × 10^+03^	6.2881 × 10^+03^	6.4088 × 10^+03^
	Std	8.2973 × 10^+02^	5.0005 × 10^+02^	5.4371 × 10^+02^	2.7408 × 10^+02^	5.9526 × 10^+02^	4.1800 × 10^+02^	2.9180 × 10^+02^	4.0652 × 10^+02^	5.2934 × 10^+02^	3.7210 × 10^+02^	6.0307 × 10^+02^	4.5608 × 10^+02^
F20	Best	2.8341 × 10^+03^	3.2773 × 10^+03^	3.5194 × 10^+03^	3.0540 × 10^+03^	3.3848 × 10^+03^	2.9252 × 10^+03^	4.0360 × 10^+03^	3.5189 × 10^+03^	3.1180 × 10^+03^	3.2719 × 10^+03^	2.9825 × 10^+03^	2.8954 × 10^+03^
	Mean	2.9445 × 10^+03^	3.4404 × 10^+03^	3.7832 × 10^+03^	3.2614 × 10^+03^	3.7362 × 10^+03^	3.2396 × 10^+03^	4.2596 × 10^+03^	3.6291 × 10^+03^	3.5270 × 10^+03^	3.3706 × 10^+03^	3.0996 × 10^+03^	3.1878 × 10^+03^
	Std	4.9673 × 10^+01^	7.6675 × 10^+01^	1.3360 × 10^+02^	1.2099 × 10^+02^	1.8359 × 10^+02^	1.2670 × 10^+02^	1.3351 × 10^+02^	6.7092 × 10^+01^	3.1486 × 10^+02^	4.4162 × 10^+01^	7.5360 × 10^+01^	1.2192 × 10^+02^
F21	Best	2.0056 × 10^+04^	2.6167 × 10^+04^	2.5849 × 10^+04^	3.3324 × 10^+04^	2.5293 × 10^+04^	8.9991 × 10^+03^	3.1401 × 10^+04^	2.9861 × 10^+04^	2.9403 × 10^+04^	3.3961 × 10^+04^	2.4476 × 10^+04^	2.8252 × 10^+04^
	Mean	3.1526 × 10^+04^	3.0814 × 10^+04^	2.8786 × 10^+04^	3.4394 × 10^+04^	3.0116 × 10^+04^	2.2554 × 10^+04^	3.4270 × 10^+04^	3.2649 × 10^+04^	3.4960 × 10^+04^	3.4830 × 10^+04^	3.1641 × 10^+04^	3.1739 × 10^+04^
	Std	2.2446 × 10^+03^	1.6516 × 10^+03^	1.4830 × 10^+03^	6.5093 × 10^+02^	2.1042 × 10^+03^	2.9337 × 10^+03^	8.7786 × 10^+02^	1.0450 × 10^+03^	1.5134 × 10^+03^	5.1630 × 10^+02^	2.1833 × 10^+03^	1.3096 × 10^+03^
F22	Best	3.2368 × 10^+03^	3.8578 × 10^+03^	4.2124 × 10^+03^	3.5491 × 10^+03^	3.9795 × 10^+03^	3.6556 × 10^+03^	5.1360 × 10^+03^	4.2094 × 10^+03^	3.4706 × 10^+03^	3.3170 × 10^+03^	3.4865 × 10^+03^	3.5214 × 10^+03^
	Mean	3.4317 × 10^+03^	4.0785 × 10^+03^	4.6111 × 10^+03^	3.8811 × 10^+03^	4.2562 × 10^+03^	3.8304 × 10^+03^	5.5406 × 10^+03^	4.3802 × 10^+03^	4.0337 × 10^+03^	3.7331 × 10^+03^	3.6902 × 10^+03^	3.8877 × 10^+03^
	Std	8.1168 × 10^+01^	1.1688 × 10^+02^	2.2006 × 10^+02^	1.6902 × 10^+02^	1.6916 × 10^+02^	1.0558 × 10^+02^	1.9433 × 10^+02^	1.0584 × 10^+02^	3.4370 × 10^+02^	2.0145 × 10^+02^	1.2901 × 10^+02^	1.6450 × 10^+02^
F23	Best	3.9273 × 10^+03^	4.6045 × 10^+03^	4.9079 × 10^+03^	4.4870 × 10^+03^	4.8240 × 10^+03^	4.5954 × 10^+03^	6.3689 × 10^+03^	5.0584 × 10^+03^	3.8401 × 10^+03^	3.6035 × 10^+03^	4.0548 × 10^+03^	4.1166 × 10^+03^
	Mean	4.0973 × 10^+03^	4.9192 × 10^+03^	5.5674 × 10^+03^	4.8811 × 10^+03^	5.4698 × 10^+03^	4.9367 × 10^+03^	7.3421 × 10^+03^	5.2637 × 10^+03^	4.7174 × 10^+03^	4.0939 × 10^+03^	4.3025 × 10^+03^	4.5034 × 10^+03^
	Std	1.0468 × 10^+02^	1.6958 × 10^+02^	3.6329 × 10^+02^	2.9373 × 10^+02^	4.2155 × 10^+02^	1.8949 × 10^+02^	5.3460 × 10^+02^	1.0611 × 10^+02^	5.4512 × 10^+02^	2.4109 × 10^+02^	1.9549 × 10^+02^	1.9987 × 10^+02^
F24	Best	3.8872 × 10^+03^	6.7965 × 10^+03^	4.4613 × 10^+03^	4.3954 × 10^+03^	6.2979 × 10^+03^	5.1778 × 10^+03^	1.6384 × 10^+04^	8.1554 × 10^+03^	3.5487 × 10^+03^	4.0378 × 10^+03^	3.9080 × 10^+03^	4.0509 × 10^+03^
	Mean	4.2204 × 10^+03^	8.2739 × 10^+03^	4.9799 × 10^+03^	5.0970 × 10^+03^	8.1549 × 10^+03^	6.5219 × 10^+03^	2.1903 × 10^+04^	9.9625 × 10^+03^	1.2994 × 10^+04^	4.4234 × 10^+03^	4.4945 × 10^+03^	4.4976 × 10^+03^
	Std	1.5741 × 10^+02^	6.7589 × 10^+02^	3.1290 × 10^+02^	4.5288 × 10^+02^	1.2014 × 10^+03^	6.2813 × 10^+02^	2.4244 × 10^+03^	1.3299 × 10^+03^	1.6420 × 10^+04^	2.5134 × 10^+02^	3.0795 × 10^+02^	3.2378 × 10^+02^
F25	Best	1.4064 × 10^+04^	2.3116 × 10^+04^	9.9227 × 10^+03^	1.8394 × 10^+04^	2.2560 × 10^+04^	2.1301 × 10^+04^	2.9289 × 10^+04^	2.1267 × 10^+04^	1.2832 × 10^+04^	1.1454 × 10^+04^	1.5184 × 10^+04^	1.4071 × 10^+04^
	Mean	1.7444 × 10^+04^	2.8870 × 10^+04^	2.0766 × 10^+04^	2.6419 × 10^+04^	3.2010 × 10^+04^	2.5931 × 10^+04^	3.8138 × 10^+04^	2.3123 × 10^+04^	2.1210 × 10^+04^	1.4940 × 10^+04^	1.9278 × 10^+04^	1.9251 × 10^+04^
	Std	2.2339 × 10^+03^	2.3407 × 10^+03^	6.8232 × 10^+03^	3.7272 × 10^+03^	3.2423 × 10^+03^	2.2526 × 10^+03^	4.8742 × 10^+03^	9.6582 × 10^+02^	5.5147 × 10^+03^	2.3788 × 10^+03^	2.3581 × 10^+03^	2.8185 × 10^+03^
F26	Best	3.7880 × 10^+03^	4.3898 × 10^+03^	4.0657 × 10^+03^	3.9727 × 10^+03^	4.0519 × 10^+03^	4.1090 × 10^+03^	4.8069 × 10^+03^	4.4508 × 10^+03^	3.2000 × 10^+03^	3.5572 × 10^+03^	3.6285 × 10^+03^	3.5181 × 10^+03^
	Mean	4.0173 × 10^+03^	4.8495 × 10^+03^	4.6497 × 10^+03^	4.4181 × 10^+03^	4.6001 × 10^+03^	4.5316 × 10^+03^	6.2608 × 10^+03^	4.9092 × 10^+03^	3.2000 × 10^+03^	3.7308 × 10^+03^	3.9096 × 10^+03^	3.9460 × 10^+03^
	Std	1.6719 × 10^+02^	2.5104 × 10^+02^	3.5689 × 10^+02^	2.2501 × 10^+02^	3.1041 × 10^+02^	2.2394 × 10^+02^	5.4935 × 10^+02^	2.6391 × 10^+02^	1.2064 × 10^−04^	8.2671 × 10^+01^	1.5377 × 10^+02^	1.9157 × 10^+02^
F27	Best	4.2940 × 10^+03^	9.8188 × 10^+03^	5.7914 × 10^+03^	5.4831 × 10^+03^	7.9239 × 10^+03^	7.9002 × 10^+03^	1.7966 × 10^+04^	1.1125 × 10^+04^	3.3000 × 10^+03^	4.5346 × 10^+03^	4.3727 × 10^+03^	4.6971 × 10^+03^
	Mean	4.7774 × 10^+03^	1.1778 × 10^+04^	6.9063 × 10^+03^	6.7779 × 10^+03^	1.1186 × 10^+04^	9.5882 × 10^+03^	2.3213 × 10^+04^	1.4417 × 10^+04^	3.3000 × 10^+03^	5.6789 × 10^+03^	5.7441 × 10^+03^	5.7811 × 10^+03^
	Std	3.4492 × 10^+02^	1.1061 × 10^+03^	6.5965 × 10^+02^	8.9757 × 10^+02^	1.5279 × 10^+03^	9.4230 × 10^+02^	2.2626 × 10^+03^	1.4167 × 10^+03^	1.1954 × 10^−04^	6.7256 × 10^+02^	7.0552 × 10^+02^	7.4699 × 10^+02^
F28	Best	6.4033 × 10^+03^	8.9332 × 10^+03^	1.0508 × 10^+04^	7.1880 × 10^+03^	9.1907 × 10^+03^	6.8189 × 10^+03^	1.4725 × 10^+04^	1.1371 × 10^+04^	9.6561 × 10^+03^	8.3983 × 10^+03^	6.2048 × 10^+03^	6.5756 × 10^+03^
	Mean	7.8077 × 10^+03^	1.1601 × 10^+04^	1.2383 × 10^+04^	8.5773 × 10^+03^	1.1104 × 10^+04^	8.9178 × 10^+03^	2.6131 × 10^+04^	1.2595 × 10^+04^	1.3370 × 10^+04^	9.6597 × 10^+03^	7.8370 × 10^+03^	7.9818 × 10^+03^
	Std	5.7301 × 10^+02^	1.0875 × 10^+03^	1.2023 × 10^+03^	6.6293 × 10^+02^	1.1121 × 10^+03^	1.0194 × 10^+03^	1.5602 × 10^+04^	6.9021 × 10^+02^	4.0110 × 10^+03^	5.5871 × 10^+02^	6.1639 × 10^+02^	7.4755 × 10^+02^
F29	Best	7.3218 × 10^+05^	1.4067 × 10^+08^	8.0392 × 10^+07^	2.8103 × 10^+05^	4.4121 × 10^+07^	1.3377 × 10^+07^	1.5788 × 10^+09^	1.9829 × 10^+08^	3.0501 × 10^+04^	4.1589 × 10^+05^	3.2112 × 10^+05^	3.7711 × 10^+05^
	Mean	3.8950 × 10^+06^	4.6999 × 10^+08^	2.0809 × 10^+08^	4.6513 × 10^+06^	7.4708 × 10^+08^	5.4853 × 10^+07^	8.9374 × 10^+09^	5.1824 × 10^+08^	4.6360 × 10^+08^	2.5357 × 10^+06^	1.1848 × 10^+06^	2.1862 × 10^+06^
	Std	2.1285 × 10^+06^	1.5874 × 10^+08^	1.1684 × 10^+08^	1.4413 × 10^+07^	1.2399 × 10^+09^	3.1361 × 10^+07^	6.0406 × 10^+09^	1.7415 × 10^+08^	1.3305 × 10^+09^	2.4234 × 10^+06^	8.7786 × 10^+05^	1.5811 × 10^+06^

**Table A5:** Comparison of results on CEC 2022 (D = 10).

Function	Metric	MICFOA	CFOA	PEOA	TLBO	COA	ARO	EDO	YDSE	LSHADE	JADE	IDE-EDA	APSM-JSO
F1	Best	3.0000 × 10^+02^	3.1152 × 10^+02^	3.0000 × 10^+02^	3.0000 × 10^+02^	5.2748 × 10^+02^	3.0463 × 10^+02^	3.2714 × 10^+02^	3.0035 × 10^+02^	3.1051 × 10^+02^	3.0000 × 10^+02^	3.0000 × 10^+02^	3.0000 × 10^+02^
	Mean	3.0000 × 10^+02^	5.4346 × 10^+02^	3.0000 × 10^+02^	3.0000 × 10^+02^	3.9789 × 10^+03^	4.9930 × 10^+02^	4.2679 × 10^+02^	3.0239 × 10^+02^	5.9907 × 10^+03^	3.0001 × 10^+02^	3.0000 × 10^+02^	3.0000 × 10^+02^
	Std	1.8047 × 10^−09^	1.8821 × 10^+02^	2.1004 × 10^−05^	3.8672 × 10^−11^	2.7575 × 10^+03^	2.6629 × 10^+02^	6.2803 × 10^+01^	1.6039 × 10^+00^	5.7216 × 10^+03^	2.6941 × 10^−02^	7.5781 × 10^−11^	1.4928 × 10^−13^
F2	Best	4.0000 × 10^+02^	4.0000 × 10^+02^	4.0001 × 10^+02^	4.0000 × 10^+02^	4.0001 × 10^+02^	4.0000 × 10^+02^	4.0719 × 10^+02^	4.0143 × 10^+02^	4.0232 × 10^+02^	4.0000 × 10^+02^	4.0000 × 10^+02^	4.0000 × 10^+02^
	Mean	4.0393 × 10^+02^	4.0362 × 10^+02^	4.1193 × 10^+02^	4.0492 × 10^+02^	4.1263 × 10^+02^	4.1003 × 10^+02^	4.1508 × 10^+02^	4.0617 × 10^+02^	4.0621 × 10^+02^	4.0180 × 10^+02^	4.0427 × 10^+02^	4.0404 × 10^+02^
	Std	3.9364 × 10^+00^	4.2283 × 10^+00^	2.3803 × 10^+01^	1.2888 × 10^+01^	2.0028 × 10^+01^	2.1034 × 10^+01^	6.7789 × 10^+00^	2.1677 × 10^+00^	1.2129 × 10^+00^	2.7481 × 10^+00^	3.5199 × 10^+00^	3.8664 × 10^+00^
F3	Best	6.0000 × 10^+02^	6.0044 × 10^+02^	6.0299 × 10^+02^	6.0000 × 10^+02^	6.0000 × 10^+02^	6.0000 × 10^+02^	6.0972 × 10^+02^	6.0343 × 10^+02^	6.0000 × 10^+02^	6.0000 × 10^+02^	6.0000 × 10^+02^	6.0000 × 10^+02^
	Mean	6.0000 × 10^+02^	6.0234 × 10^+02^	6.1057 × 10^+02^	6.0011 × 10^+02^	6.0848 × 10^+02^	6.0044 × 10^+02^	6.1556 × 10^+02^	6.0715 × 10^+02^	6.0001 × 10^+02^	6.0000 × 10^+02^	6.0002 × 10^+02^	6.0000 × 10^+02^
	Std	4.7003 × 10^−03^	1.8924 × 10^+00^	4.9746 × 10^+00^	1.9030 × 10^−01^	1.1254 × 10^+01^	1.3399 × 10^+00^	4.6486 × 10^+00^	1.9928 × 10^+00^	2.8916 × 10^−02^	3.6799 × 10^−07^	9.1815 × 10^−02^	1.4826 × 10^−03^
F4	Best	8.0199 × 10^+02^	8.0524 × 10^+02^	8.0519 × 10^+02^	8.0366 × 10^+02^	8.1094 × 10^+02^	8.0298 × 10^+02^	8.1852 × 10^+02^	8.1027 × 10^+02^	8.0742 × 10^+02^	8.1189 × 10^+02^	8.0202 × 10^+02^	8.0199 × 10^+02^
	Mean	8.0610 × 10^+02^	8.1020 × 10^+02^	8.1899 × 10^+02^	8.1230 × 10^+02^	8.2925 × 10^+02^	8.1827 × 10^+02^	8.3408 × 10^+02^	8.1979 × 10^+02^	8.2916 × 10^+02^	8.2262 × 10^+02^	8.0661 × 10^+02^	8.0805 × 10^+02^
	Std	2.8591 × 10^+00^	2.8542 × 10^+00^	6.4786 × 10^+00^	5.8051 × 10^+00^	5.8611 × 10^+00^	7.5930 × 10^+00^	6.9085 × 10^+00^	6.3685 × 10^+00^	1.0036 × 10^+01^	5.7893 × 10^+00^	2.4092 × 10^+00^	2.4338 × 10^+00^
F5	Best	9.0000 × 10^+02^	9.0000 × 10^+02^	9.0027 × 10^+02^	9.0000 × 10^+02^	9.0000 × 10^+02^	9.0000 × 10^+02^	9.0690 × 10^+02^	9.0867 × 10^+02^	9.0000 × 10^+02^	9.0000 × 10^+02^	9.0000 × 10^+02^	9.0000 × 10^+02^
	Mean	9.0002 × 10^+02^	9.0076 × 10^+02^	9.1139 × 10^+02^	9.0134 × 10^+02^	1.1375 × 10^+03^	9.0801 × 10^+02^	9.9558 × 10^+02^	9.3257 × 10^+02^	9.0027 × 10^+02^	9.0000 × 10^+02^	9.0011 × 10^+02^	9.0005 × 10^+02^
	Std	8.3988 × 10^−02^	1.3511 × 10^+00^	1.3329 × 10^+01^	2.4043 × 10^+00^	2.3630 × 10^+02^	1.7987 × 10^+01^	7.2321 × 10^+01^	2.2324 × 10^+01^	9.5894 × 10^−01^	0.0000 × 10^+00^	4.0242 × 10^−01^	1.2754 × 10^−01^
F6	Best	1.8010 × 10^+03^	1.9516 × 10^+03^	1.8327 × 10^+03^	1.9533 × 10^+03^	2.0103 × 10^+03^	1.8153 × 10^+03^	1.8651 × 10^+03^	1.8097 × 10^+03^	1.8193 × 10^+03^	1.8171 × 10^+03^	1.8011 × 10^+03^	1.8006 × 10^+03^
	Mean	1.8022 × 10^+03^	3.0993 × 10^+03^	3.9293 × 10^+03^	2.8173 × 10^+03^	4.7688 × 10^+03^	2.4570 × 10^+03^	2.0668 × 10^+03^	1.8298 × 10^+03^	1.1584 × 10^+04^	1.8428 × 10^+03^	1.8104 × 10^+03^	1.8174 × 10^+03^
	Std	7.5328 × 10^−01^	1.4390 × 10^+03^	2.0329 × 10^+03^	1.1916 × 10^+03^	2.1411 × 10^+03^	8.0904 × 10^+02^	1.7620 × 10^+02^	1.3017 × 10^+01^	1.9648 × 10^+04^	1.7922 × 10^+01^	9.7921 × 10^+00^	1.9846 × 10^+01^
F7	Best	2.0000 × 10^+03^	2.0118 × 10^+03^	2.0200 × 10^+03^	2.0012 × 10^+03^	2.0011 × 10^+03^	2.0000 × 10^+03^	2.0327 × 10^+03^	2.0257 × 10^+03^	2.0000 × 10^+03^	2.0000 × 10^+03^	2.0000 × 10^+03^	2.0000 × 10^+03^
	Mean	2.0135 × 10^+03^	2.0305 × 10^+03^	2.0367 × 10^+03^	2.0188 × 10^+03^	2.0216 × 10^+03^	2.0137 × 10^+03^	2.0512 × 10^+03^	2.0386 × 10^+03^	2.0141 × 10^+03^	2.0034 × 10^+03^	2.0126 × 10^+03^	2.0113 × 10^+03^
	Std	9.0843 × 10^+00^	8.0623 × 10^+00^	8.8057 × 10^+00^	7.9251 × 10^+00^	6.7084 × 10^+00^	9.3742 × 10^+00^	9.9008 × 10^+00^	6.4363 × 10^+00^	8.6228 × 10^+00^	6.7865 × 10^+00^	1.0190 × 10^+01^	9.7375 × 10^+00^
F8	Best	2.2004 × 10^+03^	2.2045 × 10^+03^	2.2046 × 10^+03^	2.2010 × 10^+03^	2.2162 × 10^+03^	2.2010 × 10^+03^	2.2242 × 10^+03^	2.2130 × 10^+03^	2.2012 × 10^+03^	2.2033 × 10^+03^	2.2005 × 10^+03^	2.2008 × 10^+03^
	Mean	2.2147 × 10^+03^	2.2232 × 10^+03^	2.2269 × 10^+03^	2.2239 × 10^+03^	2.2403 × 10^+03^	2.2178 × 10^+03^	2.2301 × 10^+03^	2.2256 × 10^+03^	2.2208 × 10^+03^	2.2185 × 10^+03^	2.2152 × 10^+03^	2.2169 × 10^+03^
	Std	8.4494 × 10^+00^	5.5160 × 10^+00^	5.1114 × 10^+00^	6.7854 × 10^+00^	3.6548 × 10^+01^	6.5188 × 10^+00^	1.8582 × 10^+00^	3.9813 × 10^+00^	5.8466 × 10^+00^	8.1690 × 10^+00^	1.0608 × 10^+01^	9.3439 × 10^+00^
F9	Best	2.5293 × 10^+03^	2.5293 × 10^+03^	2.5295 × 10^+03^	2.5293 × 10^+03^	2.5293 × 10^+03^	2.5293 × 10^+03^	2.5295 × 10^+03^	2.5293 × 10^+03^	2.4855 × 10^+03^	2.5293 × 10^+03^	2.5293 × 10^+03^	2.5293 × 10^+03^
	Mean	2.5293 × 10^+03^	2.5301 × 10^+03^	2.5310 × 10^+03^	2.5293 × 10^+03^	2.5391 × 10^+03^	2.5293 × 10^+03^	2.5307 × 10^+03^	2.5294 × 10^+03^	2.4855 × 10^+03^	2.5293 × 10^+03^	2.5293 × 10^+03^	2.5293 × 10^+03^
	Std	4.1369 × 10^−13^	1.8188 × 10^+00^	1.7041 × 10^+00^	5.2736 × 10^−13^	3.7278 × 10^+01^	1.4731 × 10^−02^	8.4272 × 10^−01^	1.0720 × 10^−01^	2.4135 × 10^−02^	1.1942 × 10^−13^	2.2342 × 10^−13^	3.1596 × 10^−13^
F10	Best	2.5003 × 10^+03^	2.5002 × 10^+03^	2.5003 × 10^+03^	2.5002 × 10^+03^	2.5004 × 10^+03^	2.5003 × 10^+03^	2.5006 × 10^+03^	2.5005 × 10^+03^	2.5003 × 10^+03^	2.5003 × 10^+03^	2.5002 × 10^+03^	2.5002 × 10^+03^
	Mean	2.5147 × 10^+03^	2.5258 × 10^+03^	2.5006 × 10^+03^	2.5421 × 10^+03^	2.5570 × 10^+03^	2.5272 × 10^+03^	2.5059 × 10^+03^	2.5007 × 10^+03^	2.5142 × 10^+03^	2.5276 × 10^+03^	2.5438 × 10^+03^	2.5480 × 10^+03^
	Std	3.7288 × 10^+01^	4.6842 × 10^+01^	1.7332 × 10^−01^	5.5786 × 10^+01^	9.5586 × 10^+01^	4.9368 × 10^+01^	2.6825 × 10^+01^	1.1070 × 10^−01^	3.7588 × 10^+01^	5.0236 × 10^+01^	5.4123 × 10^+01^	5.5505 × 10^+01^
F11	Best	2.6000 × 10^+03^	2.7505 × 10^+03^	2.6015 × 10^+03^	2.6000 × 10^+03^	2.6002 × 10^+03^	2.6001 × 10^+03^	2.6772 × 10^+03^	2.7015 × 10^+03^	2.6890 × 10^+03^	2.6000 × 10^+03^	2.9000 × 10^+03^	2.6000 × 10^+03^
	Mean	2.8708 × 10^+03^	2.9072 × 10^+03^	2.7298 × 10^+03^	2.7656 × 10^+03^	2.8446 × 10^+03^	2.7125 × 10^+03^	2.7281 × 10^+03^	2.9037 × 10^+03^	2.8744 × 10^+03^	2.8633 × 10^+03^	2.9000 × 10^+03^	2.8804 × 10^+03^
	Std	9.1866 × 10^+01^	3.5777 × 10^+01^	1.5754 × 10^+02^	1.2656 × 10^+02^	9.7642 × 10^+01^	1.2783 × 10^+02^	2.4905 × 10^+01^	1.2964 × 10^+02^	6.6593 × 10^+01^	1.0662 × 10^+02^	2.5535 × 10^−10^	7.6263 × 10^+01^
F12	Best	2.8586 × 10^+03^	2.8614 × 10^+03^	2.8617 × 10^+03^	2.8614 × 10^+03^	2.8596 × 10^+03^	2.8626 × 10^+03^	2.8625 × 10^+03^	2.8599 × 10^+03^	2.8453 × 10^+03^	2.8645 × 10^+03^	2.8627 × 10^+03^	2.8626 × 10^+03^
	Mean	2.8624 × 10^+03^	2.8644 × 10^+03^	2.8651 × 10^+03^	2.8648 × 10^+03^	2.8671 × 10^+03^	2.8673 × 10^+03^	2.8651 × 10^+03^	2.8626 × 10^+03^	2.8621 × 10^+03^	2.8652 × 10^+03^	2.8650 × 10^+03^	2.8653 × 10^+03^
	Std	1.9027 × 10^+00^	1.3721 × 10^+00^	1.8234 × 10^+00^	1.7680 × 10^+00^	5.5638 × 10^+00^	1.9153 × 10^+00^	3.6877 × 10^+00^	1.4939 × 10^+00^	2.1549 × 10^+01^	6.8116 × 10^−01^	1.6232 × 10^+00^	1.7596 × 10^+00^

**Table A6:** Comparison of results on CEC 2022 (D = 20).

Function	Metric	MICFOA	CFOA	PEOA	TLBO	COA	ARO	EDO	YDSE	LSHADE	JADE	IDE-EDA	APSM-JSO
F1	Best	3.0009 × 10^+02^	4.0502 × 10^+03^	3.0112 × 10^+02^	1.2525 × 10^+03^	1.9293 × 10^+04^	5.9946 × 10^+03^	5.7494 × 10^+03^	1.6566 × 10^+03^	5.2579 × 10^+03^	3.4437 × 10^+03^	3.0879 × 10^+02^	3.0424 × 10^+02^
	Mean	3.1442 × 10^+02^	1.3261 × 10^+04^	3.1464 × 10^+02^	5.5028 × 10^+03^	4.4477 × 10^+04^	1.3293 × 10^+04^	1.1776 × 10^+04^	4.9305 × 10^+03^	6.0472 × 10^+04^	9.1116 × 10^+03^	6.3430 × 10^+02^	4.5865 × 10^+02^
	Std	1.7029 × 10^+01^	4.6885 × 10^+03^	1.7437 × 10^+01^	3.6605 × 10^+03^	1.5100 × 10^+04^	4.7022 × 10^+03^	3.8330 × 10^+03^	3.3082 × 10^+03^	4.2841 × 10^+04^	3.8054 × 10^+03^	3.9426 × 10^+02^	2.8837 × 10^+02^
F2	Best	4.3021 × 10^+02^	4.5808 × 10^+02^	4.3161 × 10^+02^	4.0914 × 10^+02^	4.5018 × 10^+02^	4.5081 × 10^+02^	5.4405 × 10^+02^	4.5582 × 10^+02^	4.1691 × 10^+02^	4.0523 × 10^+02^	4.4490 × 10^+02^	4.1467 × 10^+02^
	Mean	4.6130 × 10^+02^	5.0920 × 10^+02^	4.7406 × 10^+02^	4.5465 × 10^+02^	4.8960 × 10^+02^	4.7232 × 10^+02^	6.9988 × 10^+02^	4.6993 × 10^+02^	4.2229 × 10^+02^	4.5307 × 10^+02^	4.5756 × 10^+02^	4.5084 × 10^+02^
	Std	1.2771 × 10^+01^	4.3208 × 10^+01^	2.9329 × 10^+01^	1.7379 × 10^+01^	4.1685 × 10^+01^	2.0091 × 10^+01^	1.1639 × 10^+02^	1.1724 × 10^+01^	1.5086 × 10^+01^	1.4779 × 10^+01^	1.1444 × 10^+01^	1.3820 × 10^+01^
F3	Best	6.0028 × 10^+02^	6.0635 × 10^+02^	6.2034 × 10^+02^	6.0090 × 10^+02^	6.0793 × 10^+02^	6.0044 × 10^+02^	6.3118 × 10^+02^	6.1504 × 10^+02^	6.0000 × 10^+02^	6.0000 × 10^+02^	6.0005 × 10^+02^	6.0007 × 10^+02^
	Mean	6.0118 × 10^+02^	6.1618 × 10^+02^	6.3433 × 10^+02^	6.0577 × 10^+02^	6.3357 × 10^+02^	6.0467 × 10^+02^	6.4762 × 10^+02^	6.2781 × 10^+02^	6.0037 × 10^+02^	6.0001 × 10^+02^	6.0160 × 10^+02^	6.0154 × 10^+02^
	Std	7.0732 × 10^−01^	6.1928 × 10^+00^	6.4866 × 10^+00^	3.3797 × 10^+00^	1.4538 × 10^+01^	4.0360 × 10^+00^	8.5846 × 10^+00^	5.2672 × 10^+00^	9.6777 × 10^−01^	4.3938 × 10^−02^	1.5471 × 10^+00^	2.1195 × 10^+00^
F4	Best	8.1691 × 10^+02^	8.3544 × 10^+02^	8.3655 × 10^+02^	8.3077 × 10^+02^	8.4635 × 10^+02^	8.2096 × 10^+02^	9.1015 × 10^+02^	8.7101 × 10^+02^	8.2931 × 10^+02^	8.8395 × 10^+02^	8.1376 × 10^+02^	8.1596 × 10^+02^
	Mean	8.2988 × 10^+02^	8.6034 × 10^+02^	8.6200 × 10^+02^	8.6210 × 10^+02^	8.8705 × 10^+02^	8.5175 × 10^+02^	9.4184 × 10^+02^	9.0019 × 10^+02^	9.0834 × 10^+02^	9.0410 × 10^+02^	8.3056 × 10^+02^	8.4065 × 10^+02^
	Std	7.4276 × 10^+00^	1.5840 × 10^+01^	1.0246 × 10^+01^	1.9248 × 10^+01^	1.2705 × 10^+01^	1.5940 × 10^+01^	1.3377 × 10^+01^	1.1948 × 10^+01^	2.8429 × 10^+01^	8.2480 × 10^+00^	9.3096 × 10^+00^	1.9398 × 10^+01^
F5	Best	9.0115 × 10^+02^	9.1803 × 10^+02^	1.3397 × 10^+03^	9.0715 × 10^+02^	2.0073 × 10^+03^	9.8093 × 10^+02^	1.8633 × 10^+03^	1.2717 × 10^+03^	9.0000 × 10^+02^	9.0000 × 10^+02^	9.0345 × 10^+02^	9.0054 × 10^+02^
	Mean	9.1904 × 10^+02^	1.1105 × 10^+03^	1.9738 × 10^+03^	1.0140 × 10^+03^	2.7549 × 10^+03^	1.3342 × 10^+03^	2.7778 × 10^+03^	1.9014 × 10^+03^	9.6983 × 10^+02^	9.0020 × 10^+02^	9.4587 × 10^+02^	9.4025 × 10^+02^
	Std	3.0102 × 10^+01^	1.4929 × 10^+02^	2.8901 × 10^+02^	1.2545 × 10^+02^	3.5767 × 10^+02^	3.0188 × 10^+02^	5.2897 × 10^+02^	5.2377 × 10^+02^	1.8136 × 10^+02^	2.6488 × 10^−01^	3.4544 × 10^+01^	4.3389 × 10^+01^
F6	Best	1.8399 × 10^+03^	1.9661 × 10^+03^	1.9067 × 10^+03^	1.9111 × 10^+03^	1.9812 × 10^+03^	1.8935 × 10^+03^	2.1518 × 10^+05^	4.5176 × 10^+03^	4.4406 × 10^+03^	1.4585 × 10^+04^	1.8862 × 10^+03^	1.8798 × 10^+03^
	Mean	1.8768 × 10^+03^	3.6008 × 10^+03^	4.9765 × 10^+03^	4.8734 × 10^+03^	7.0877 × 10^+03^	4.4741 × 10^+03^	1.1940 × 10^+06^	1.3608 × 10^+04^	6.5886 × 10^+06^	1.0377 × 10^+05^	2.3679 × 10^+03^	2.6657 × 10^+03^
	Std	2.2402 × 10^+01^	1.5033 × 10^+03^	3.8805 × 10^+03^	3.5439 × 10^+03^	5.0709 × 10^+03^	2.9416 × 10^+03^	8.2078 × 10^+05^	8.8680 × 10^+03^	1.0736 × 10^+07^	8.7560 × 10^+04^	1.4847 × 10^+03^	1.8955 × 10^+03^
F7	Best	2.0215 × 10^+03^	2.0633 × 10^+03^	2.0691 × 10^+03^	2.0261 × 10^+03^	2.0426 × 10^+03^	2.0279 × 10^+03^	2.0914 × 10^+03^	2.0746 × 10^+03^	2.0325 × 10^+03^	2.0397 × 10^+03^	2.0228 × 10^+03^	2.0292 × 10^+03^
	Mean	2.0339 × 10^+03^	2.0890 × 10^+03^	2.0965 × 10^+03^	2.0609 × 10^+03^	2.1346 × 10^+03^	2.0636 × 10^+03^	2.1491 × 10^+03^	2.1169 × 10^+03^	2.0734 × 10^+03^	2.0582 × 10^+03^	2.0483 × 10^+03^	2.0513 × 10^+03^
	Std	9.0465 × 10^+00^	1.7029 × 10^+01^	1.9075 × 10^+01^	1.9136 × 10^+01^	8.7158 × 10^+01^	3.7434 × 10^+01^	2.6846 × 10^+01^	2.1533 × 10^+01^	3.0569 × 10^+01^	8.3852 × 10^+00^	2.5447 × 10^+01^	1.7534 × 10^+01^
F8	Best	2.2252 × 10^+03^	2.2279 × 10^+03^	2.2272 × 10^+03^	2.2275 × 10^+03^	2.2269 × 10^+03^	2.2210 × 10^+03^	2.2374 × 10^+03^	2.2337 × 10^+03^	2.2269 × 10^+03^	2.2301 × 10^+03^	2.2224 × 10^+03^	2.2235 × 10^+03^
	Mean	2.2293 × 10^+03^	2.2308 × 10^+03^	2.2730 × 10^+03^	2.2399 × 10^+03^	2.2796 × 10^+03^	2.2321 × 10^+03^	2.2502 × 10^+03^	2.2399 × 10^+03^	2.2419 × 10^+03^	2.2359 × 10^+03^	2.2407 × 10^+03^	2.2420 × 10^+03^
	Std	1.8819 × 10^+00^	2.2576 × 10^+00^	5.3804 × 10^+01^	2.1662 × 10^+01^	6.4713 × 10^+01^	3.1094 × 10^+01^	8.5080 × 10^+00^	4.6391 × 10^+00^	1.2818 × 10^+01^	2.5227 × 10^+00^	3.6077 × 10^+01^	3.6018 × 10^+01^
F9	Best	2.4808 × 10^+03^	2.4852 × 10^+03^	2.4889 × 10^+03^	2.4808 × 10^+03^	2.4808 × 10^+03^	2.4811 × 10^+03^	2.4992 × 10^+03^	2.4823 × 10^+03^	2.4653 × 10^+03^	2.4808 × 10^+03^	2.4808 × 10^+03^	2.4808 × 10^+03^
	Mean	2.4808 × 10^+03^	2.5006 × 10^+03^	2.5069 × 10^+03^	2.4808 × 10^+03^	2.4822 × 10^+03^	2.4873 × 10^+03^	2.5366 × 10^+03^	2.4867 × 10^+03^	2.4661 × 10^+03^	2.4808 × 10^+03^	2.4808 × 10^+03^	2.4808 × 10^+03^
	Std	1.0663 × 10^−02^	1.0958 × 10^+01^	1.3340 × 10^+01^	1.2105 × 10^−06^	3.1724 × 10^+00^	5.0809 × 10^+00^	2.4764 × 10^+01^	2.9118 × 10^+00^	1.4870 × 10^+00^	5.9198 × 10^−04^	1.5249 × 10^−04^	9.1686 × 10^−05^
F10	Best	2.5003 × 10^+03^	2.5004 × 10^+03^	2.5009 × 10^+03^	2.5005 × 10^+03^	2.5007 × 10^+03^	2.5006 × 10^+03^	2.5126 × 10^+03^	2.5014 × 10^+03^	2.4706 × 10^+03^	2.5003 × 10^+03^	2.5004 × 10^+03^	2.5005 × 10^+03^
	Mean	2.6647 × 10^+03^	3.1045 × 10^+03^	2.5330 × 10^+03^	2.8562 × 10^+03^	4.0342 × 10^+03^	2.6495 × 10^+03^	4.2625 × 10^+03^	3.6221 × 10^+03^	3.9481 × 10^+03^	2.7669 × 10^+03^	2.8136 × 10^+03^	2.9792 × 10^+03^
	Std	5.1209 × 10^+02^	1.0441 × 10^+03^	7.2418 × 10^+01^	7.5099 × 10^+02^	1.3463 × 10^+03^	2.0174 × 10^+02^	1.7863 × 10^+03^	1.3666 × 10^+03^	1.2600 × 10^+03^	6.2946 × 10^+02^	6.3601 × 10^+02^	7.0625 × 10^+02^
F11	Best	2.9001 × 10^+03^	3.2496 × 10^+03^	2.9166 × 10^+03^	2.9000 × 10^+03^	2.9324 × 10^+03^	2.6758 × 10^+03^	3.9697 × 10^+03^	4.4273 × 10^+03^	2.9000 × 10^+03^	2.9000 × 10^+03^	2.9000 × 10^+03^	2.9000 × 10^+03^
	Mean	2.9009 × 10^+03^	3.5509 × 10^+03^	2.9553 × 10^+03^	2.9000 × 10^+03^	3.6146 × 10^+03^	2.9386 × 10^+03^	5.0199 × 10^+03^	4.7723 × 10^+03^	3.1880 × 10^+03^	2.9000 × 10^+03^	2.9002 × 10^+03^	2.9023 × 10^+03^
	Std	6.9771 × 10^−01^	1.9881 × 10^+02^	3.9528 × 10^+01^	9.8784 × 10^−03^	1.1149 × 10^+03^	7.4038 × 10^+01^	7.2481 × 10^+02^	2.8021 × 10^+02^	6.2744 × 10^+02^	1.4271 × 10^−09^	2.7787 × 10^−01^	6.7766 × 10^+00^
F12	Best	2.9352 × 10^+03^	2.9482 × 10^+03^	2.9657 × 10^+03^	2.9390 × 10^+03^	2.9494 × 10^+03^	2.9380 × 10^+03^	2.9615 × 10^+03^	2.9553 × 10^+03^	2.9000 × 10^+03^	2.9392 × 10^+03^	2.9375 × 10^+03^	2.9370 × 10^+03^
	Mean	2.9464 × 10^+03^	2.9721 × 10^+03^	3.0219 × 10^+03^	2.9815 × 10^+03^	2.9943 × 10^+03^	2.9911 × 10^+03^	3.0146 × 10^+03^	2.9712 × 10^+03^	2.9000 × 10^+03^	2.9505 × 10^+03^	2.9580 × 10^+03^	2.9607 × 10^+03^
	Std	8.8057 × 10^+00^	1.6327 × 10^+01^	4.7186 × 10^+01^	3.2766 × 10^+01^	3.0682 × 10^+01^	3.2370 × 10^+01^	4.3871 × 10^+01^	9.5614 × 10^+00^	1.1863 × 10^−04^	6.7802 × 10^+00^	1.6094 × 10^+01^	2.0140 × 10^+01^
